# Embryo‐uterine interaction coordinates mouse embryogenesis during implantation

**DOI:** 10.15252/embj.2022113280

**Published:** 2023-07-31

**Authors:** Vladyslav Bondarenko, Mikhail Nikolaev, Dimitri Kromm, Roman Belousov, Adrian Wolny, Marloes Blotenburg, Peter Zeller, Saba Rezakhani, Johannes Hugger, Virginie Uhlmann, Lars Hufnagel, Anna Kreshuk, Jan Ellenberg, Alexander van Oudenaarden, Anna Erzberger, Matthias P Lutolf, Takashi Hiiragi

**Affiliations:** ^1^ European Molecular Biology Laboratory Developmental Biology Unit Heidelberg Germany; ^2^ Faculty of Biosciences University of Heidelberg Heidelberg Germany; ^3^ Institute of Bioengineering, Ecole Polytechnique Fédérale de Lausanne (EPFL) Lausanne Switzerland; ^4^ European Molecular Biology Laboratory, Cell Biology and Biophysics Unit Heidelberg Germany; ^5^ Hubrecht Institute Utrecht The Netherlands; ^6^ EMBL‐EBI, Wellcome Genome Campus Hinxton UK; ^7^ Department of Physics and Astronomy Heidelberg University Heidelberg Germany; ^8^ Institute for the Advanced Study of Human Biology (WPI‐ASHBi) Kyoto University Kyoto Japan; ^9^ Department of Developmental Biology Graduate School of Medicine, Kyoto University Kyoto Japan; ^10^ Present address: Weizmann Institute of Science Rehovot Israel; ^11^ Present address: Institute of Human Biology (IHB) Roche Pharma Research and Early Development Basel Switzerland; ^12^ Present address: Delft Center for Systems and Control Delft University of Technology Delft The Netherlands; ^13^ Present address: Novartis Institutes for BioMedical Research Novartis Pharma AG Basel Switzerland; ^14^ Present address: Veraxa Biotech Heidelberg Germany

**Keywords:** biophysical modeling, embryo, engineering, Implantation, uterus, Cell Adhesion, Polarity & Cytoskeleton, Development

## Abstract

Embryo implantation into the uterus marks a key transition in mammalian development. In mice, implantation is mediated by the trophoblast and is accompanied by a morphological transition from the blastocyst to the egg cylinder. However, the roles of trophoblast‐uterine interactions in embryo morphogenesis during implantation are poorly understood due to inaccessibility *in utero* and the remaining challenges to recapitulate it *ex vivo* from the blastocyst. Here, we engineer a uterus‐like microenvironment to recapitulate peri‐implantation development of the whole mouse embryo *ex vivo* and reveal essential roles of the physical embryo‐uterine interaction. We demonstrate that adhesion between the trophoblast and the uterine matrix is required for *in utero*‐like transition of the blastocyst to the egg cylinder. Modeling the implanting embryo as a wetting droplet links embryo shape dynamics to the underlying changes in trophoblast adhesion and suggests that the adhesion‐mediated tension release facilitates egg cylinder formation. Light‐sheet live imaging and the experimental control of the engineered uterine geometry and trophoblast velocity uncovers the coordination between trophoblast motility and embryo growth, where the trophoblast delineates space for embryo morphogenesis.

## Graphical abstract

The engineering of a uterus‐like microenvironment *ex vivo* uncovers the role of trophoblast adhesion and migration in a key morphological transition of the mouse embryo during implantation.

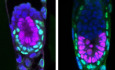

## Introduction

Organismal development is coordinated in space and time across multiple scales. Spatial coordination of mechanics and cell proliferation underlies tissue growth and morphogenesis (Lecuit & le Goff, 2007; Wang & Riechmann, 2007), and the spatial context can determine stem‐cell fate (Rompolas *et al*, 2013). Oscillatory gene expression is linked to tissue‐level morphogenesis and patterning leading to vertebrate segmentation (Palmeirim *et al*, 1997). Spatial coordination can go beyond embryos—extraembryonic tissues regulate the growth and patterning of embryonic tissues via mechanochemical signaling (Chen & Kimelman, 2000; Brennan *et al*, 2001). Furthermore, the maternally provided vitelline envelope physically interacts with embryonic tissues to coordinate their movement (Bailles *et al*, 2019; Münster *et al*, 2019). However, studying the interplay between the triad of embryonic—extraembryonic—maternal tissues in viviparous mammals remains a challenge and its potential role is poorly understood.

**Figure EV1:**
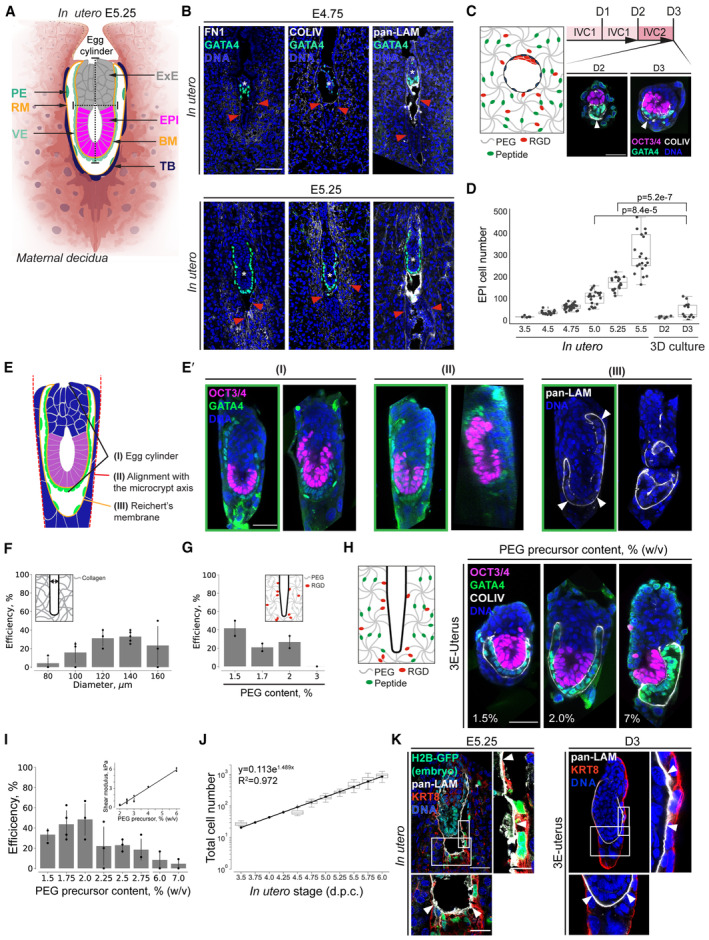
*Ex vivo* Engineering Uterine Environment with topographically patterned hydrogels ASchematic showing the lineages and the layers of extracellular matrix, comprising *in utero* peri‐implantation mouse embryo at E5.25. EPI, epiblast; ExE, extraembryonic ectoderm; TB, trophoblast; PE, parietal endoderm; VE, visceral endoderm; BM, the basal membrane between EPI/ExE and VE; RM, Reichert's membrane; the embryo is surrounded by maternal decidua, the egg cylinder is delineated with dashed lines.BImmunostaining of E4.75 (top) and E5.25 (bottom) pregnant uteri cross sections, showing Fibronectin (FN1, white), Collagen IV (COLIV, white), and Laminin (LAM, white) (from left to right), GATA4 (green), and nuclei (DNA, blue). White asterisks mark the implanted embryos. Red arrowheads point at the uterine ECM.CSchematic of the 3D hydrogel‐embedded embryo culture. *Inset*, Immunostaining of the embryos embedded and cultured 3D inside hydrogel drops until Day 2 (D2) and Day 3 (D3) showing OCT3/4 (magenta), GATA4 (green), and nuclei (DAPI, blue). White arrowheads point at the Reichert's membrane.DComparison of the epiblast (EPI) cell numbers between *in utero* E3.5–E5.5 embryos and embryos embedded and cultured 3D inside hydrogel drops until Days 2–3 (D2–3). *n* = 7 (D2) and *n* = 14 (D3). The midline marks the median, and the boxes indicate the interquartile range. Mann–Whitney's U test *P*‐value.ESchematic of the embryo morphology criteria (I‐III), based on which the efficiency of the *ex vivo* culture is evaluated.E′Immunostaining of 3E‐uterus embryos from Day 3 showing OCT3/4 (magenta), GATA4 (green), Laminin (LAM, white), and nuclei (DNA, blue). The embryos that form egg cylinder (I) show the egg cylinder axis in line with the crypt axis (II), and form Reichert's membrane (III), are considered to be successfully developed (outlined in green; 46%; *n* = 12 of 26, pooled from three independent experiments). White arrowheads point at Reichert's membrane.F3E‐uterus efficiency for embryo culture inside cylindrical crypts with different diameters, calculated across 2 (80 μm), 3 (100 μm), 3 (120 μm), 5 (140 μm), and 3 (160 μm) independent experiments.G3E‐uterus efficiency for embryo culture inside funnel‐shaped microwells made of nonbiodegradable PEG with RGD, calculated across 2 (1.5% PEG concentration), 2 (1.7%), 2 (2%), and 1 (3%) independent experiments.HImmunostaining of 3E‐uterus embryos from Day 3 (D3) grown in 1.5%, 2%, 2.5%, and 7% PEG precursor concentrations (from left to right), showing OCT3/4 (magenta), GATA4 (green), Collagen IV (COLIV, white), and nuclei (DNA, blue).I3E‐uterus efficiency at Day 3 at a 1.5–7% range of PEG precursor content, calculated across 3 (1.5%), 4 (1.75%), 3 (2%), 3 (2.25%), 3 (2.5%), 3 (2.75%), 3 (6%), and 2 (7%) independent experiments. Inset, rheological measurement showing linear relationship between the PEG precursor content (%, w/v) and the Shear modulus (kPa).JTotal cell number (EPI + VE) vs *in utero* developmental stage. The days of 3E‐uterus culture were matched with the *in utero* stages based on the log‐linear regression. Equation of the regression line for the total cell number (EPI and VE) is y = 0.133e1.489x; that for the EPI cell number is y = 0.036e1.617x. *n* = 6 (E3.5), *n* = 21 (E4.5), *n* = 28 (E4.75), *n* = 20 (E5.0), *n* = 20 (E5.25), *n* = 21 (E5.5), *n* = 21 (E5.75) and 22 (E6.0). Y scale, log 10.KImmunostaining of E5.25 pregnant uterus cross section (left) and 3E‐uterus embryo from Day 3 (right) showing H2B‐GFP (marks the embryo in green), Cytokeratin 8 (KRT8, red), pan‐Laminin (pan‐LAM, white), and nuclei (DNA, blue). Right, 4× zoom; bottom, 2× zoom. White arrowheads point at Reichert's membrane. Scale bars, 50 μm, 100 μm (A), 12.5 μm (J, zoom‐in).

By establishing the embryo‐maternal interaction, implantation represents a critical developmental stage in mammalian species. Mammalian development begins with generating extraembryonic lineages, trophectoderm (TE), and primitive endoderm (PrE), in addition to the embryonic epiblast (EPI) in the blastocyst. In mice, at embryonic day (E) 4.5, the TE differentiates into EPI‐attaching polar TE (pTE) and EPI‐distant mural TE (mTE), which adheres to the uterine wall and initiates implantation. The former generates the extraembryonic ectoderm (ExE) tissue and the latter differentiates into giant trophoblast (GT), while EPI and ExE proliferate and elongate to form an “egg cylinder” (Fig EV1A; Movie EV1). The extraembryonic lineages neighboring the EPI, ExE, and visceral endoderm (VE) derived from PrE, play a key role in embryonic growth, patterning, and body axis formation via signaling (Brennan *et al*, 2001; Rodriguez *et al*, 2005; Ichikawa *et al*, 2022). In addition to these interactions between embryonic and extraembryonic tissues postimplantation, the maternal uterine tissues establish a unique context for mammalian development. In particular, while the placenta provides nutritional support for embryonic growth postimplanation, the potential role of embryo‐uterus interactions in peri‐implantation embryogenesis remains largely unexplored (Mesnard *et al*, 2004; Hiramatsu *et al*, 2013).

*Ex vivo* culture of peri‐implantation mouse embryos has been developed both in 2D (Hsu, 1972, 1973; Morris *et al*, 2012; Bedzhov *et al*, 2014) and 3D (Govindasamy *et al*, 2021; Ichikawa *et al*, 2022). However, the peri‐implantation development of the extraembryonic trophoblast together with the embryonic egg cylinder remains challenging to recapitulate *ex vivo* and study with the available culture systems. The 2D culture induces embryo adhesion on the 2D surface which disrupts Reichert's membrane and embryo morphogenesis, and the 3D culture so far has required removal of the mTE to release tension in the pTE enabling invagination and formation of the ExE. While our recent data suggested the role of embryo‐uterus interaction in tension release *in utero* (Ichikawa *et al*, 2022), the exact mechanism awaited development of an *ex vivo* system that recapitulates the uterine environment and embryo‐uterus interaction upon implantation.

Bioengineering approaches are generally used to emulate biochemical and mechanical properties of *in vivo* tissues and identify microenvironmental characteristics necessary and/or sufficient for tissue morphogenesis and patterning. For example, biomaterials engineering and microfabrication provide powerful tools for such *ex vivo* modeling of the native 3D environment (Lutolf & Hubbell, 2005; Vianello & Lutolf, 2019). Chemically defined matrices, such as those based on poly(ethylene glycol) (PEG), provide tunability, robustness and reproducibility for state‐of‐the‐art mechanobiological studies (Seliktar, 2012; Caliari & Burdick, 2016; Gjorevski *et al*, 2016; Qazi *et al*, 2022). These techniques can be combined with live microscopy to gain mechanistic insights into tissue morphogenesis and patterning (Gjorevski *et al*, 2022).

In this study, we develop an engineered uterus that reconstitutes key properties of the mouse uterine environment at implantation, in order to dissect the potential role and mechanisms of embryo‐uterus interaction and tissue coordination during mouse peri‐implantation development. The controlled geometry of the engineered environment permits a quantitative theoretical description of the process.

## Results

### An engineered uterus supports the development of the whole mouse embryo from blastocyst to egg cylinder

**Figure 1:**
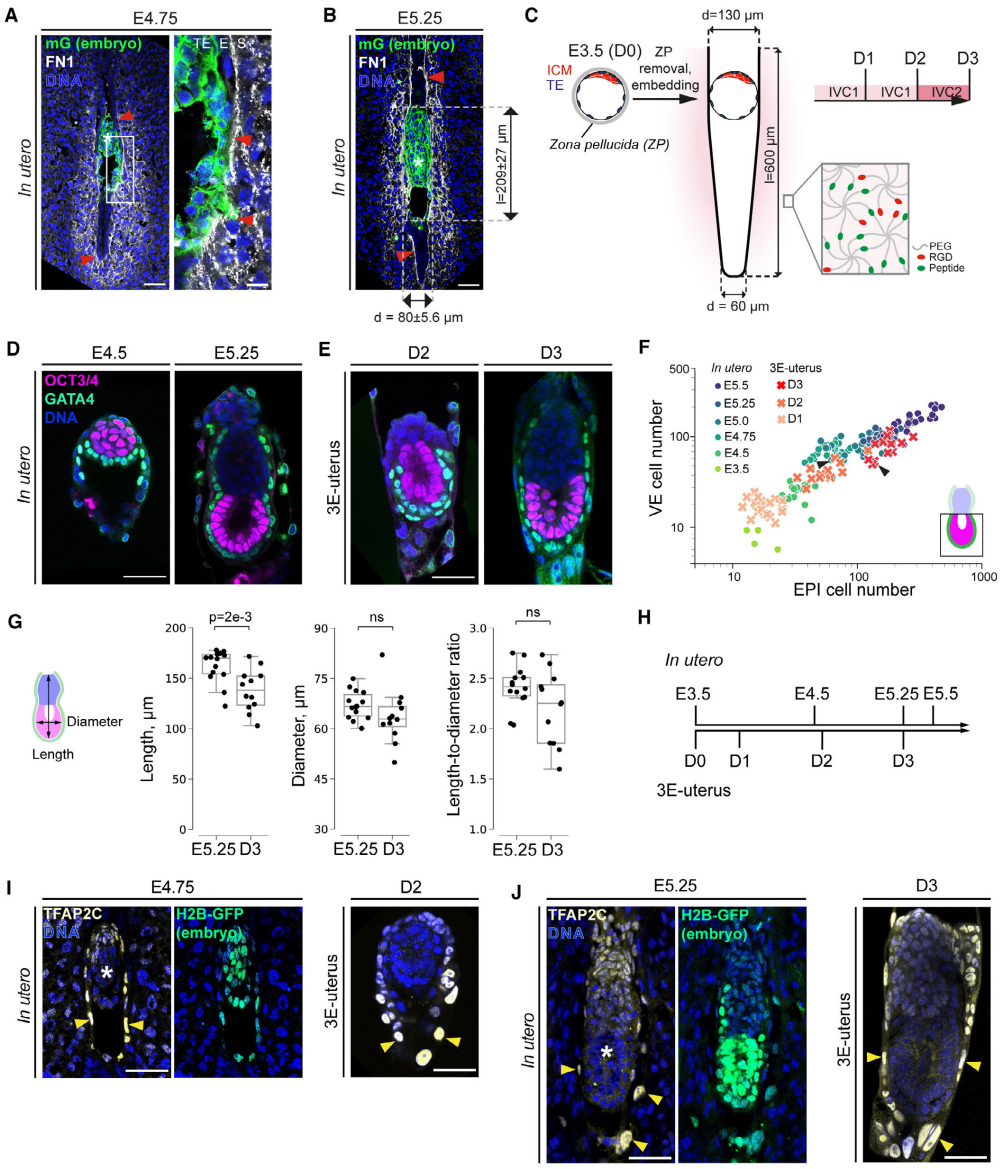
Engineered uterus supports the development of the whole embryo from blastocyst to egg cylinder A, BImmunostaining of pregnant uteri cross sections at E4.75 (A) and E5.25 (B) showing mGFP (marks the embryo in green), Fibronectin 1 (FN1, white), and nuclei (DNA, blue). Red arrowheads point at the uterine ECM. (A) right, 4× zoom into the interface between trophectoderm (TE), the uterine epithelium (E), and stroma (S). (B), *In utero* length (l) and diameter (d) of E5.25 embryos.CSchematic of peri‐implantation embryo culture. Embryos are recovered at E3.5, treated with Tyrode's solution to remove *Zona pellucida* (ZP), and embedded into the crypts on the day of recovery (D0). IVC1 and ICV2 stand for “*In Vitro* Culture” medium 1 and 2, respectively. Inset, schematic of the hydrogel composition. 8‐arm Poly(ethylene glycol) (PEG, gray) molecules, cross‐linked via metalloprotease‐cleavable peptides (Peptide, green), and functionalized with RGDSPG peptide (Arg‐Gly‐Asp‐Ser‐Pro‐Gly, “RGD,” red).D, EImmunostaining of *in utero* E4.5 and E5.25 embryos (D) and 3E‐uterus Day 2 and Day 3 embryos (E) showing OCT3/4 (magenta), GATA4 (green), and nuclei (DAPI, blue).FNumbers of epiblast (EPI) cells (x‐axis) vs visceral endoderm (VE) cells (y‐axis) that cover epiblast (shown on the bottom right scheme) in the embryos developed *in utero* until E5.5 (Ichikawa *et al*, 2022; E3.5–E5.5) and the embryos successfully developed by 3E‐uterus until Day 3 (D1–3). *n* = 5 (E3.5), *n* = 21 (E4.5), *n* = 28 (E4.75), *n* = 20 (E5.0), *n* = 20 (E5.25), *n* = 21 (E5.5); *n* = 20, two replicates pooled (D1), *n* = 13 of 28, three replicates pooled (D2), *n* = 12 of 26, three replicates pooled (D3). X/Y scale, log 10, arrows point to the representative 3E‐uterus embryos shown in (E).GLeft to right, Egg cylinder's length, diameter, and the length‐to‐diameter ratio between embryos developed *in utero* until E5.25 and 3E‐uterus embryos from Day 3 (D3). *n* = 14 and 12, respectively. Data points correspond to individual embryos, midline marks the median, and boxes indicate interquartile range. Student's *t*‐test and the Mann–Whitney's U test *P*‐values.HCell number‐based correspondence between *in utero* and 3E‐uterus embryo development.I, J(I) Immunostaining of E4.75 pregnant uterus cross section (left) and 3E‐uterus embryo from Day 2 (right) showing H2B‐GFP (marks the embryo in green), TFAP2C (yellow), and nuclei (DNA, blue). (J) Immunostaining of pregnant uterus cross section (left, E5.25) and 3E‐uterus embryo (right, Day 3), showing H2B‐GFP (marking the embryo, green), TFAP2C (yellow), and nuclei (DNA, blue). Yellow arrowheads mark differentiated trophoblast cells. White asterisks indicate the epiblast of the implanted embryos. Data information: Scale bars, 50 μm, 12.5 μm (A, right). Source data are available online for this figure.

*In utero*, the trophoblast progressively reaches the extracellular matrix (ECM) underlying the uterine epithelium between embryonic day 4.75 (E4.75) and E5.25 (Fig 1A and B; Li *et al*, 2015). However, using synthetic hydrogels that mimic ECM, we found that a 3D isotropic environment fails to support mouse blastocysts through peri‐implantation development resulting in their growth retardation (Fig EV1C and D; Ichikawa *et al*, 2022). On the contrary, the blastocyst culture on the 2D surface, such as a glass bottom dish, disrupts Reichert's membrane, leading to independent growth of trophoblast and epiblast lineages. Notably, the deposition of ECM components around implanting embryos at E4.75 and E5.25 consistently evidences a crypt‐shaped tissue geometry (Figs 1A and B, and EV1B; Farrar & Carson, 1992). Using microfabrication, we thus applied a topographical 3D modification of the hydrogel to generate the elongated crypt shape that the mouse uterus acquires around implanting embryos (Burckhard, 1901; Cha *et al*, 2014) with high precision (Nikolaev *et al*, 2020; Gjorevski *et al*, 2022; Fig 1C; Movie EV2).

The optimal crypt dimension in 3E‐uterus (*ex vivo* engineered uterine environment with 3D geometrically patterned hydrogels) was determined by the efficiency of the *ex vivo* culture, as judged by the embryo morphology (Fig EV1E and F). A diameter gradient was introduced to accommodate variability in blastocyst size (Fig 1C). An addition of metalloprotease (MMP)‐sensitive cross‐linking peptides resulted in hydrogel biodegradability and a higher developmental efficiency compared with the nonbiodegradable matrix (Fig EV1G–I). Among the 10‐fold range of the shear moduli generated by 1.5–7% biodegradable PEG precursor content, 1.5–2% PEG generated the shear modulus at 100–300 Pa, which is in the stiffness range of the E5.5 mouse decidua (Govindasamy *et al*, 2021) and resulted in the highest developmental efficiency (Fig EV1H and I).

Under these conditions, E3.5 blastocysts reached epiblast (EPI) and visceral endoderm (VE) cell numbers as well as the diameter and the length‐to‐diameter ratio of the egg cylinder comparable to E5.25 embryos developed *in utero* (Fig 1D–G; Movie EV2). 3E‐uterus reproduces E5.25 egg cylinder formation with a 46% efficiency after 3 days of culture (*n* = 12 of 26 embryos; Fig 1D–G), with developmental progression slowed down during the first 2 days (Figs 1H and EV1J). Laminin‐rich Reichert's membrane, connected to the basal membrane of the egg cylinder, successfully formed in 77% of the embryos (*n* = 20 of 26 embryos; Figs 1I and J, and EV1K). In these embryos, the inner side of the Reichert's membrane contained GATA4‐positive cells, corresponding to the parietal endoderm (PE), whereas Tfap2C‐ and Krt8‐positive trophoblast (TB) formed on the outer side with enlarged nuclei (Figs 1J and EV1K).

**Figure 2:**
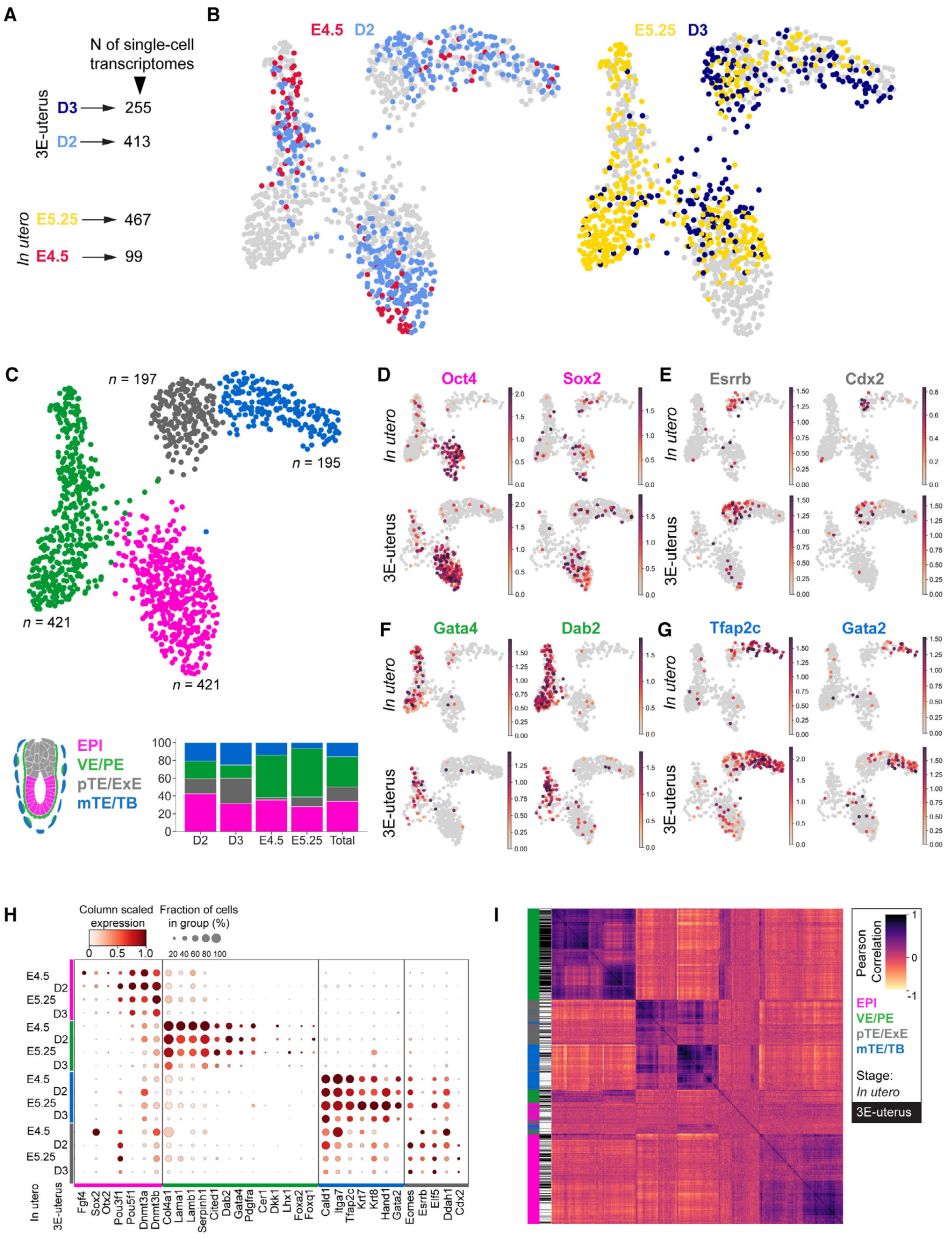
Single‐cell transcriptional profiling of peri‐implantation mouse development *in utero* and in 3E‐uterus ASingle cells were collected and sequenced from 6 to 10 embryos for each experimental condition (E4.5, E5.25, Day 2, Day 3; red, yellow, light and dark blue, respectively) from two independent replicates (litters, *N* = 8 in total). After quality‐based filtering, in total 1,234 transcriptomes were used for further analysis.BThe UMAP of single‐cell transcriptomes colored by the *in utero* and 3E‐uterus experimental conditions shown on the same graph: left, E4.5 (red) and D2 (light blue); right, E5.25 (yellow) and D3 (dark blue).CThe UMAP colored by the clustering outcome (Leiden, Traag *et al*, 2019), identifying epiblast (EPI, pink; *n* = 421), visceral and parietal endoderm (VE/PE, green; *n* = 421), polar trophectoderm/extraembryonic ectoderm (pTE/ExE, gray; *n* = 197), and mural trophectoderm/trophoblast (mTE/TB, blue; *n* = 195) cells. Bottom, percentage of the identified cell types across the experimental conditions.D–GUMAPs for *in utero* (top, *n* = 566) and 3E‐uterus (bottom, *n* = 668) cells colored by the normalized gene expression of Oct3/4 and Sox2 (EPI, D), Esrrb and Cdx2 (pTE/ExE, E), Gata4 and Dab2 (VE/PE, F), Tfap2c and Gata2 (mTE/TB, G).HDotplot showing quantification of the gene expression within the cell groups arranged by the experimental condition (E4.5, E5.25, D2, and D3) and cell type (EPI, magenta; VE/PE, green; mTE/TB, blue; pTE/ExE, gray), corresponding to y‐axis. The normalized gene expression level is denoted by the color of each dot, along with the fraction of the cell number in the group where marker gene expression was detected (dot size). X‐axis shows marker gene names. The plot indicates lineage‐specific expression of the marker genes and comparable gene expression levels between *in utero* (E4.5 and E5.25) and 3E‐uterus (Day 2 and Day 3) conditions across different cell types (EPI, magenta; VE/PE, green; mTE/TB, blue; pTE/ExE, gray).IHeatmap of unsupervised clustering of all *in utero* (black) and all 3E‐uterus (white) cells based on Pearson correlation using the first 50 principal component values from the expression of all protein‐coding genes. The plot shows that the cells from *in utero* and 3E‐uterus cluster predominantly based on the cell type rather than the sample origin. Source data are available online for this figure.

**Figure EV2:**
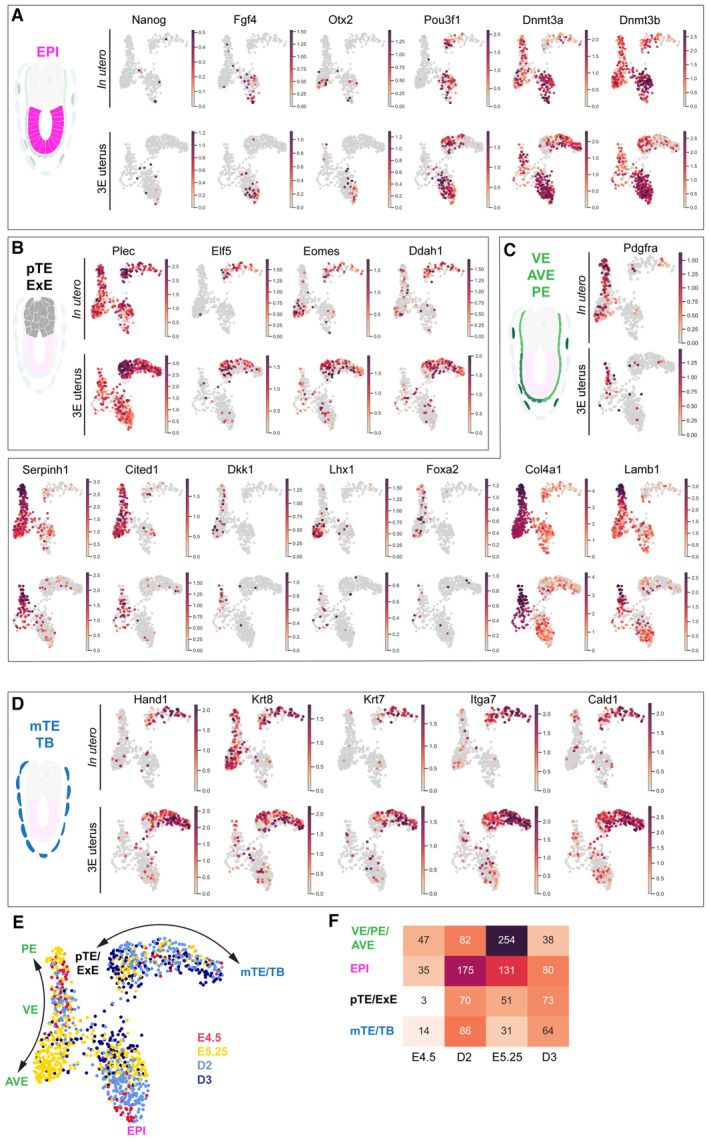
Expression of the lineage marker genes A–DUMAPs colored by the normalized expression of epiblast (A, EPI), polar trophectoderm/extraembryonic ectoderm (B, pTE/ExE), visceral, anterior visceral, and parietal endoderm (C, VE/AVE/PE), and mural trophectoderm/trophoblast (D, mTE/TB) across *In utero* (top, *n* = 566) and 3E‐uterus (bottom, *n* = 668) cells.EThe UMAP colored by the experimental conditions: 3E‐uterus (D2, light blue; D3, dark blue) and *in utero* (E.4.5, red; E5.25, yellow), total *n* = 1,234.FThe numbers of single‐cell transcriptomes per experimental condition and cell type.

We then evaluated *ex vivo* development by a comprehensive gene expression analysis. We collected single cells and performed RNA sequencing (scRNA‐seq) from whole embryos at Day 2 and Day 3 of 3E‐uterus as well as those developed *in utero* to E4.5 and E5.25, resulting in total of 1,234 single‐cell transcriptomes (Fig 2A; Appendix Fig S1). Overall, cells from 3E‐uterus and from *in utero* clustered together, indicating transcriptional similarity between Day 2 and E4.5 as well as between Day 3 and E5.25 (Fig 2B). We annotated four distinct clusters, based on the known marker gene expression, which correspond to epiblast (EPI, Pou3f1, Pou5f1, Sox2, Fgf4, Otx2, Dnmt3a, and Dnmt3b; Figs 2C and D, and EV2A), polar trophectoderm/extraembryonic ectoderm (pTE/ExE, Cdx2, Eomes, Elf5, Esrrb, Ddah1, and Plec; Figs 2C and E, and EV2B), visceral/parietal endoderm (VE/PE, Gata4, Pdgfra, Dab2, Serpinh1, Cited1, and Foxa2; Figs 2C and F, and EV2C), and mural trophectoderm/trophoblast (mTE/TB, Tfap2c, Krt7, Krt8, Gata2, Hand1, Cald1, and Itga7 (Klaffky *et al*, 2001); Figs 2C and G, and EV2D). These four cell types were found in all embryo stages and developing conditions (Figs 2C and EV2E and F), although tissue‐specific dissociation conditions likely contributed to a variable representation of some lineages. EPI cells from E4.5 embryos clustered with those from 3E‐uterus Day 2 and E5.25 cells with Day 3 cells (Appendix Fig S2A), suggesting that the *ex vivo* culture in 3E‐uterus does not perturb the pluripotency exit program (Appendix Fig S2B; Shahbazi *et al*, 2017). Cell clustering using either marker genes or genome‐wide transcription showed high similarity and no evident separation based on *in utero*/3E‐uterus cell origin (Fig 2H and I), indicating that the cells from 3E‐uterus embryos are transcriptionally indistinguishable from the cells from *in utero*, and cluster in a cell‐type‐specific manner.

Altogether, these results indicate that our new method of *ex vivo* culture with an engineered uterus recapitulates *in utero* peri‐implantation development of the whole mouse embryo with the trophoblast, providing an opportunity for mechanistic studies of embryo implantation.

### Trophoblast cells lose polarity and acquire motility upon adhesion, which is essential for peri‐implantation mouse development

**Figure 3:**
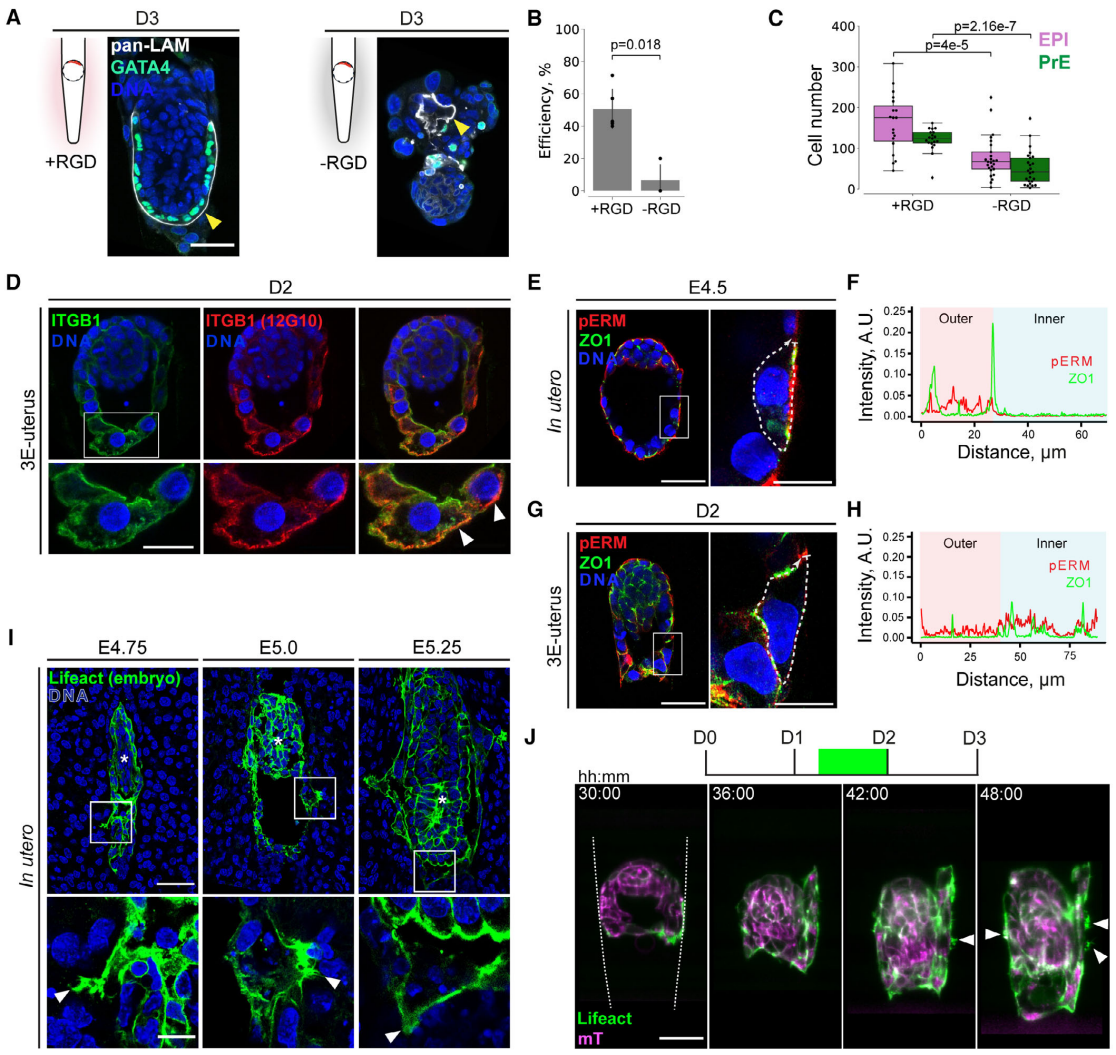
Trophoblast cells lose polarity and acquire motility upon adhesion, which is essential for peri‐implantation development AImmunostaining of embryos cultured in 3E‐uterus with RGD (left) and without RGD (right) for 3 days showing pan‐Laminin (pan‐LAM, white), GATA4 (green), and nuclei (DNA, blue). Yellow arrowheads point at the Reichert's membrane.BDevelopmental efficiency of 3E‐uterus with RGD and without RGD. Dots correspond to efficiency values in experimental replicates (*N* = 5 and 3, respectively). Error bars mark SD, Student's *t*‐test *P*‐value.CNumbers of epiblast (OCT4+, EPI) and primitive endoderm (GATA4+, PrE) cells in all embryos grown in 3E‐uterus with RGD (*n* = 19, pooled from three biological replicates) and without RGD (*n* = 25, pooled from three biological replicates) at Day 3 of 3E‐uterus. The midline marks the median, the boxes indicate the interquartile range, and the whiskers extend maximum ± 1.5x interquartile range.DImmunostaining of 3E‐uterus embryo from Day 2 showing integrin beta 1 (ITGB1, green), active ITGB1 (12G10, red), and nuclei (DNA, blue). Bottom, 2× zoom. White arrowheads point at the apical surface of the trophoblast (TB) cells.E–H(E, G) Immunofluorescence of the embryo grown *in utero* until E4.5 and 3E‐uterus embryo from Day 2 (G) showing ZO‐1 (green), phosphor‐Ezrin/Radixin/Moesin (pERM, red), and nuclei (DNA, blue). Right, 4x zoom into the mural TE (mTE) cell. (F, H) Corresponding intensity profile plots for ZO1 and pERM signals along the cell surface outlined in (E) and (G), right.Ileft to right, Immunostaining of the E4.75, E5.0, and E5.25 pregnant uteri cross sections showing Lifeact‐GFP (marking the embryos in green) and nuclei (DNA, blue). Bottom, 4× zoom. White arrowheads point at the mural trophectoderm (mTE)/TB membrane protrusions. White asterisks indicate the epiblast of the implanted embryos.JTime‐lapse images of the developing Lifeact‐GFP (green);mTmG (magenta) embryo. The crypt surface is outlined. t = 00:00, hours: minutes from recovery at E3.5. Data information: Scale bars, 50 μm, 25 μm (2× zoom), 12.5 μm (4× zoom). Source data are available online for this figure.

**Figure EV3:**
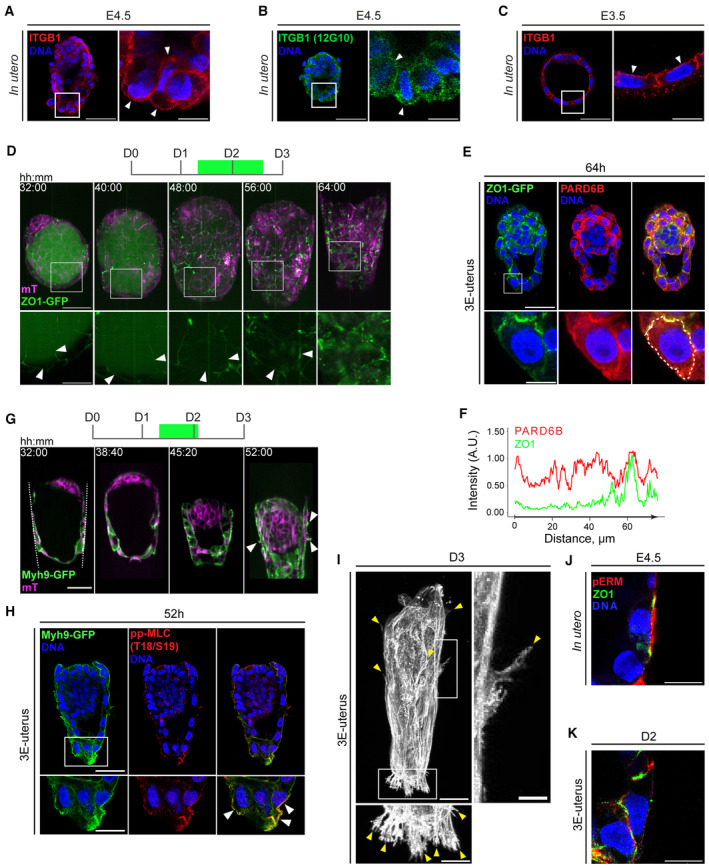
Characterization of trophoblast cell polarity and cytoskeletal dynamics A, BImmunofluorescence of E4.5 embryos showing nuclei (DNA, blue), integrin beta 1 (ITGB1, red) (A), and active ITGB1 (12G10, green) (B). Right, 4× zoom‐ins. Arrowheads point to the apicobasal integrin localization in mural TE.CImmunofluorescence of the blastocyst‐stage embryo (E3.5) showing integrin beta 1 (ITGB1, red), and nuclei (DAPI, blue). Right, 4× zoom. Arrowheads point to the basal integrin localization in TE.D3D projections of time‐lapse images of the developing ZO1‐GFP (green);mTmG (magenta) embryo. Bottom, 2.5× zoom into the TE cell; white arrowheads mark cell–cell interfaces.EImmunofluorescence of the 3E‐uterus embryo after live imaging, simultaneously stained for ZO1‐GFP (green) PARD6B (red), and nuclei (DNA, blue). From left to right, ZO1‐GFP, PARD6B, composite image channels. Bottom, 4× zoom of the TB cell.FIntensity profile of ZO1 and PARD6B signals along the cell surface outlined in (E, bottom), including apical and basolateral regions.GTime‐lapse images of the developing Myh9‐GFP (green);mTmG (magenta) embryo. The crypt surface is outlined.HImmunofluorescence of the 3E‐uterus embryo after live imaging showing Myh9‐GFP (green) phosphor‐MLC (T18/S19) (red), and nuclei (DNA, blue). Bottom, 2× zoom. White arrowheads point at the apical TB cell surface.IImmunofluorescence of Day 3 3E‐uterus embryo, showing maximum Z‐projection of F‐actin signal (white). Bottom, 2× zoom; right, 4× zoom. Yellow arrows mark trophoblast cell membrane protrusions. Invasive trophoblast cell protrusions at least 10 μm deep inside the biodegradable LDTM PEG matrix are consistently observed in 86% of all WT embryos at the Day 3 of 3E‐uterus.J, Kimmunofluorescence of the mural TE (mTE) cell of the embryo grown *in utero* until E4.5 (left) and 3E‐uterus embryo from Day 2 (right) showing ZO‐1 (green), phosphor‐Ezrin/Radixin/Moesin (pERM, red), and nuclei (DNA, blue) without the outline, corresponding to Fig 3E and G. t = 00:00, Hours: Minutes from recovery at E3.5. Scale bars, 50 μm, 25 μm (2× zoom), 20 μm (2.5× zoom), 12.5 μm (4× zoom).

As we established a geometrical and mechanical context for the engineered uterus, we then examined the dependence of *ex vivo* embryo development on its key biochemical characteristics. Remarkably, the developmental efficiency significantly dropped in the absence of RGD (Fig 3A–C), suggesting that integrin‐mediated adhesion of the embryo to the uterine wall is required for peri‐implantation mouse development. In agreement with this, the integrin beta 1 subunit and its active form are enriched at the basal as well as the apical side of the mTE/TB cells that mediate adhesion of the embryo to the uterine wall (Figs 3D and EV3A and B; Sutherland *et al*, 1993; Govindasamy *et al*, 2021), in contrast to its basal localization in E3.5 blastocysts (Fig EV3C; Kim *et al*, 2022).

To understand the mechanisms of mTE cell reaction upon implantation, we further characterized mTE/TB cellular dynamics at the subcellular level. First, we examined how the apical side of mTE cells, which initially lacks integrin beta 1 (Fig EV3C), could adhere and mediate migration in the uterine ECM. Immunofluorescence staining of 3E‐uterus embryos at D2 showed localization of the apical marker, pERM, at the basolateral surface and of the basal marker, integrin beta 1, at the apical surface (Fig 3E–H). Localization of the tight‐junction marker, ZO‐1, also becomes disorganized during 3E‐uterus culture (Figs 3E–H and EV3D–F; Movie EV3). These data show that mTE cells lose cell polarity upon adhesion to the uterine matrix.

To investigate mTE/TB cell morphology upon implantation *in utero*, we systematically examined mTE/TB cells in their native uterine tissue context throughout the implantation stages. To distinguish embryo‐derived cells in the uterine tissues, we crossed Lifeact‐GFP (Riedl *et al*, 2010) males with WT females, so that only embryo‐derived cells have GFP expression within the tissue sections. mTE/TB cells of the embryo formed actin‐rich filopodia/lamellipodial cell membrane protrusions into the uterine tissue at E4.75 (Fig 3I, left). TB cell protrusions were observed along the mesometrial/antimesometrial (M/AM) axis, as well as laterally at E5.0 and E5.25 (Fig 3I, middle, right). These prominent filopodia/lamellipodia are in agreement with the migration of mTE/TB cells *in utero*. The Itgb1 genetic knockout and immunofluorescence imaging of the pregnant uterine sections at E4.75 revealed developmental failure (all 10 identified embryos had Itgb1 expression, *N* = 2 litters). At E4.5, we found two out of 19 embryos lacking Itgb1 expression and *in utero* cell protrusions associated with implantation (*N* = 3 litters; Appendix Fig S3). Furthermore, InVi‐SPIM light‐sheet microscopy (Strnad *et al*, 2016) of actomyosin dynamics in Lifeact‐GFP;mTmG and Myh9‐GFP;mTmG (Zhang *et al*, 2012) embryos (Movies [Link], [Link]) showed enrichment at the adhesion side of the mTE, followed by lamellipodia and filopodia formation at the TB migration front along the crypt axis and laterally inside the hydrogel (Figs 3J and EV3G–I). These data indicate that integrin‐mediated adhesion to the uterine matrix induces mTE/TB cells to lose polarity and migrate.

### A droplet‐wetting process can explain the coupling between trophoblast‐uterine interfacial dynamics and morphogenesis

**Figure 4:**
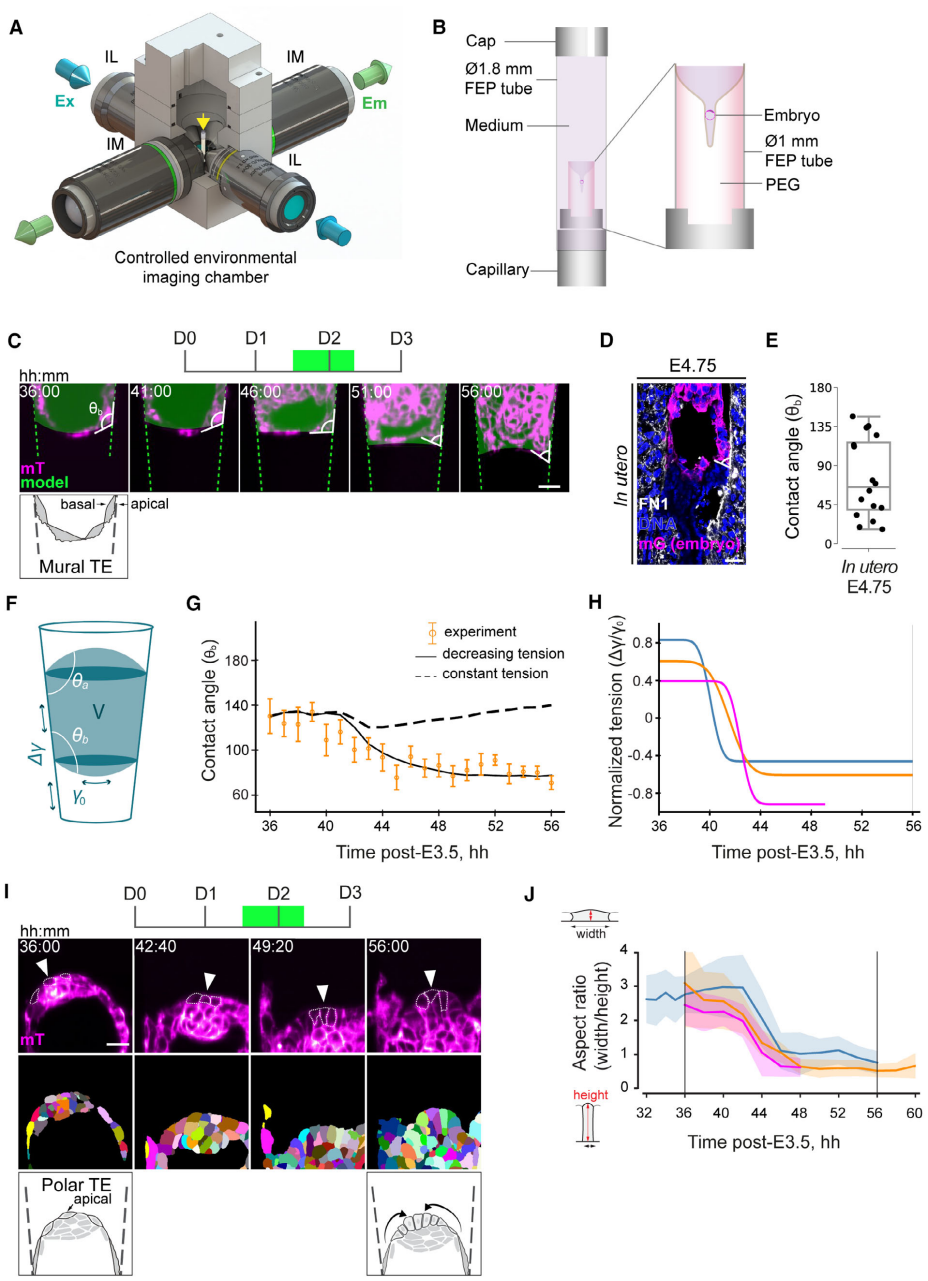
Droplet‐wetting process can explain embryo‐uterus interactions upon implantation Schematic of the MuVi‐SPIM setup with two low‐NA illumination objective lenses (IL), two high‐NA imaging objective lenses (IM), and the controlled environmental imaging chamber with the sample holder (yellow Arrow).Schematic of the sample holder. The outer FEP tube (∅1.8 mm, l = 25 mm) is mounted on top of the sealed glass capillary and filled with IVC medium. The inner FEP tube (∅1 mm, l = 3 mm) contains the crypt and is supported by the PDMS holder from the bottom. The embryo is mounted from the top. The outer FEP tube is closed with the PDMS cap with ∅0.6 mm opening for the gas exchange.Time‐lapse images of the mural TE (mTE) in the mTmG (magenta) developing embryo. The fitted droplet model (embryo) and the frustum shape (crypt) are in green; an exemplar contact angle (θ) between mTE and the crypt surface is shown. Apical and basal sides of mural TE are marked with arrows.Immunostaining of the E4.75 pregnant uterus cross section showing mGFP (marks the embryo in magenta), Fibronectin 1 (FN1, white), and nuclei (DNA, blue). An exemplar contact angle between mTE and the uterine basal membrane is shown.
*In utero* contact angle values from six E4.75 embryos collected from three biological replicates, measured in 1–2 cross sections. The midline marks the median, the boxes indicate the interquartile range, and the whiskers extend maximum ± 1.5× interquartile range.Schematic of the active droplet in a frustum‐shaped confinement; θ_a_ and θ_b_ denote top and bottom contact angles, respectively.Simulated contact angle dynamics for constant tension (dashed line) and decreasing tension (solid line) with experimental data (in orange). Error bars denote SEM. See also Fig EV4H and Appendix Fig S4.Inferred dynamics of the normalized embryo‐substrate interfacial tension difference. Colors correspond to independent experiments.Top, time‐lapse images of the polar TE (pTE) in mTmG (magenta) developing embryo. Exemplar pTE cells are marked with arrowheads, cell perimeter is outlined. Middle, corresponding images of the 3D cell membrane segmentation. Bottom, the schematic of pTE cell columnarization and invagination. Apical side of polar TE is marked with an arrow.Dynamics of the width‐to‐height aspect ratio of the pTE cells. Colors correspond to independent experiments (same as H). Average values across 15–20 cells per time point (solid line) and standard deviations (shaded area) are shown. t = 00:00, hours: minutes from recovery at E3.5. Data information: Scale bars, 25 μm. Source data are available online for this figure.

Next, to characterize the tissue‐scale change upon implantation at a cellular resolution, we combined 3E‐uterus with the multiview light‐sheet microscopy (MuVi‐SPIM; Krzic *et al*, 2012; McDole *et al*, 2018) by implementing a controlled environment to the sample chamber (Fig 4A and B). Live imaging showed distinct changes in the position and contact angle of the embryo in relation to the 3E‐uterus surface (Fig 4C), in agreement with the *in utero* observations (Fig 4D and E). Our findings on the enrichment of integrins in TB cells (Figs 3D and EV3A–C) led us to hypothesize that an active adaptation of embryo‐uterus adhesion may explain the observed evolution of the embryo shape as described by the physics of a droplet‐wetting process (de Gennes, 1985; Douezan *et al*, 2011).

To identify the key physical drivers of implantation dynamics, we sought a theoretical description that is as simple as possible while both recapitulating and predicting the experimental measurements. To this end, we developed a minimal model of the embryo as a fluid droplet, confined within a conical frustum representing the 3E‐uterus. The droplet has different interfacial tensions with the substrate and the medium, with γ_0_ denoting the tension of the droplet–medium interface and Δγ the tension difference between droplet‐substrate and substrate‐medium interfaces, and it is subject to a Laplace pressure ∆P that acts as a Lagrange multiplier to the imposed droplet volume V. We did not impose any asymmetries or gradients in the physical properties of the droplet otherwise or considered complex fluid properties arising from an active migratory polarity of cells (as, e.g., in Pérez‐González *et al*, 2019).

**Figure EV4:**
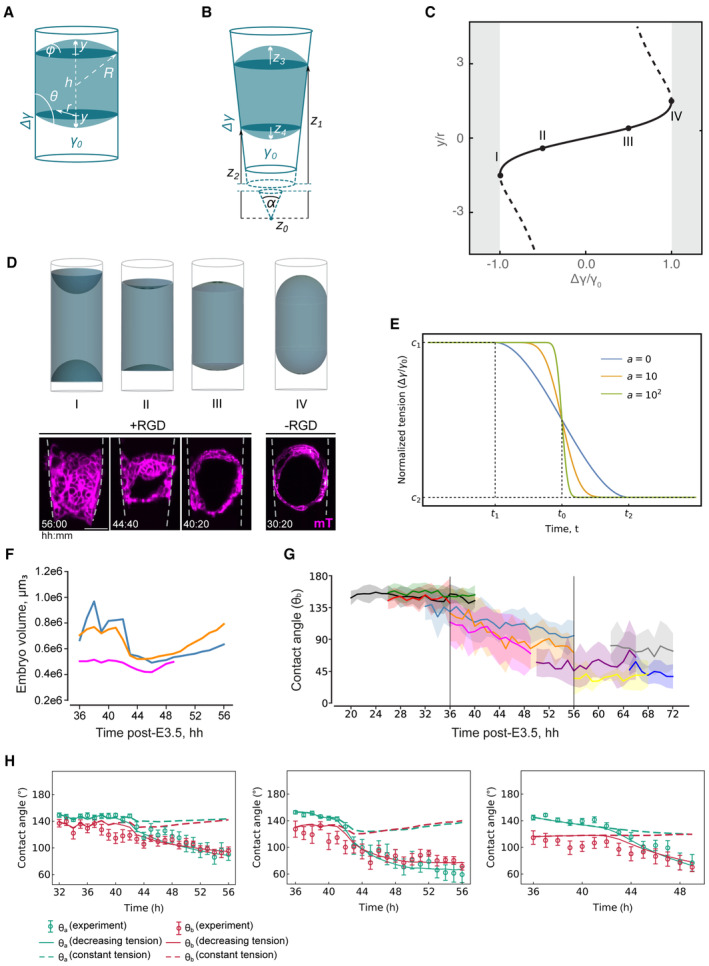
Characterization of the droplet‐wetting model The equilibrium shape of the droplet in a cylindrical confinement of radius *r* is described by the distance *h* between the two contact lines and by the height *y* of the top and bottom spherical caps, corresponding to polar and mural TE, respectively. These caps can also be characterized by the curvature radius *R* and angle *φ*. The contact angle *θ* depends on the droplet–medium tension *γ*_0_ and the Young tension Δγ.The droplet in a conical frustum with angle *α* is described by the positions of the top and bottom contact lines *z*_1_, *z*_2_ measured from the conical tip *z*_0_ = 0, and by the heights of the top and bottom spherical caps *z*_3_ and *z*_4_, respectively. When the caps curve into the embryo, their heights assume negative values.Bifurcation diagram for the equilibrium solutions Equation S6 (Appendix) of the droplet in cylindrical confinement. The solid line corresponds to the stable solution *y*_−_, whereas the dashed line denotes the unstable branch *y*_+_.Top, Calculated equilibrium shapes of the droplet in cylindrical confinement at the transition to total wetting (I), in the regime of partial wetting (II, III), and dewetting (IV). Bottom, Time‐lapse images of mTmG signal (magenta) in the embryos growing in 3E‐uterus with RGD, corresponding to the I‐III wetting regimes and without RGD, corresponding to dewetting (IV). T = 00:00, hours: minutes after recovery at E3.5. The crypt surface is outlined.Sigmoid model of the Young tension adaptation Equation S16 drawn for three values of the modulation parameter *a* ≥ 0. Constants *c*_1_ and *c*_2_ specify the initial and final values of the normalized tension. The adaptation begins at a time instance *t*_1_ and ends at a time instance *t*_2_. A full specification of the model requires five constants, for example, the mid‐time *t*_0_ = (*t*_1_ + *t*_2_)/2, the duration *Δt* = *t*_2_ − *t*_1_, the constants *c*_1_ and *c*_2_, and the modulation parameter *a*.Volume dynamics in the developing embryos between 36 and 56 h after E3.5. Colors correspond to different embryos; *n* = 3.Contact angle (θ_b_) dynamics in developing embryos. Colors correspond to different embryos imaged in time intervals between 20 and 72 h after E3.5; *n* = 10.From left to right, simulated contact angle dynamics for constant tension (dashed line), and decreasing tension (solid line), with experimental data points (green and red points for θ_a_ and θ_b_, respectively) for three different embryos between 36 and 56 h from recovery at E3.5. Error bars denote SEM. Scale bar, 50 μm.

This model predicts a relationship between the interfacial tensions, contact points, and contact angles between the droplet (embryo) and the substrate (uterus), given the volume of the droplet (Figs 4F and EV4A–D; Appendix).

We measured the volume from live imaging data of the embryo and calculated the contact angle dynamics in the presence and absence of adhesion changes between the droplet and the substrate using simulation‐based inference (Tejero‐Cantero *et al*, 2020) to determine the remaining parameter values of our model (Fig EV4E and F; Appendix Table S4). By including the experimentally measured volume changes, our model accounts for both the inflation‐collapse dynamics of the blastocoel (Chan *et al*, 2019) as well as for effects of cell proliferation.

Experimental measurement of the contact angle between the embryo and the 3E‐uterus surface showed a remarkable agreement with the theoretical values for increasing adhesion (Figs 4G and EV4G and H; Movie EV7). We confirmed the soundness of our model by performing leave‐one‐out cross‐validation and recovered a good agreement between predicted shape dynamics and experimental data in all cases (Appendix Fig S4). The quantitative and predictive agreement between our simple model and the experimentally measured contact angles in both embryonic (polar) and abembryonic (mural) parts of the embryo (Fig EV4H) shows that the predominant driving mechanism underlying embryo shape dynamics in 3E‐uterus is—in addition to embryo volume changes—an increase in adhesion between mTE cells and a substrate. More generally, this result suggests that the tissue‐scale shape dynamics resulting from embryo implantation can be biophysically understood as an active wetting process. This model further predicts that failure to adhere to the uterus should lead to maximum contact angles or near‐spherical embryo shapes, in line with the outcome of embryo culture in the hydrogel without RGD modification (Figs 3A and EV4D, IV right).

Notably, the postulated tension release at the embryo‐substrate interface exactly corresponds to the condition required for pTE cells to constrict apically, invaginate and form ExE—which had been achieved *ex vivo* only by removing the mTE and thereby releasing tension acting on the pTE (Bedzhov *et al*, 2014; Ichikawa *et al*, 2022). Light‐sheet microscopy and measurement of the pTE cell aspect ratio showed that pTE cells indeed undergo apical constriction (Fig 4I and J) within the predicted time interval (Fig 4H and J), indicating that the 3E‐uterus recapitulates the embryo‐uterus interaction, releasing the TE tension and enabling the development of the whole embryo *ex vivo* for the first time.

Collectively, these findings show that the embryo‐uterus tissue‐level interaction upon implantation can be biophysically described as a droplet‐wetting process and that this embryo‐uterus interaction releases tension acting on the TE, enabling ExE formation.

### Multiview light‐sheet microscopy reveals peri‐implantation egg cylinder growth dynamics

**Figure 5:**
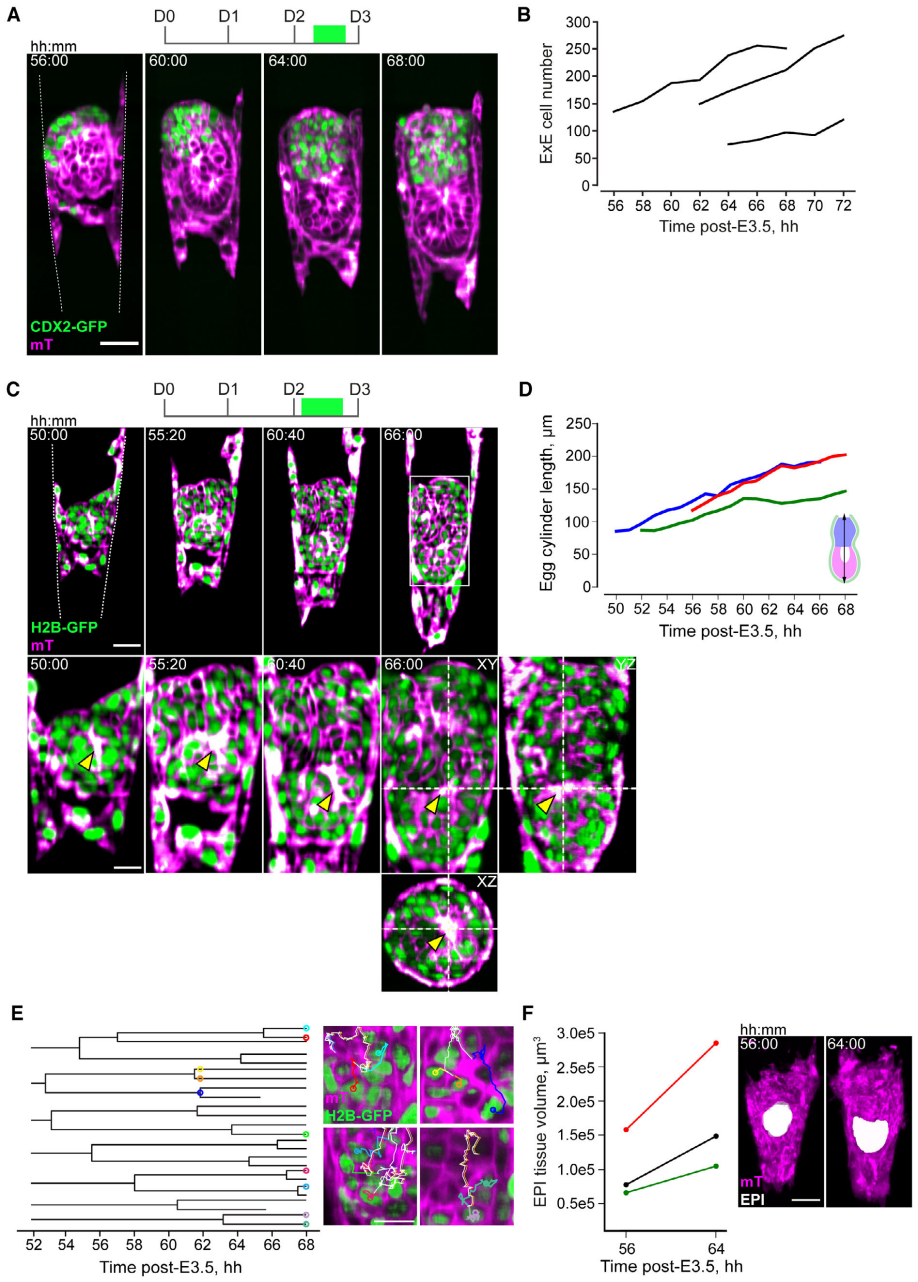
Multiview light‐sheet microscopy reveals peri‐implantation egg cylinder growth dynamics Time‐lapse images of the developing CDX2‐GFP (green); mTmG (magenta) embryo. t = 00:00, hours: minutes after recovery at E3.5.Quantification of the ExE cell numbers for three independent experiments.Time‐lapse images of the H2B‐GFP (green); mTmG (magenta) developing embryo. Bottom, 2x zoom into the epiblast region. Right and bottom, YZ and XZ image sections show 3D resolution. Yellow arrows indicate egg cylinder growth; asterisk, the pro‐amniotic cavity formation.Egg cylinder length between 50 and 68 h after recovery at E3.5. Colors correspond to independent experiments.Epiblast cell lineage dendrograms. Right, corresponding cells marked as dots with different colors overlaying the dendrograms and the image slices; cell lineage tracks are depicted as a 2D overlay.An increase in epiblast tissue volume between 56 and 64 h after recovery at E3.5. Colors correspond to independent experiments (same as D). Data information: Scale bars, 50 μm, 25 μm (2× zoom), 10 μm (E, right). Source data are available online for this figure.

To dissect the coordination between embryo growth, TB migration, and the uterus, we further characterized the growth dynamics of the tissues comprising the egg cylinder, using the MuVi‐SPIM. As a result of TE tension release as described above, CDX2‐GFP;mTmG embryos underwent ExE invagination and proliferation as well as egg cylinder patterning (Fig 5A and B; Movie EV8). Live imaging of H2B‐GFP;mTmG embryos showed tissue growth at cellular resolution without compromising egg cylinder proliferation and patterning (Fig 5C and D; Appendix Fig S5A and B; Christodoulou *et al*, 2018; Ichikawa *et al*, 2022). The egg cylinder elongated along the M/AM axis at a rate of 5.52 μm/h with its tip moving at 4.62 μm/h (Fig 5C and D; Movie EV9). EPI cell lineage tracks estimated an average cell cycle length of 8:38 hh:min (Fig 5E), and EPI tissue volume increased 1.78x (on average) over 8 hours (Fig 5F). Thus, our engineering approach of hydrogel microfabrication combined with multiview light‐sheet imaging revealed the cellular dynamics of embryonic and extraembryonic tissues and allowed us to quantitatively characterize their substantial growth and dynamic morphogenesis upon embryo‐uterus interaction.

### Spatial coordination of trophoblast dynamics and embryo growth delineates peri‐implantation development

**Figure 6:**
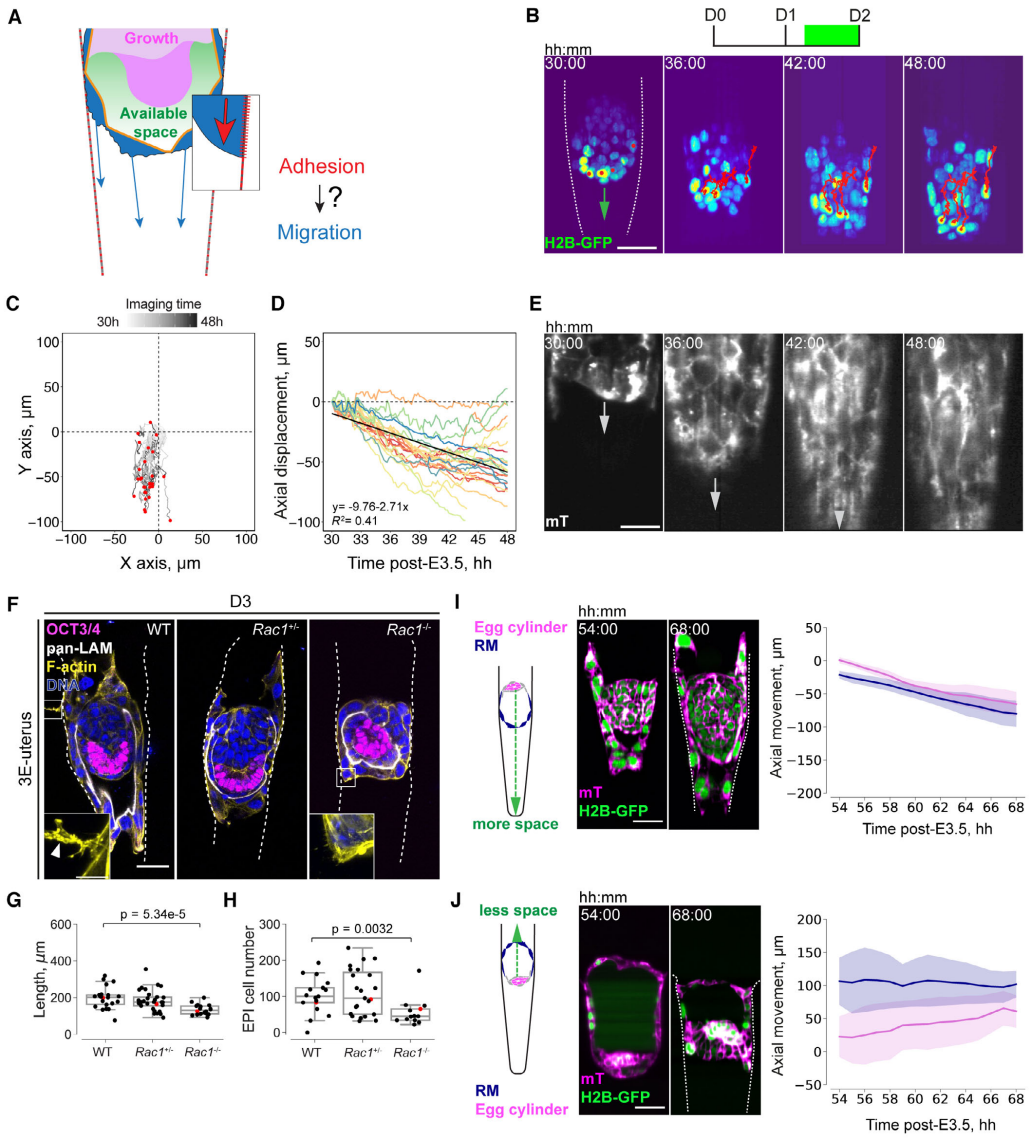
Spatial coordination of trophoblast migration and embryo growth delineates peri‐implantation development ASchematic of the hypothesis that adhesion‐induced migration of trophoblast (TB) cells generates space for embryo growth.B3D projections of time‐lapse images of the developing H2B‐GFP (green) embryo. Trajectories of individual mural TE (mTE) cells are marked with red lines. The crypt surface is outlined.CTrajectories of mTE cells in an XY plane, normalized to the starting coordinates. End coordinates are marked with red dots; *n* = 29.DDisplacement of mTE cells along the Y‐axis in relation to imaging time; *n* = 29. The linear regression fit is shown in black, y = −9.76‐2.71x, *R* ^2^ = 0.41.ETime‐lapse images of the developing mTmG (gray) embryo in the Z plane corresponding to the crypt surface. The arrow indicates direction of migration.FLeft to right, Immunostaining of WT, *Rac1* ^+/−^, and *Rac1* ^−/−^ embryos, cultured up to Day 3 (D3) in 3E‐uterus, showing OCT3/4 (magenta), pan‐Laminin (pan‐LAM, white), F‐actin (yellow), and nuclei (DNA, blue). The crypt surface is outlined, and the arrow points at the invasive trophoblast cell protrusion.G, HEmbryo length and epiblast cell number in WT, *Rac1* ^+/−^, and *Rac1* ^−/−^ embryos. *n* = 17 (WT), 23 (*Rac1* ^+/−^), 13 (*Rac1* ^−/−^), embryos pooled from five experimental replicates (epiblast cell number) and *n* = 22 (WT), 30 (*Rac1* ^+/−^), 18 (*Rac1* ^−/−^), embryos pooled from seven experimental replicates (embryo length). Data points correspond to embryos; the midline marks the median, the boxes indicate the interquartile range, and the whiskers extend maximum ± 1.5× interquartile range; the red dots mark representative embryos shown in (F). Mann–Whitney's U test *P*‐value.I, JEgg cylinder elongation and RM movement in a downward (I) and upward (J) embryo orientations. (I, J) left, Schematic of the egg cylinder tip (magenta) and the Reichert's Membrane (RM, blue) movement within an experimentally controlled space (green). Coordinates are scaled to the starting coordinate of the egg cylinder's tip. (I, J) middle, Time‐lapse images of H2B‐GFP (green); mTmG (magenta) developing embryos. (I, J) right, Movement of the egg cylinder tip (magenta) and RM (blue) along the crypt axis. Solid lines and shaded regions indicate average and SD values across two (downward) and four (upward) independent experiments. t = 00:00, hours: minutes from recovery at E3.5. Data information: Scale bars, 50 μm. Source data are available online for this figure.

**Figure EV5:**
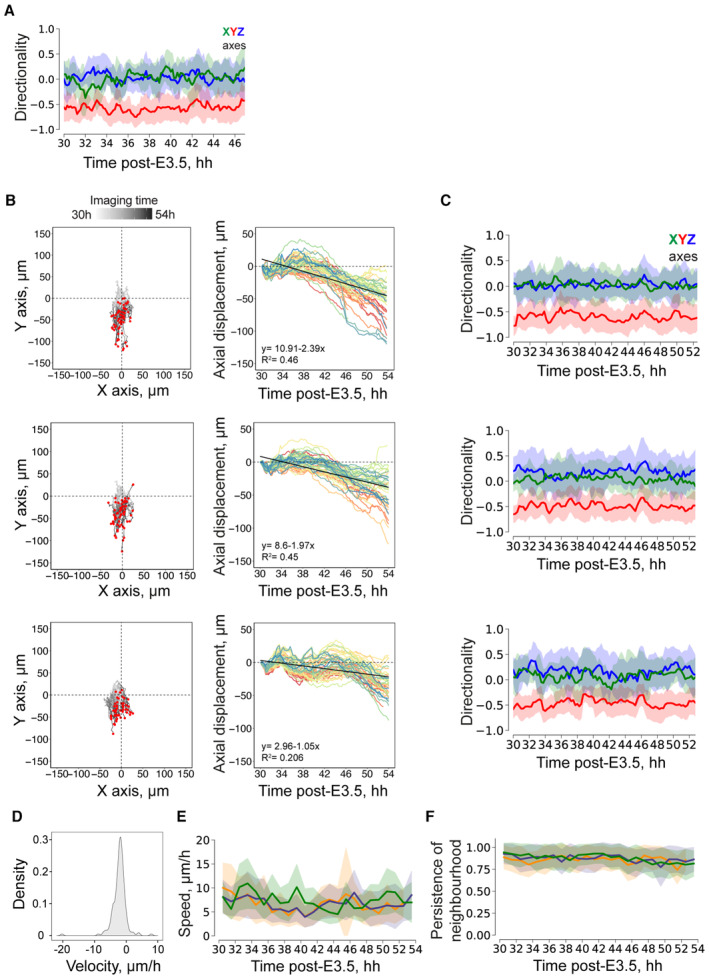
Characterization of collective trophoblast migration Directionality of the mTE/TB migration along the X, Y, and Z axes (green, red, and blue, respectively) between subsequent hours of live imaging. *n* = 29.Left, Mural TE (mTE) cell trajectories for three different embryos; coordinates in XY plane are normalized to the starting coordinates. End coordinates are marked with red dots. Right, Displacement of mTE cells along the Y‐axis vs imaging time post‐E3.5. From top to the bottom, *n* = 61, 58, 51, respectively. The linear regression fit is shown as a black line.Directionality of the mTE/TB migration along the X, Y, and Z axes (green, red, and blue, respectively) between subsequent hours of live imaging for three embryos (from top to bottom). *n* = 61, 58, 51, respectively.Distribution density of the average TB velocities (μm/h). *n* = 255, pooled from six embryos.TB migration speed (μm/h) vs imaging time post‐E3.5. Colors correspond to the three embryos from (B) and (C).Persistence of the nearest mTE/TB four‐cell neighborhood between subsequent hours of live imaging.

The observed egg cylinder morphogenesis and growth require space, which in the intact mouse embryo is delineated by the TB/RM boundary (Figs 1I and J, and EV1K). Removal of the TB and RM results in expansion of the epiblast tissue exceeding the *in utero* dimensions (Ichikawa *et al*, 2022). Based on our *in utero* and *ex vivo* evidence, we hypothesized that TB migration facilitates an extension of the TB/RM boundary along the uterine matrix (Fig 6A). Tracking mTE/TB cell nuclei labeled with H2B‐GFP (Hadjantonakis & Papaioannou, 2004) confirmed collective cell motility (Fig 6B; Movie EV10). Individual mTE/TB cells preferentially moved downward along the crypt axis (Figs 6C–E and EV5A–C) with an average velocity of 2.51 μm/h (Fig EV5D and E) and maintained the nearest neighbors (Fig EV5F), in agreement with their attachment to the RM. Nuclear divisions were rarely observed (12% of mTE/TB cell nuclei divided during 24 h of live imaging; *n* = 158), suggesting a limited contribution of mTE/TB cell division to their collective displacement.

**Figure 7:**
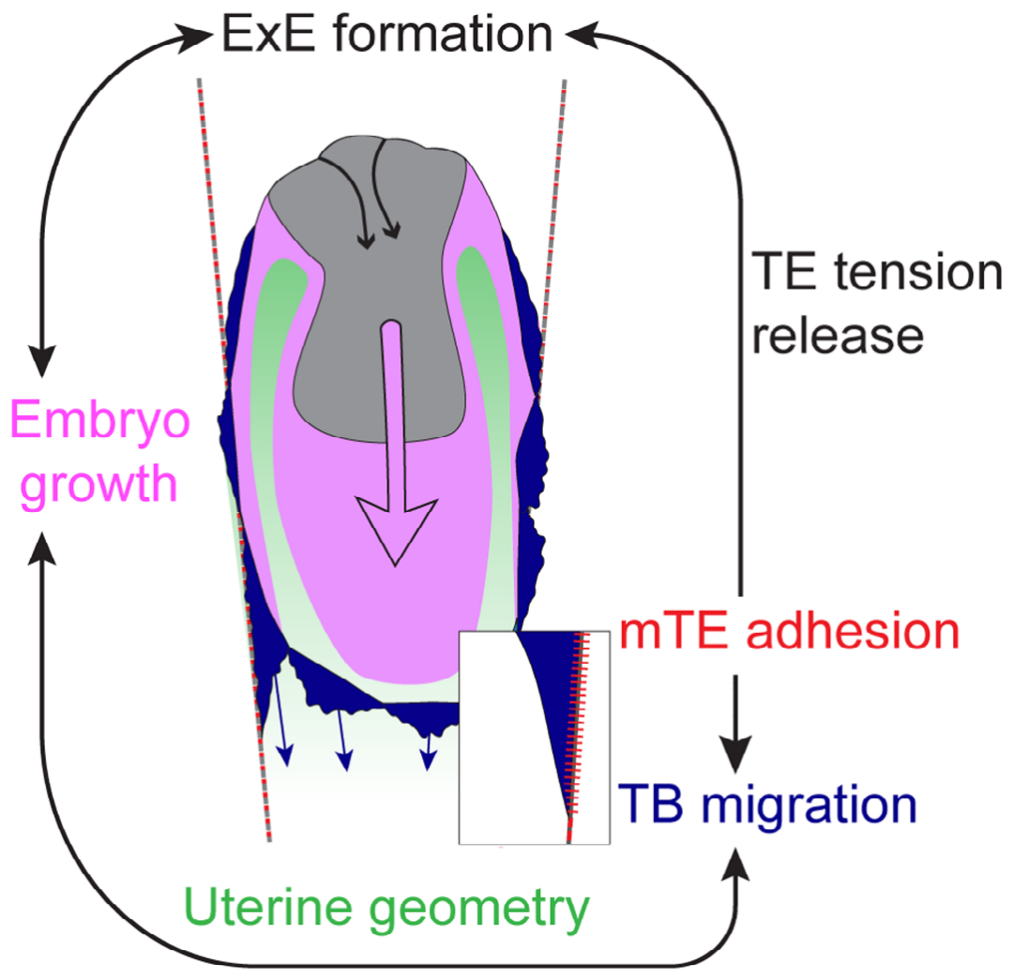
Embryo‐uterine coordination underlying mouse peri‐implantation embryogenesis The model for the coordination underlying mouse embryo development during implantation. Mural TE (mTE) adhesion to the uterine tissue triggers trophectoderm (TE) tension release, resulting in extraembryonic ectoderm (ExE) formation, and mTE/trophoblast (TB) motility which delineates uterine space for embryo growth and morphogenesis.

Hence, to address the coordination between egg cylinder morphogenesis, TB/RM boundary, and tissue geometry, we first perturbed TB motility. Genetic abrogation of Rac1^−/−^ reportedly shows growth retardation at E5.75 and arrest during gastrulation (Sugihara *et al*, 1998; Migeotte *et al*, 2010). 3E‐uterus culture revealed that elongation of the embryo is significantly limited in Rac1^−/−^ embryos (Fig 6F and G) with the compromised EPI growth (Fig 6F and H). Consistently, E5.25 Rac1^−/−^ embryo exhibited retarded growth *in utero* when compared to the WT embryo from the same litter (mTmG^+/−^Rac1^+/−^ male mated with Rac1^+/−^ female; Appendix Fig S6). Notably, Rac1 KO embryos also had defects in the parietal endoderm (Appendix Fig S6), revealing its essential role in parietal endoderm migration, too. Pharmacological inhibition of Rac1 by NSC23766 (Gao *et al*, 2004) further confirmed retention of the TB migration front in a reversible manner compared with the control embryos (Appendix Fig S7). Together, these results indicate that TB motility is dependent on Rac1 and is required for coordinated growth of embryonic tissues. Next, the displacement velocity of RM depends on the geometry of the 3E‐uterus, too. To interfere with it, we placed the embryo upside down (Fig 6J) close to the crypt opening, to limit the RM displacement without preventing individual TB cell displacement. Measurement of the displacement of the egg cylinder tip and RM front revealed that egg cylinder elongation is accompanied by a coordinated RM movement in 3E‐uterus (Fig 6I). However, with the embryo upside down, the displacement of RM relative to the egg cylinder growth is slower (Fig 6J), with fewer EPI cells, on average (Appendix Fig S8). The downward egg cylinder elongation was also blocked in shallow microwells, where space availability for embryo growth was limited (Appendix Fig S9). These data indicate that the spatial coordination is disrupted by a change in the 3E‐uterus geometry, leading to the impairment of the egg cylinder morphogenesis. Together, these experimental perturbations support the model in which the coordination between embryo growth, collective TB motility, and uterine geometry plays a key role in peri‐implantation mouse development (Fig 7).

## Discussion

In this study, we developed an engineered uterus, 3E‐uterus, which allowed us for the first time to culture the whole peri‐implantation mouse embryo *ex vivo*, recapitulating the embryo‐uterus interaction and supporting the differentiation of TB cells and Reichert's membrane. Combined with light‐sheet microscopy, this system allows for monitoring the cellular dynamics and perturbing cellular processes by genetic, pharmacological, and biophysical means, in order to gain insights into the underlying mechanisms. Our study revealed that integrin‐mediated adhesion of TB cells onto the uterine matrix not only releases TE tension to drive its invagination and ExE formation but also induces migration of TB cells. This TB cell migration, in turn, displaces Reichert's membrane so that the embryo has space for growth and morphogenesis. These findings thus reveal a dynamic coordination between embryo growth, TB cell migration, and uterine geometry that plays an essential role in peri‐implantation mouse development (Fig 7).

Our finding that the embryo adheres to the uterus for its morphogenesis is reminiscent of recent studies demonstrating a key role of attachment of the blastoderm or endoderm to the vitelline envelope in insect morphogenesis (Bailles *et al*, 2019; Münster *et al*, 2019). In the implanting mouse embryo, however, adhesion of the TB cells induces loss of polarity and collective migration.

To study the mechanics of embryo‐uterine interaction, we applied theoretical concepts from the physics of wetting. Theoretical approaches for classical wetting and active tissue wetting have yielded key insights into different multicellular spreading phenomena (Douezan *et al*, 2011; Alert & Casademunt, 2019; Pérez‐González *et al*, 2019; Gonzalez‐Rodriguez *et al*, 2021). Here, we modeled the embryo as a confined simple droplet with adaptive adhesion to the substrate and inferred the substrate‐adhesion dynamics from the observed shape changes (Figs 4C–J and EV4; Appendix). Our results suggest that embryo implantation occurs through a biologically tuned capillarity‐like process (Figs 4F and G, and EV4E–H; Movie EV7). The integrin‐mediated adhesion of TB cells to the uterine matrix not only releases TE tension to drive its invagination and ExE formation (Fig 4I and J) but also results in the collective motion of TB cells (Figs 6B–E and EV5). We show that TB displaces RM, so the embryo has space for growth and morphogenesis (Fig 6I and J). Thus, embryogenesis during implantation requires the coordination between tissues in both space and time. The space for embryo growth is defined by the TB and RM extension within a uterine tissue geometry, while the timing of TB‐matrix interaction matches pTE invagination and generation of ExE, which, in turn, influences embryonic growth.

On the basis of our findings in engineered environments, we propose that implantation into the uterine wall *in vivo* might be understood as a soft wetting problem (Andreotti & Snoeijer, 2020), that is, where a droplet interacts with and deforms a soft substrate. Such a capillarity‐based embedding mechanism would permit the self‐organization of embryo and uterus geometry. Testing whether the embryo utilizes this principle will become possible with the capacity to quantitatively measure implantation dynamics *in utero*, together with theoretical advances on complex wetting phenomena.

The geometric confinement can also facilitate the establishment of a murine cup‐shaped egg cylinder. As previous studies reported, the *ex vivo* confinement triggers A‐P symmetry breaking in the isolated egg cylinders (Hiramatsu *et al*, 2013; Matsuo & Hiramatsu, 2017). Here, we used an engineering approach to identify and address the key aspects provided by the context of the uterine matrix, TB, and RM at the onset of implantation. The revealed dynamics of embryonic and extraembryonic tissues is unique and necessary for understanding coordination in the triad of embryonic—extraembryonic—maternal tissues during implantation. The interaction between the embryo and the uterus likely involves dynamic changes in the uterine tissue (Kelleher *et al*, 2018; Flores *et al*, 2020; Ueda *et al*, 2020), studying which requires complementary development of methods for *in utero* monitoring (Huang *et al*, 2020). The engineering of the uterus‐like environment can also be developed further (Brassard & Lutolf, 2019; Chrisnandy *et al*, 2022; Qazi *et al*, 2022) to build up the complexity and dynamics of embryo‐uterine interactions and accommodate embryo development to more advanced stages. Incorporating endometrial, stromal cells (Boretto *et al*, 2017; Turco *et al*, 2017), and microfluidics (Nikolaev *et al*, 2020) into the engineering platform may improve the *ex vivo* culture and our understanding of the mechanisms of embryo‐uterine interactions. These new methods will open an exciting perspective for studying the feto‐maternal interaction upon implantation in other mammalian species and its co‐adaptation in evolution.

## Materials and Methods

### Reagents and Tools table

**Table:** 

Reagent or resource	Source	Identifier
Antibodies		
Mouse anti‐OCT3/4	Santa Cruz Biotechnology	Cat#sc‐5279; RRID:AB_628051
Goat anti‐GATA4	R&D systems	Cat#AF2606; RRID:AB_2232177
Rabbit anti‐SOX2	Cell Signaling Technology	Cat#23064; RRID:AB_2714146
Mouse anti‐CDX2	BioGenex	Cat#AM392; RRID:AB_2650531
Rabbit anti‐TFAP2C	Cell Signaling Technology	Cat#2320; RRID:AB_2202287
Rabbit anti‐Phospho‐Myosin Light Chain 2 (Thr18/Ser19)	Cell Signaling Technology	Cat#3674; RRID:AB_2147464
Rabbit anti‐Phospho‐Ezrin (Thr567)/Radixin (Thr564)/Moesin (Thr558)	Cell Signaling Technology	Cat#3726; RRID:AB_10560513
Mouse anti‐ZO‐1	Thermo Fisher Scientific	Cat#33‐9100; RRID:AB_2533147
Rabbit anti‐PARD6B	Santa Cruz Biotechnology	Cat#sc‐67393; RRID:AB_2267889
Rabbit anti‐Collagen IV	Millipore	Cat#AB756P; RRID:AB_2276457
Rabbit anti‐(pan) Laminin	Novus Biologicals	Cat#NB300‐144SS; RRID:AB_921870
Rabbit anti‐Fibronectin	Proteintech	Cat#15613‐1‐AP; RRID:AB_2105691
Rabbit anti‐IGFB1 (12G10)	Santa Cruz Biotechnology	Cat#sc‐59827; RRID:AB_782089
Rat anti‐ITGB1	Millipore	Cat#MAB1997; RRID:AB_2128202
Rabbit anti‐KRT8	DSHB	Cat#TROMA‐I; RRID:AB_531826
Donkey anti‐goat IgG Alexa Fluor 488	Thermo Fisher Scientific	Cat#A11055; RRID:AB_2534102
Donkey anti‐mouse IgG Alexa Fluor 488	Thermo Fisher Scientific	Cat#A21202; RRID:AB_141607
Donkey anti‐rat IgG Alexa Fluor 488	Thermo Fisher Scientific	Cat#A21208; RRID:AB_141709
Donkey anti‐rabbit IgG Alexa Fluor Plus 546	Thermo Fisher Scientific	Cat#A10040; RRID:AB_2534016
Donkey anti‐goat IgG Alexa Fluor Plus 555	Thermo Fisher Scientific	Cat#A‐21432; RRID:AB_2535853
Donkey anti‐mouse IgG Cy5 AffiniPure	Jackson ImmunoResearch	Cat#715‐175‐150; RRID:AB_2340819
Donkey anti‐rabbit IgG 647	Thermo Fisher Scientific	Cat#A‐31573; RRID:AB_2536183
Chemicals, peptides, and recombinant proteins		
8‐arm vinylsulfone‐functionalized PEG (PEG‐VS)	NOF	Custom synthesis
RGD peptide (Ac‐GRCGRGDSPG‐NH2)	Biomatik	Custom synthesis
Crosslinker peptide (Ac‐GCRE‐GPQGIWGQ‐ERCG‐NH2)	Biomatik	Custom synthesis
Triethanolamine (TEA)	Sigma	Cat#90278
DMEM, low glucose, pyruvate, no glutamine, no phenol red	Gibco	Cat#11880028
Fetal Bovine Serum	PAA	Cat#A15‐080
GlutaMAX	Gibco	Cat#35050061
HEPES	Merck	Cat#H0887
Penicillin‐Streptomycin	Gibco	Cat#15070063
Primocin	Invivogen	Cat#ant‐pm
Advanced DMEM/F‐12	Gibco	Cat#12634010
Global medium	CooperSurgical	Cat#LGGG‐050
Global w/ HEPES medium	CooperSurgical	Cat#LGGH‐050
Matrigel, Growth Factor Reduced	Corning	Cat#356230; lot: 7345012
Atelocollagen I	Reprocell	Cat#KKN‐IPC‐50
Fetal Bovine Serum, Embryonic stem cell–grade	Biosera	Cat#FB1001S
KnockOut Serum Replacement	Gibco	Cat#10828010
Insulin‐Transferrin‐Selenium‐Ethanolamine (ITS‐X)	Gibco	Cat#51500056
β‐estradiol	Merck	Cat#E8875
Progesterone	Merck	Cat#P0130
N‐acetyl‐L‐cysteine	Merck	Cat#A7250
NSC23766	Tocris	Cat#2161
Paraformaldehyde, EM Grade, Purified	Electron microscopy sciences	Cat#19208
SDS	Serva	Cat#20767
EDTA, pH 8.0	Invitrogen	Cat#15575020
Proteinase K	Merck	Cat#P2308
Tween‐20	Merck	Cat#P1379
Triton X‐100	Merck	Cat#T8787
Bovine serum albumin	Merck	Cat#A9647
DAPI (4',6‐Diamidino‐2‐Phenylindole, Dilactate)	Invitrogen	Cat#D3571
Rhodamine Phalloidin	Invitrogen	Cat#R415
Mineral Oil	Merck	Cat#M8410
Experimental models: Organisms/strains		
Mouse: (C57BL/6xC3H) F1	Laboratory Animal Resources at EMBL	N/A
Mouse: mTmG: Gt(ROSA)	The Jackson Laboratory; Muzumdar *et al* (2007)	Stock#007676; RRID: IMSR_JAX:007676
Mouse: H2B‐GFP: Tg(HIST1H2BB/EGFP)1Pa	The Jackson Laboratory; Hadjantonakis & Papaioannou (2004)	Stock#006069; RRID: IMSR_JAX:006069
Mouse: Myh9‐GFP: Myh9tm6(EGFP/MYH9)Rsad	Zhang *et al* (2012)	N/A
Mouse: Cdx2‐GFP: Cdx2tm1(EGFP)Yxz	The Jackson Laboratory; McDole & Zheng (2012)	Stock#018983; RRID:IMSR_JAX:018983
Mouse: Lifact‐GFP: Tg(CAG‐EGFP)Rows	The Jackson Laboratory; Riedl *et al* (2010)	MGI:4831036
Mouse: ZO1‐GFP: Tjp1tm(EGFP)Tlch	The Jackson Laboratory; Foote *et al* (2013)	MGI:5558017
Mouse: Rac1: Rac1tm1(flox)Ty	Walmsley *et al* (2003)	N/A
Oligonucleotides		
See Appendix Table S1 for Genotyping Primer List	N/A	N/A
Software and algorithms		
R v3.5.0	The R Foundation	https://www.r‐project.org/
		RRID:SCR_001905
RStudio v1.1.453	RStudio	https://rstudio.com/
		RRID:SCR_000432
ggplot2 v3.0.0	Hadley Wickham	https://ggplot2.tidyverse.org/
		RRID:SCR_014601
Python 3.8	Python Software Foundation	https://www.python.org/
		RRID:SCR_008394
SciPy	Virtanen *et al* (2020)	RRID:SCR_008058
PlantSeg	Wolny *et al* (2020)	https://github.com/hci‐unihd/plant‐seg
Wolfram Mathematica	Wolfram	https://www.wolfram.com/mathematica/
		RRID:SCR_014448
ZEN	Carl Zeiss	https://www.zeiss.com/microscopy/us/products/microscope‐software/zen.html
		RRID:SCR_013672
Imaris v9.2.1	Bitplane	https://imaris.oxinst.com
		RRID:SCR_007370
ICY	France Bioimaging	https://icy.bioimageanalysis.org
		RRID:SCR_010587
Fiji	Schindelin *et al* (2012)	https://fiji.sc
		RRID:SCR_002285
	Tischer *et al* (2021)	RRID: SCR_018484
Ilastik	Berg *et al* (2019)	RRID: SCR_015246
Paintera	HHMI Janelia	https://github.com/saalfeldlab/paintera
Luxendo Image Processor	Luxendo	https://luxendo.eu/
LuxControl	Luxendo	https://luxendo.eu/
Other		
μ‐Slide Angiogenesis Dish	Ibidi	Cat#81506
BD Eclipse Needle	BD	Cat#305757

### Methods and Protocols

#### Animal work

All animal work was performed in the Laboratory Animal Resources (LAR) at the European Molecular Biology Laboratory (EMBL) with permission from the Institutional Animal Care and Use Committee (IACUC) overseeing the operation (IACUC number TH11 00 11), and at the Animal Facility at the Hubrecht Institute. LAR facilities operated according to the Federation of European Laboratory Animal Science Associations (FELASA) guidelines and recommendations. At the Hubrecht animal facility, mice were housed according to institutional guidelines, and procedures were performed in compliance with the Standards for Care and Use of Laboratory Animals with approval from the Hubrecht Institute ethical review boards. Animal experiments were approved by the Animal Experimentation Committee (DEC) of the Royal Netherlands Academy of Arts and Sciences All mice were housed in IVC cages in pathogen‐free conditions with 12–12‐h light–dark cycle and used for experiments at the age of 8–35 weeks.

#### Mouse lines and genotyping

The following mouse lines were used in this study: a F1 hybrid strain between C57BL/6 and C3H (B6C3F1) as wild‐type (WT), Cdx2‐GFP (McDole & Zheng, 2012), mTmG (Muzumdar *et al*, 2007), H2B‐GFP (Hadjantonakis & Papaioannou, 2004), Lifeact‐GFP (Riedl *et al*, 2010), GFP‐Myh9 (Zhang *et al*, 2012), ZO1‐GFP (Foote *et al*, 2013). Rac1^flox/flox^ conditional allele (Walmsley *et al*, 2003) was crossed with ZP3‐Cre line (Lewandoski *et al*, 1997) to generate Rac1^+/−^ animals. To obtain zygotic Rac1^−/−^ embryos, Rac1^+/−^ females were crossed with Rac1^+/−^ males. Standard tail genotyping procedures were used to genotype transgenic mice (see Appendix Table S1 for primers and PCR product sizes).

#### Mouse embryos

Female estrous cycle synchronization was used to increase the natural mating efficiency (Whitten, 1957). The embryonic day 0.5 (E0.5) was defined as noon on the day when a vaginal plug was detected. Preimplantation mouse embryos were flushed from the uteri of the plugged females with 37°C KSOMaa with HEPES (Zenith Biotech, ZEHP‐050, 50 ml) using a syringe equipped with a cannula (Acufirm, 1400 LL 23). Embryos were handled using an aspirator tube (Sigma, A5177), connected to a glass pipette pulled from a glass microliter pipette (Blaubrand intraMark 708744). Procedures were performed under a stereomicroscope (Zeiss, StreREO Discovery.V8) equipped with a thermal plate (Tokai Hit) at 37°C (Behringer *et al*, 2014).

Peri‐implantation embryos were dissected from uteri in DMEM (Gibco, 11880028) supplemented with 15% heat‐inactivated FBS (PAA, A15‐080), 2 mM GlutaMAX (Gibco, 35050061), and 10 mM HEPES (Sigma, H0887), as described (Nagy *et al*, 2003).

#### LDTM hydrogel precursor synthesis

Low‐defect thiol‐Michael addition (LDTM) PEG hydrogel was synthesized and characterized according to the previously published study (Rezakhani *et al*, 2020). Briefly, to synthesize peptide‐functionalized PEG macromers (PEG‐PEP), vinyl sulfone‐functionalized 8‐arm PEG (8‐arm PEG‐VS) and the peptide Ac‐GCRE‐GPQGIWGQ‐ERCG‐NH2 (mol wt 1773.97 g/mol) with matrix metalloproteinases sensitive sequence (GPQGIWGQ) were dissolved in triethanolamine (TEA; 0.3 M, pH 8.0), and the 8‐arm PEG‐VS was added dropwise to the excess of peptides (VS/SH = 10) and reacted for 2 h at room temperature under inert atmosphere. The reaction solution was dialyzed (Snake Skin, molecular weight cutoff 10 K) against ultrapure water (pH < 7) for 5 days at 4°C, and the final product was lyophilized. The lyophilized product was dissolved in water to make 10% precursor solutions.

#### Hydrogel formation

LDTM hydrogels were formed by Michael‐type addition of PEG‐PEP precursors onto 8‐arm PEG‐VS. To make hydrogel networks of desired final PEG content, proper volumes of 10% (w/v) 8‐arm PEG‐VS in TEA and 10% (w/v) PEG‐PEP in water were mixed in molar stoichiometric ratio of VS/SH = 0.8. For example, to make 100 μl of LDTM hydrogels of 2.5% (w/v), taking into account each precursors' densities, 8.8 μl of 8‐arm PEG‐VS, 10 μl of TEA buffer, 65 μl of distilled water, and 16.20 μl of PEG‐PEP were mixed. For conditions containing RGD adhesion peptide (Ac‐GRCGRGDSPG‐NH2, mol wt 1002.04 g/mol), different volumes of RGD were added to the mix before addition of the PEG‐PEP precursor, and the molar ratio of VS/TH was adjusted as VS/(TH‐RGD) = 0.8. Appendix Table S2 shows the mixing values for LDTM gels (2.5% (v/w)) with different RGD contents.

#### Rheological measurements of hydrogels

The shear modulus (G′) of hydrogels was determined by performing small‐strain oscillatory shear measurements on a Bohlin CVO 120 rheometer with plate‐plate geometry. Briefly, 1–1.4 mm thick hydrogel disks were prepared and allowed to swell in water overnight. The mechanical response of the hydrogels sandwiched between the parallel plates of the rheometer was recorded by performing frequency sweep (0.1–10 Hz) measurements in a constant strain (0.05) mode at room temperature. Previously, we characterized stiffness and elasticity of the used hydrogel (Rezakhani *et al*, 2020).

#### Hydrogel holders and microtopography stamps fabrication

For hydrogel casting on the bottom of the 3.5 cm dish, special ring‐like PDMS holders were fabricated (see “Fabrication of topographically patterned hydrogels”). These hydrogel holders defined resulting hydrogel thickness to 1 mm and had overhanging features to hold the hydrogel block attached to the bottom of the plate. Holders were fabricated using conventional soft‐lithography methods established at the Center of Micronanotechnology (CMi, EPFL). In brief, the array of circular rings (∅ = 10 mm) was drawn using a CleWin (Phoenix Software). The designed layout was written with a diode laser onto a fused silica plate coated with chrome and positive photoresist (Nanofilm) using an automated system (VPG200, Heidelberg Instruments). Exposed photoresist was removed with a developer (DV10, Süss MicroTec), and the chrome layer underneath was etched with an acid–oxidizer solution of perchloric acid, cerium ammonium nitrate, and water. The resulting mask was developed with TechniStrip P1316 (Microchemicals) to remove the residual resist and extensively washed with ultrapure water. The mold was made from double‐layered epoxy‐based negative photoresist SU8. First, a 250 μm thick layer of SU8 GM1075 (Gerlteltec) photoresist was cast onto a dehydrated silicon wafer using a negative resist coater (LMS200, Sawatec). After baking at 110°C for 4 h, a second 250 μm thick layer of the SU8 GM1075 was coated, resulting in total thickness of a 500 μm. After second bake, this wafer was aligned and exposed to ultraviolet (UV) radiation through the mask (MA6/BA6, Süss MicroTec). After the postexposure bake at 85°C for 5 h, the wafer was developed with propylene glycol monomethyl ether acetate (Sigma) and baked at 135°C for 4 h. The wafer was then plasma‐activated and silanized with vapored trichloro (1H,1H,2H,2H‐perfluorooctyl) silane (Sigma‐Aldrich) overnight. This wafer was then used for polydimethylsiloxane (PDMS) molding (Sylgard 184, Dow Corning). Ten weight‐parts of elastomer base were vigorously mixed with one part of curing agent and poured onto the mold. After degassing under vacuum, PDMS was baked for 24 h at 80°C. The resulting PDMS replica was cut and punched with appropriate size biopsy punchers (5 mm for inner hydrogel area, 18 mm outside diameter). Resulting hydrogel holders were sterilized with UV and kept sterile until further use.

The stamps featuring micropillars topography were fabricated using conventional soft‐lithography methods established at the Center of Micronanotechnology (CMi, EPFL). In brief, the 3D models of the micropillars were designed in Autodesk Inventor. A diameter gradient was introduced into the crypt design to accommodate variability in blastocyst size (Fig 1C). The STL model was further processed in DeScribe 1.7 (Photonic Professional) to optimize it for printing 2PP lithography (NanoScribe GT2, Photonic Professional). The printing parameters were defined to have a slicing distance of 1 μm and a hatching distance of 600 nm. Writing was performed in the galvo scan mode and the 3D model was divided in 400 × 400 μm subblocks, corresponding to the FOV of 25× objective. The model was printed in acrylic photopolymer resin IP‐S (Photonic Professional), which was deposited on the surface of indium‐tin‐oxide (ITO)‐coated soda lime slides. Once the printing process was finalized, the slides were developed for 7 min in propylene glycol monomethyl ether acetate (PGMEA, Sigma‐Aldrich), followed by 5 min rinsing with ultrapure isopropanol and gentle blow‐drying. Then, samples were UV cured for 10 min (MA6/BA6, Süss MicroTec) and backed in the 80°C oven for 3 h. To fabricate a master mold featuring inverted topography, 3D printed models were then plasma‐activated and silanized with vapored trichloro (1H,1H,2H,2H‐perfluorooctyl) silane (Sigma‐Aldrich) overnight. Then, polydimethylsiloxane (PDMS, Sylgard 184, Dow Corning) was used for molding. Ten weight‐parts of elastomer base were vigorously mixed with one part of curing agent and poured onto the mold. After degassing under vacuum, PDMS was baked for 12 h at 80°C. The resulting PDMS replica was cut, plasma‐activated, and silanized with vapored trichloro (1H,1H,2H,2H‐perfluorooctyl) silane (Sigma‐Aldrich) overnight. This replica was used multiple times as a master for molding stamps, following the same protocol (PDMS Sylgard 184, 1:10 ratio, baked at 80°C for 12 h).

#### Fabrication of topographically patterned hydrogels

Elastomeric stamps containing the desired geometries in bas‐relief were coated with bovine serum albumin in PBS (1% w/v in PBS; Thermo Fisher Scientific) for overnight to prevent hydrogel adhesion. Before use, stamps were washed once with distilled water and dried with the gentle air blowing. For hydrogel casting, PDMS ring holders were placed on the bottom of the 3.5 cm dish and UV sterilized prior use. Then, a drop of liquid hydrogel precursor (see “Hydrogel formation”) was made in the center of the ring spacer and then a stamp with microtopography was placed atop. After 30–40‐min polymerization in the incubator (37 C), stamps were removed and hydrogels were covered with PBS. Hydrogels were used either the same day or stored for about 1 week.

#### Embryo culture

##### 3E‐uterus

*In Vitro* Culture medium (IVC1 and IVC2) was prepared as described (Bedzhov *et al*, 2014). Hydrogels with microfabricated crypts (see “Hydrogel formation” and “Fabrication of topographically patterned hydrogels”) were equilibrated in 3 ml of IVC1 medium in an incubator with a humidified atmosphere of 5% CO_2_ at 37°C (Thermo Scientific, Heracell 240i) for at least 12 h prior to 3E‐uterus embryo culture. After recovery at noon on embryonic day 3.5 (E3.5), embryos were serially transferred to IVC1 microdrops in a culture dish covered with mineral oil (Sigma, M8410‐1L). The time of embryo culture was counted from the time of embryo recovery (D0 = E3.5). In approximately 1 h after recovery, embryos were briefly treated in Tyrode's solution (Sigma, T1788) to remove *Zona pellucida*, washed repeatedly (Behringer *et al*, 2014), and left in a culture dish with IVC1 medium for at least an hour inside the incubator. Zona‐free embryos were carefully positioned inside microfabricated hydrogel crypts in a downward mTE orientation with a fused tip of a thin glass pipette. Precise positioning of the embryo within a crypt is a critical step significantly effecting the efficiency. The medium was exchanged to IVC1 in 24 h (Day 1) and to IVC2 in 48 h (Day 2).

##### 3D hydrogel‐embedded culture

LDTM PEG hydrogel components were mixed on ice. Fifteen microlitre of the mix was added to an inner well of a prewarmed μ‐Slide Angiogenesis dish, and embryos were carefully transferred and mixed inside the hydrogel (2–3 embryos per drop). To prevent embryos from adhering to glass or reaching gel surface, the dish was flipped regularly during gel solidification inside the incubator. Thirty‐five microlitre of prewarmed IVC1 medium was then added to each well. Subsequently, the medium was exchanged, and then exchanged again to IVC1 in 24 h (Day 1) and to IVC2 in 48 h (Day 2).

#### Single embryo genotyping

Individual embryos were mouth pipetted into 200 μl PCR tubes containing 10 μl of lysis solution of 200 μg/ml Proteinase K in Taq polymerase buffer (Thermo Fischer Scientific, B38). The lysis reaction was carried out for 1 h at 55°C, followed by 10 min at 96°C. The resulting genomic DNA was mixed with relevant primers (Appendix Table S1) for determination of genotype via PCR.

#### Single‐cell RNA sequencing

E4.5 and E5.25 embryos, dissected from the uterus, and Day 2 (D2) and Day 3 (D3) embryos, grown in 3E‐uterus, were dissociated in TrypLE solution for up to 12 min. After the first 6 min in TrypLE, embryos were gently pipetted up and down with a glass pipette to facilitate dissociation. Glass pipette diameter was chosen to first mechanically decouple trophectoderm and the Reichert's membrane, followed by a narrower pipette to dissociate the remaining tissues. The generated cell suspension was transferred into M2 media drops under the oil to prevent media evaporation at room temperature. Using a glass pipette and an aspirator, single cells were then manually distributed into 384‐well plates containing barcoded poly‐T primers under oil. The plates were centrifuged and stored at −80°C until library preparation.

Plate‐based VASA‐seq was performed according to (Salmen *et al*, 2022) with double volumes. Cells were lysed, and RNA was heat fragmented. Fragmented RNA was end‐repaired using T4‐PNK and A‐tailed by *E. coli* Poly(A) Polymerase. Repaired RNA was reverse transcribed with SuperScriptIII (SSIII), and second‐strand synthesis was carried out in the plate. After pooling, cDNA was amplified by *in vitro* transcription (IVT). Amplified RNA was depleted for ribosomal RNA using mouse‐specific DNA probes in combination with RNAseH. RA3 was ligated onto the 3′ end of the depleted aRNA, reverse transcription was performed using SSIII, and the final PCR amplification was performed to introduce small RNA PCR primer indexes. Plates were sequenced paired‐end on the Illumina NextSeq2000 2 × 50 bp, with 25 bp for read 1 and 75 bp for read 2.

Raw fastq files were processed according to the VASA‐seq Snakemake workflow of the SingleCellMultiOmics (SCMO) package (version 0.1.30; SCMO pipeline https://github.com/BuysDB/SingleCellMultiOmics). In brief, reads were demultiplexed for VASA barcodes with a hamming distance of 0 and trimmed for default adapters with CutAdapt (version 4.1; Martin, 2011), and the remaining polyA stretches were trimmed off with the SCMO script trim_vasa.py. Reads #2 were mapped to the mouse GRCm38 genome (Ensembl 97) using STAR (version 2.5.3a; Dobin *et al*, 2013). Mapped reads were tagged, filtered for a mapping quality > 50, and deduplicated using samtools (version 1.15.1; Li *et al*, 2009). Transcript counts of deduplicated files were generated using velocyto (version 0.17.17; La Manno *et al*, 2018).

Downstream analysis was performed with Scanpy (version 1.9.1; Wolf *et al*, 2018). Cells with less than 1,500 reads and 50 detected genes were removed as well as genes detected in less than two cells. Cells with more than 40% mitochondrial reads were additionally excluded. Protein‐coding genes were used for further analysis (excluding mitochondrial genes, Malat1, SnoRNAs, and ribosomal proteins). Counts were normalized to 10.000 transcripts per cell (scanpy.pp.normalize_per_cell) and logarithmized. (scanpy.pp.log1p). The number of total counts and percentage of mitochondrial reads were regressed out (scanpy.pp.regress_out), and each gene was scaled to unit variance with a maximum of 10 (scanpy.pp.scale). Principal component analysis was performed, and the 30 highest principal components were used to generate the UMAP. The data were clustered using the Leiden algorithm (scanpy.tl.leiden, resolution set to 0.3).

Cell cycle state was determined by scoring the cell cycle genes (scanpy.tl.score_genes_cell_cycle) determined as in Tirosh *et al* (2016). The correlation matrix was produced by extracting the first 50 PCs and calculating the Pearson correlation using pandas (version 1.4.2) “corr” function. Statistical tests were performed using add_stat_annotation from statannot (version 0.2.3), using Mann–Whitney tests with Bonferroni multiple testing correction.

#### Immunofluorescence preparation and staining

Recovered embryos were fixed with 4% paraformaldehyde (Electron microscopy sciences, 19208) in PBS for 15 min at room temperature. For *ex vivo* cultured embryos, the hydrogel with embryos was gently dissected and fixed with 4% paraformaldehyde for 30 min at room temperature with agitation. For immunostaining of active integrin and di‐phosphorylated myosin regulatory light chain (ppMRLC), fixation was performed in 1% PFA in PBS supplemented with MgCl_2_. The samples were subsequently washed in PBST buffer (0.1% Tween‐20 in PBS; Sigma, 85113), and *ex vivo* cultured embryos were carefully dissected from the hydrogel at this step. Permeabilization was performed with 0.5% Triton X‐100 (Sigma, T8787) in PBS for 30 min at room temperature with gentle agitation. After several washes in the wash buffer (2.5% BSA (Sigma, A9647) in PBST), embryos were incubated in the blocking buffer (5% BSA in PBST) overnight at 4°C. Embryos were stained with primary antibodies diluted in the blocking buffer overnight at 4°C. After washes, embryos were incubated with secondary antibodies diluted in the wash buffer for 2 h at room temperature with gentle agitation. Staining with rhodamine phalloidin (Invitrogen, R415) diluted at 1:500 was performed together with secondary antibodies. Subsequently, embryos were washed in PBST with DAPI (Invitrogen, D3571) at 5 μg/ml and mounted in PBST.

Primary antibodies against GATA4 biotinylated (R&D systems, AF2606), SOX2 (Cell Signaling, 23064), TFAP2C (Cell Signaling, #2320), CDX2 (Biogenex Laboratories, MU392AUC), PARD6B (Santa Cruz Biotechnology, sc‐67393), pan‐Laminin (Novus Biologicals, NB300‐144SS), Collagen IV (Millipore, AB756P), Fibronectin (Proteintech, 15613‐1‐AP), ITGB1 (Millipore, MAB1997), and GFP (chromotek, gb2AF488) were diluted at 1:200. Primary antibodies against active ITGB1 (12G10) (Santa Cruz Biotechnology, sc‐59827), ZO1 (Invitrogen, 33‐9100), di‐phosphorylated myosin regulatory light chain (ppMRLC) (Cell Signaling, 3674), and phosphorylated ERM (pERM) (Cell Signaling, 3726) were diluted at 1:100. Primary antibodies against OCT3/4 (Santa Cruz Biotechnology, sc‐5279) and KRT8 (Troma‐1‐C, AB531826) were diluted at 1:50.

The following secondary antibodies were used at 1:400: donkey anti‐goat IgG Alexa Fluor 488 (ThermoFisher, A11055), donkey anti‐rat IgG Alexa Fluor 488 (ThermoFisher, A21208), donkey anti‐mouse IgG Alexa Fluor 488 (ThermoFisher, A21202), donkey anti‐rabbit IgG Alexa Fluor Plus 546 (Invitrogen, A10040), donkey anti‐goat IgG Alexa Fluor Plus 555 (Invitrogen, A21432), donkey anti‐mouse IgG Cy5 AffiniPure (Jackson ImmunoResearch, 715‐175‐150), and donkey anti‐rabbit IgG 647 (ThermoFisher, A31573).

#### Cryosectioning

Pregnant mouse uteri were dissected and handled in KSOM with HEPES. To reduce nonphysiological uterine contraction due to the release from connecting tissues, uteri were transferred to prewarmed 0.5 M MgCl_2_ solution. Uteri were cut into pieces corresponding to the embryo implantation sites, as visually judged by their swollen and opaque appearance under the stereomicroscope. Tissue pieces were immediately fixed in 4% PFA in PBS overnight at 4°C, followed by an overnight wash in PBS at 4°C, and subsequent overnight washes in 12% Sucrose, 15% Sucrose, and 18% Sucrose at 4°C until further use within 2 weeks. The tissue pieces were dried with KIMTECH paper (Kimberly‐Clark) and mixed with M‐1 Embedding Matrix for cryosectioning (ThermoScientific, 1310TS). Tissue pieces were mounted and orientated in M‐1 Embedding Matrix in Tissue‐Tek cryomold (Sakura) and frozen at −80°C.

Cryosectioning was performed with Leica CM3050S cryotome at −16°C, to produce sections of 15–20 μm thickness using low‐profile microtome blades (Accu‐Edge, Sakura). Tissue sections were dried at room temperature, washed in PBST, and permeabilized for 15 min using 0.5% Triton X‐100 in PBS. Immunostaining was performed as described above.

#### Confocal imaging

Confocal imaging was performed on Zeiss LSM 780 Confocal Inverted Microscope with LD C‐Apochromat 40×/1.1 W Corr objective, using Zen 2012 LSM Black software and LSM880 Airyscan Confocal Inverted Microscope with a C‐Apochromat 40×/1.2 NA water immersion objective, using Zen 2.3 SP1 Black software v14.0.0.0. Nuclear immunostaining of OCT3/4, GATA4, and TFAP2C was imaged by LSM780 or LSM880 confocal mode (evaluation of 3E‐uterus) with 1 μm Z spacing. Immunostainings of embryos and tissue sections were also imaged with Airyscan Optimal or Superresolution modes with optimal Z spacing, calculated based on the used imaging settings. The following lasers were used: diode 405 nm, argon multiline 458/488/514 nm, and HeNe 561 nm and 633 nm. Raw Airyscan images were processed by ZEN 2.3 SP1 Black software v14.0.0.0 or v14.0.12.201. See Appendix Table S3 for the summary of the microscopy types used throughout the study.

#### Light‐sheet live imaging with Muvi‐SPIM

##### Custom sample holder assembly and embryo mounting

To support long‐term embryo viability, we implemented atmospheric and temperature regulation of the MuVi‐SPIM imaging chamber. Moreover, we developed a new engineering approach to precisely position the embryo within the hydrogel microenvironment. Our design prevented the embryo from exchanging liquid with the rest of the imaging chamber, providing sterility and efficient usage of the culture medium.

The sample holder encompasses two transparent and gas‐permeable FEP tubes. The outer tube (∅_inner_ = 1.7 mm, ∅_outer_ = 1.8 mm) contains the medium and is supported by a PDMS‐filled capillary from the bottom and sealed by a PDMS cap from the top. The inner tube (∅_inner_ = 1.05 mm, ∅_outer_ = 1.15 mm) is supported by the tube holder made of PDMS. Molds for the tube holder and the cap were made from Teflon using custom microfabrication. For PDMS preparation, elastomer and a curing agent (Sylgard 184, Dow Corning) were mixed at a 10:1 ratio (w/w). After degassing in a vacuum chamber, the molds were baked in the oven at 60°C overnight.

A single‐embryo cavity was cast inside the PEG hydrogel precursor‐filled inner FEP tube using a custom single‐embryo‐shaped PDMS stamp (see “Hydrogel holders and microtopography stamps fabrication”). IVC medium was exchanged several times inside the outer tube to equilibrate the hydrogel prior to embryo mounting. An embryo was carefully mounted with a glass pipette from the opening of the outer tube, closed with a cap, and immediately placed in the incubator. IVC medium was exchanged twice per day.

##### Microscope and imaging settings

Multiview light‐sheet microscope is equipped with 2 Olympus 2 mm WD 20x/1.0 NA water immersion objectives (XLUMPLFLN20XW) used for detection, and 2 Nikon 3.5 mm WD 10x/0.3 NA water dipping objectives (CFI Plan Fluor 10X W) used for illumination. The detection path further consists of a filter wheel, a Nikon TI‐E 1× tube lens (Nikon Instruments Inc.), and a CMOS camera (ORCA‐Flash4.0 V2, Hamamatsu Photonics K.K.), The captured 3D data have a voxel size of 0.295 × 0.295 × 1.000 μm^3^ along the X, Y, and Z axes, respectively. The recorded volume size amounts to 302.08 × 604.15 × 150–250 μm^3^. The following lasers and filters were used: 488 nm (LuxX® series, Omicron‐Laserage Laserprodukte GmbH) and BP525/50 (525/50 BrightLine HC, Semrock, IDEX Health & Science LLC), 561 nm (OBIS LS 561, Coherent Inc.) and LP561 (561 LP Edge Basic Langpass‐Filter, Semrock, IDEX Health & Science LLC). Dual light‐sheet illumination was used, paired with line‐scan detection mode (de Medeiros *et al*, 2015) with a slit width of 40 px. The exposure was set to 30 ms. Live imaging was performed under 5% CO_2_ and 19.5% O_2_ atmospheric conditions at 37°C inside the controlled environmental imaging chamber.

##### MuVi‐SPIM image processing

The volumes acquired with the left and right cameras were fused using the Luxendo Image Processor (v2.4.1., Luxendo, Bruker Corp). For further quantification and analysis, the image drift was corrected in Fiji (Schindelin *et al*, 2012) with the BigDataProcessor2 plug‐in (Tischer *et al*, 2021).

#### Light‐sheet live imaging with InVi‐SPIM

An array of micro‐cavities was fabricated inside the PEG hydrogel‐filled TruLive3D Dishes using custom PDMS stamp, containing a single row of micro‐cavities (see “Hydrogel holders and microtopography stamps fabrication”). The dish bottom was covered with 35 μl of the PEG hydrogel precursor mix; the PDMS stamp was carefully placed parallel to the side of the detection objective. After hydrogel solidification for 30–40 min in the incubator, 200–300 μl of PBS was added atop and the stamp was pulled out with forceps. Several washes with IVC1 medium were performed before embryo culture. Embryos were carefully mounted into crypts in a downward mTE orientation, and IVC1 medium was added up to 115 μl and covered with 250 μl mineral oil to prevent evaporation during live imaging. IVC medium was exchanged as described (see “Embryo culture”). Live imaging was performed under 5% CO_2_ and 19.5% O_2_ atmospheric conditions at 37°C inside the controlled environmental imaging chamber. The InVi‐SPIM is equipped with a Nikon 25x/1.1NA water dipping objective (CFI75 Apochromat 25XC W, Nikon Instruments Inc.) used for detection, Nikon 3.5 mm WD 10x/0.3 NA water dipping objectives (CFI Plan Fluor 10X W) used for illumination, and CMOS camera (ORCA‐Flash4.0 V2, Hamamatsu Photonics K.K.). Voxel size: 0.104 × 0.104 × 1.000 μm^3^ along the X, Y and Z axes, respectively. The following lasers and filters were used: 488 nm and BP525/50 (525/50 BrightLine HC, Semrock, IDEX Health & Science LLC), 561 nm and LP561 (561 LP Edge Basic Langpass‐Filter, Semrock, IDEX Health & Science LLC) Exposure time was set to 50 ms. Imaging was performed with line‐scan mode in LuxControl (Luxendo, Bruker Corp).

#### Pharmacological Rac1 inhibition and live imaging

Embryos were recovered at E3.5 (D0) and manipulated according to the 3E‐uterus protocol. Embryos from the same litter were split into two isolated TruLive3D dish compartments for the parallel live imaging of the treatment and the control conditions. Live imaging started at 30 h counted from the time of embryo recovery. A single mTomato channel was illuminated with a 561 nm laser every 20 min during subsequent live imaging intervals. At 36 h, IVC1 medium in one compartment was exchanged to IVC1 medium supplemented with 100 μM NSC23766 (treatment) and in another compartment to IVC1 medium supplemented with an equal amount of H_2_O (control). The supplemented medium (for both treatment and control) was exchanged 3–4 times with several‐minute incubation time intervals to equilibrate the concentrations. Imaging restarted at 37 h until 48 h. Between 48 and 49 h, the medium was exchanged in the same way to nonsupplemented IVC2 for both the treatment and the control conditions. Live imaging restarted at 49 and continued until 72 h, after which embryos were fixed and immunostained. Image voxel size: 0.208 × 0.208 × 1.000 μm^3^ along the X, Y, and Z axes, respectively.

#### Image analysis software

Dimension measurements and cell counting were performed with Imaris v9.2.1 (Bitplane). ICY (de Chaumont *et al*, 2012) was used for cell tracking. Fiji (Schindelin *et al*, 2012; Berg *et al*, 2019) was used for kymograph analysis, basal membrane segmentation, contact angle quantification, volume measurements, and fluorescence intensity quantification for plasma membrane proteins. Ilastik (Berg *et al*, 2019) was used for CDX2‐GFP nuclear signal segmentation. Paintera software was used to generate and correct the ground truth segmentation (https://github.com/saalfeldlab/paintera).

#### Nuclei segmentation

The 3D data volumes were acquired with either LSM780 or LSM880 in a confocal mode with a voxel size of 0.207 × 0.207 × 1 μm^3^ or 0.23.23 × 0.23 × 1 μm^3^, for X, Y, and Z dimensions, respectively. The channels corresponding to anti‐OCT3/4, anti‐GATA4, and anti‐CDX2 immunostainings were used. A 3d UNet (preprint: Çiçek *et al*, 2016) was trained with a multitask objective: predicting the binary nuclei mask in the first output channel and predicting the nuclei boundaries/outlines in the second output channel. The boundary predictions were then used to recover the individual nuclei using PlantSeg's “MutexWS” partitioning algorithm. The nuclei foreground prediction is used in postprocessing for removing spurious instances in the background.

Model training was performed iteratively with an increasing amount of ground truth data. Starting from four initial ground truth data volumes, in each iteration, we trained the network, performed the segmentation, and manually proofread the results in order to increase the training set and accuracy. In total, 22 training and 13 validation data volumes were used for the final model training. The size of the training volumes ranged from [117, 703, 377] to [162, 1052, 1840] voxels in Z, X, and Y dimensions.

#### Membrane‐based cell segmentation

The data volumes were acquired with MuVi‐SPIM (see “Light‐sheet live‐imaging with Muvi‐SPIM”). A dedicated 3D UNet was trained to predict the foreground membrane mask, which was used for the final cell segmentation with PlantSeg's “GASP” agglomeration algorithm. The ground truth for the network training was bootstrapped by initially segmenting the stacks with pretrained PlantSeg models (“confocal_unet_bce_dice_ds2x”), followed by manual correction of the erroneous cells. In total, four annotated stacks were used for training and one for validating the network. Both nuclei and membrane UNets were trained using Adam optimizer (preprint: Kingma & Ba, 2014) with β1 = 0.9, β2 = 0.999, L2 penalty of 0.00001, and initial learning rate ε = 0.0002. Networks were trained until convergence for 100 K iterations, using the PyTorch framework (preprint: Paszke *et al*, 2019). The models with the best score on the validation set were selected.

#### Embryo staging by cell numbers

Cell counts for E4.5‐E6.0 *in utero* embryos were obtained from the previous study (Ichikawa *et al*, 2022). Cells for E3.5 *in utero* embryos were manually counted based on GATA4 and SOX2 immunostaining. Linear regression analysis for embryo staging was performed as described (Ichikawa *et al*, 2022). For successfully developed 3E‐uterus embryos, epiblast (EPI) cells were defined based on the nuclear OCT3/4 expression. Cells with nuclear GATA4 expression overlying epiblast cells were defined as visceral endoderm (VE). OCT3/4 and GATA4 channels were used for automatic EPI and VE nuclei segmentation (see “Machine‐learning‐based nuclei segmentation”). For the absolute quantification accuracy, manual correction and cell counting were performed on top of the automated nuclei segmentation.

#### Evaluation of 3E‐uterus efficiency

Efficiency was quantified as a percentage of successfully developed embryos among all embryos at Day 3 of 3E‐uterus. 3E‐uterus embryo was classified as successfully developed if three criteria were met (Fig EV1E).

- Egg cylinder formation, defined as EPI tissue located within a VE layer with the basal membrane in between.
- Alignment of the egg cylinder axis with the crypt axis. The embryos with an evident upward egg cylinder orientation were excluded from quantifications due to an experimental error of embryo positioning (corresponding to less than 5% of samples).
- Formation of the Reichert's membrane, determined as a basal membrane underneath TB which, at the top of the egg cylinder, was required to continue into the basal membrane between EPI and VE.

To directly assess the criteria i–iii, the simultaneous immunostaining against OCT3/4, GATA4, Collagen IV, or pan‐Laminin, and nuclei (DAPI) was performed each time. For evaluation of 3E‐uterus efficiency, three independent experiments were performed, among which 46% of embryos (12 of 26) met all the above mentioned criteria.

Efficiencies for crypt diameter evaluation (Fig EV1F) were calculated as follows:

80 μm crypt diameter: 0/4, 0/5, and 1/8 (the number of successfully developed embryos divided by the total number of embryos); three independent experiments.
100 μm: 0/3, 2/9, and 2/8; three independent experiments.
120 μm: 1/3, 1/5, and 2/5; three independent experiments.
140 μm: 3/8, 2/5, 1/3, 2/7, and 1/4; five independent experiments.
160 μm: 2/4, 0/5, and 1/5; two independent experiments.

Efficiencies for PEG content evaluation (Fig EV1G) were calculated as follows:

1.5% PEG content: 2/6 and 4/8 (the number of successfully developed embryos divided by the total number of embryos); two independent experiments.
1.7%: 1/6 and 2/8; two independent experiments.
2%: 3/9 and 1/5; two independent experiments.
3%: 0/4, a single experiment.

Efficiencies for the hydrogel stiffness evaluation (Fig EV1H and I) were calculated as follows:

1.5% PEG‐PEP content: 3/8, 3/8, and 2/8 (the number of successfully developed embryos divided by the total number of embryos); three independent experiments.
1.75%: 3/9, 5/8, 2/7, and 4/8; four independent experiments.
2%: 2/7, 3/6, and 4/6; three independent experiments.
2.25%: 0/6, 6/13, and 1/5; three independent experiments.
2.5%: 1/6, 2/7, and 4/17; three independent experiments.
2.75%: 1/9, 5/15, and 1/9; three independent experiments.
6%: 1/6 and 0/7; two independent experiments.
7%: 1/11 and 0/5; two independent experiments.

#### Sample size estimation and blinding

We did not apply statistical tests to determine the sample size. The investigators were not blinded in this study.

#### Extraembryonic ectoderm cell number counting

Nuclei were counted based on CDX2‐GFP signal in MuVi‐SPIM 3D data volumes using automated Spots detection with manual correction in Imaris.

#### Trophoblast cell tracking

Individual cells on the mural TE side of the H2B‐GFP expressing embryos were tracked in 3D over 18–24 h of imaging, starting from 30‐h post‐E3.5 recovery.

#### Cell speed quantification

The mTE/TB cell speed was quantified as the Euclidian distance between the mTE/TB nuclei positions in the adjacent hours of live imaging using a sliding time window with a size corresponding to 1 h and a step size of an image time resolution (10 or 15 min; Fig EV5E).

#### Cell directionality quantification

The sliding window (see “Cell speed quantification”) was used to define mTE/TB vector between time points. We calculated the angle (α) between mTE/TB vector and the unit vectors corresponding to X, Y, and Z axes. “Directionality” was calculated as (180 ‐ α)/90–1, ranging from −1 to 1 values (Fig EV5A and C).

#### Quantification of the neighborhood persistence

The cell neighborhood was defined for each TB cell as the nearest four TB cells. Persistence of neighborhood was quantified as a proportion of cell neighbors maintained between adjacent hours of live imaging, ranging from 0 to 1.

#### Fluorescence intensity quantification for plasma membrane proteins

Identical imaging settings were applied for the samples in Fig 3E–H to enable comparison. The fluorescence signal of ZO1, PARD6B, and phosphor‐Ezrin/Radixin/Moesin was measured in Fiji using a line tool, 5 pixels in width, drawn along the cell's perimeter. Signal intensity values along the cell perimeter were exported for analysis and visualization in R. The signal was normalized to the average nuclear DAPI signal within the same Z plane.

#### Quantification of the contact angle at the mural TE‐hydrogel interface

Image volumes were manually transformed with BigDataProcessor2 Fiji plug‐in for vertical crypt alignment along the y‐axis. Images were XZ‐resliced followed by 180^o^ radial reslice about the center of the line of symmetry. Microwell surface was identified based on the background hydrogel fluorescence. Fiji's Ange tool with a handle length of 15–20 μm was used to quantify the angle (θ) between the crypt surface and the cell membrane on the mural and polar TE sides. θ values were quantified on the left and right sides of the image every 30^o^. The final θ value represents the averaged value across the crypt circumference.

#### Kymograph analysis

To quantify mural TE and EPI displacements, a kymograph was drawn parallel to the crypt axis and the edge of the membrane signal was tracked. Per each embryo, the values were averaged across three lines per Z‐slice in three different Z locations.

#### Polar TE cell shape analysis

Polar TE cell length and the width were manually measured with Imaris based on the overlay of the cell membrane segmentation output and the raw signal. The dimensions were measured for 15–20 polar TE cells per embryo every hour of live imaging.

#### Embryo length analysis

Embryo length was quantified in 3D as a distance between the outermost giant trophoblast nucleus and the outermost nucleus of the polar TE/ExE along the crypt axis (Fig 6G).

#### Basal membrane segmentation

Segmentation of the basal membrane (BM) between EPI and PrE from the Reichert's membrane was performed with the segmentation editor (https://imagej.net/plugins/segmentation‐editor) in Fiji based on anti‐Collagen IV or anti‐pan‐Laminin immunostaining data. 2D Roi with the BM data signal was converted into continuous contours using a custom Python script.

#### Middle axis estimation and length computation

The binary 3D segmentation of the basal membrane (BM) between EPI and PrE was used for analysis. First, the Euclidean distance transform (DT) was applied to the 3D segmentation to construct a directed graph in which the nodes are the nonzero valued pixels of the DT. The edges of the graph were assigned with weights that represent the difference between the global maximum of the DT values and the DT value of the target node. The shortest path in the weighted graph was then computed between two nodes that correspond to manually annotated points on the specimen's surface that mark its extreme poles (Dijkstra, 1959). The nodes on the shortest path were used to fit an open cubic B‐spline (Schoenberg, 1969) curve that approximates the middle axis. Finally, the integration over the spline was performed in order to obtain the arc length of the egg cylinder.

#### Diameter estimation

Similarly, the binary 3D segmentation of the BM was used for diameter estimation. Two landmark points, which correspond to the middle of the EPI tissue density, were manually annotated on the specimen's surface. The landmark points were then used to determine a plane that intersects the specimen orthogonally with respect to the estimated middle axis (see the previous section). More specifically, the plane was fitted such that it minimizes the Euclidean distance to the landmark points under the constraint of being orthogonal to the middle axis. The closed circular curve, resulting from the intersection of the specimen and the plane, was then used to compute the diameter.

#### Materials availability

All unique/stable reagents generated in this study are available from the Lead Contacts with a completed Materials Transfer Agreement.

## Author contributions

**Vladyslav Bondarenko:** Conceptualization; resources; data curation; formal analysis; funding acquisition; validation; investigation; visualization; methodology; writing – original draft; writing – review and editing. **Mikhail Nikolaev:** Conceptualization; resources; methodology; writing – original draft; writing – review and editing. **Dimitri Kromm:** Conceptualization; resources; formal analysis; visualization; methodology; writing – review and editing. **Roman Belousov:** Formal analysis; validation; inference and simulations; visualization; methodology; writing – original draft; writing – review and editing. **Adrian Wolny:** Resources; data curation; software; formal analysis; methodology; writing – original draft. **Marloes Blotenburg:** Data curation; formal analysis; visualization; writing – review and editing. **Peter Zeller:** Data curation; formal analysis; writing – review and editing. **Saba Rezakhani:** Resources; methodology; writing – review and editing. **Johannes Hugger:** Resources; methodology. **Virginie Uhlmann:** Supervision. **Lars Hufnagel:** Supervision; funding acquisition. **Anna Kreshuk:** Resources; software; supervision; methodology. **Jan Ellenberg:** Supervision. **Alexander van Oudenaarden:** Supervision; funding acquisition. **Anna Erzberger:** Conceptualization; formal analysis; supervision; funding acquisition; investigation; visualization; methodology; writing – original draft; writing – review and editing. **Matthias P Lutolf:** Conceptualization; supervision; funding acquisition; investigation; writing – original draft; writing – review and editing. **Takashi Hiiragi:** Conceptualization; supervision; funding acquisition; investigation; writing – original draft; writing – review and editing.

## Disclosure and competing interests statement

The authors filed a patent EP23162464.4 devoted to the live imaging and embryo culture method.

## Supporting information



Appendix S1

Expanded View Figures PDF

Movie EV1

Movie EV2

Movie EV3

Movie EV4

Movie EV5

Movie EV6

Movie EV7

Movie EV8

Movie EV9

Movie EV10

PDF+

Source Data for Figure 1

Source Data for Figure 2

Source Data for Figure 3

Source Data for Figure 4

Source Data for Figure 5

Source Data for Figure 6

## Data Availability

The trained nuclei and cell segmentation models were deposited at https://bioimage.io. All ground truth datasets can be downloaded from https://doi.org/10.5281/zenodo.6546550. The code to reproduce the image segmentation can be found in the GitHub repository https://github.com/kreshuklab/mouse‐embryo‐seg. The raw single‐cell RNA sequencing data were deposited at GEO data repository under GSE228264 (http://www.ncbi.nlm.nih.gov/geo/query/acc.cgi?acc=GSE228264).
