# Supplementary material for: Embryo‐uterine interaction coordinates mouse embryogenesis during implantation
Source: EMBO J. 2023 Jul 31;42(17):e113280. doi: 10.15252/embj.2022113280 (PMC10476174; doi:10.15252/embj.2022113280)
Supplement: Supplementary file 1 — Appendix S1 [file EMBJ-42-e113280-s008.pdf]

Appendix

EMBRYO-UTERINE INTERACTION COORDINATES MOUSE EMBRYOGENESIS DURING IMPLANTATION

Bondarenko V. [1,2,10]*, Nikolaev M.* [3,11]*, Kromm D.* [4,12]*, Belousov R.* [4]*, Wolny A.*[4]*, Blotenburg M.*[5]*, Zeller P.*[5]*, Rezakhani S.*[3,13]*, Hugger J.*[4,6]*, Uhlmann V.*[6]*, Hufnagel L.*[4,14]*, Kreshuk A.*[4]*, Ellenberg J.*[4]*, van Oudenaarden A.*[5]*, Erzberger A.*[4,7,*]*, Lutolf M.*[3,11,**]*, Hiiragi T.*[1,5,8,9,***]

[1]European Molecular Biology Laboratory, Developmental Biology Unit, 69117 Heidelberg, Germany
[2]Faculty of Biosciences, University of Heidelberg, 69120 Heidelberg, Germany
[3]Institute of Bioengineering, Ecole Polytechnique Fédérale de Lausanne (EPFL), Building AA-B 039, CH-1015 Lausanne, Switzerland
[4]European Molecular Biology Laboratory, Cell Biology and Biophysics Unit, 69117 Heidelberg, Germany
[5]Hubrecht Institute, Uppsalalaan 8, 3584 CT Utrecht, Netherlands
[6]EMBL-EBI, Wellcome Genome Campus, CB10 1SD Hinxton, UK
[7]Department of Physics and Astronomy, Heidelberg University, 69120 Heidelberg, Germany
[8]Institute for the Advanced Study of Human Biology (WPI-ASHBi), Kyoto University, 606-8501 Kyoto, Japan
[9]Department of Developmental Biology, Graduate School of Medicine, Kyoto University, Kyoto, Japan

[10]Present address: Weizmann Institute of Science, Herzl St. 234, 7610001 Rehovot, Israel
[11]Present address: Institute of Human Biology (IHB), Roche Pharma Research and Early Development, Basel, Switzerland
[12]Present address: Delft Center for Systems and Control, Delft University of Technology, Delft, Netherlands
[13]Present address: Novartis Institutes for BioMedical Research, Novartis Pharma AG, 4056 Basel, Switzerland
[14]Present address: Veraxa Biotech, Heidelberg, Germany

[*]Correspondence: erzberge@embl.de
[**]Correspondence: matthias.lutolf@epfl.ch
[***]Correspondence: t.hiiragi@hubrecht.eu

TABLE OF CONTENTS

APPENDIX FIGURE S1 3

APPENDIX FIGURE S2 4

APPENDIX FIGURE S3 5

APPENDIX FIGURE S4 6

APPENDIX FIGURE S5 7

APPENDIX FIGURE S6 8

APPENDIX FIGURE S7 9

APPENDIX FIGURE S8 10

APPENDIX FIGURE S9 11

APPENDIX TABLE S1 12

APPENDIX TABLE S2 13

APPENDIX TABLE S3 14

APPENDIX TABLE S4 15

APPENDIX NOTE ON THE THEORETICAL MODEL 16

Appendix Figure S1

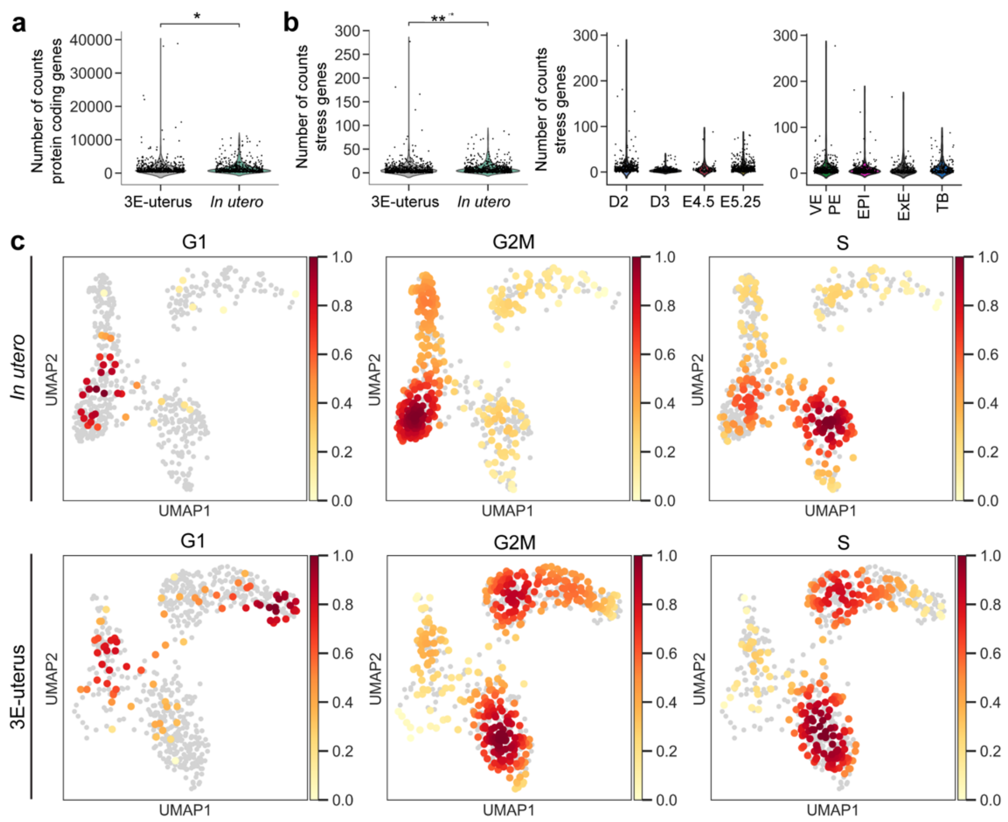

Appendix Figure S1. Quality control and characterization of the single-cell transcriptome data. a, Distributions of the numbers of raw counts mapped to protein coding genes in the cells from 3E-uterus and from *in utero* embryos. P-values, *: 1.00e-02 < p <= 5.00e-02, **: 1.00e-03 < p <= 1.00e-02, Mann-Whitney test with Bonferroni multiple testing correction. **b***,* Total gene expression counts of stress genes across different stages and cell types. The plots show overall low expression of the stress marker genes (Junb, Fosb, Fos, Hspa1a, Hspa1b, Jun, Hspa8, Hsp90ab1, Hspb1, Egr1, Hsp90aa1, Zfp36, Cebpd, Jund, Hspe1, Atf3, Socs3) in our data (van den Brink SC et al., 2017). **c**, From left to right, phase densities of the cell cycle stages (G1, G2M, S) projected on the UMAP, and determined as in (Tirosh et al., 2016); top, *in utero*; bottom, 3E-uterus.

Appendix Figure S2

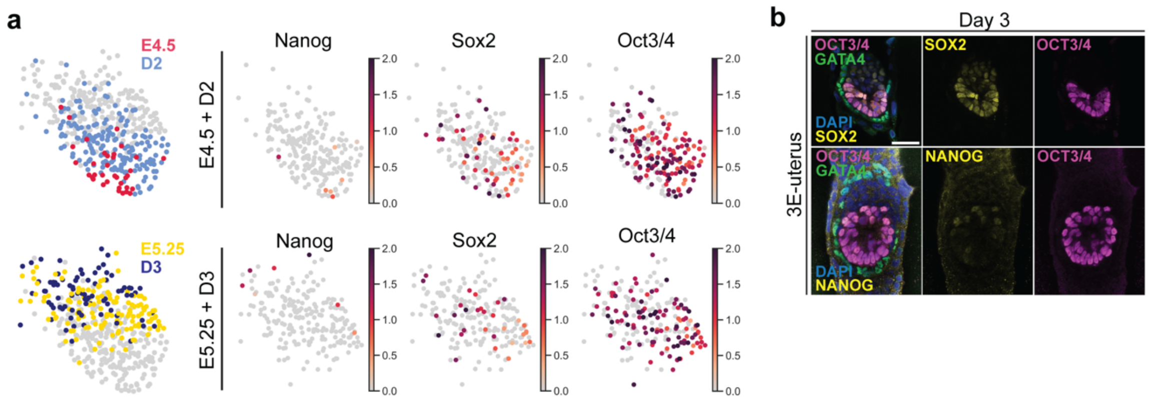

Appendix Figure S2. The characterization of pluripotency in epiblast. a, Left, the UMAPs coloured by the experimental condition: E4.5 (red) and D2 (light blue), as well as E5.25 (yellow) and D3 (dark blue) across the EPI cluster (total n = 398). **a**, Right, the UMAPs coloured by the normalized gene expression of Nanog, Sox2, and Oct3/4 among *in utero* (top) and 3E-uterus (bottom) EPI cells**. b,** Immunostaining of 3E-uterus Day 3 embryos showing heterogeneous SOX2 signal (yellow, top), low/no NANOG (yellow, bottom), OCT3/4 (magenta), GATA4 (green), and nuclei (DAPI, blue). Scale bar, 50 μm.

Appendix Figure S3

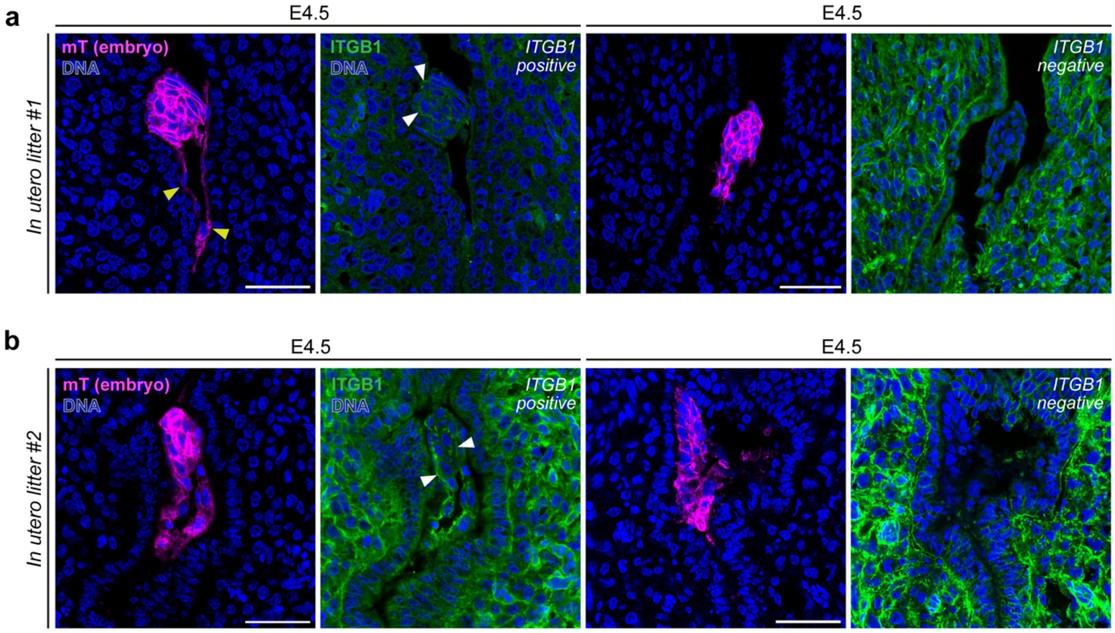

Appendix Figure S3. Characterization of Itgb1 KO *in utero*. a, Immunostaining of the two E4.5 pregnant uteri cross-sections from the same Itgb1$^{+/-}$ female mouse mated with a double-transgenic mTmG (hom) and Itgb1$^{+/-}$ male, showing the embryo (mT, magenta), Integrin beta 1 (ITGB1, green), and DNA (DAPI, blue). The embryo on the left has distinct ITGB1 signal in the cell-cell interfaces (marked with white arrows) and the inner lining of the blastocoel whereas the embryo on the right has no specific and detectable signal. The embryo-uterine attachment and protrusions are marked with yellow arrows. **b**, Same immunostaining as in **(a)**, but with the embryos from a different female mouse mated as above. Scale bars, 50 μm.

Appendix Figure S4

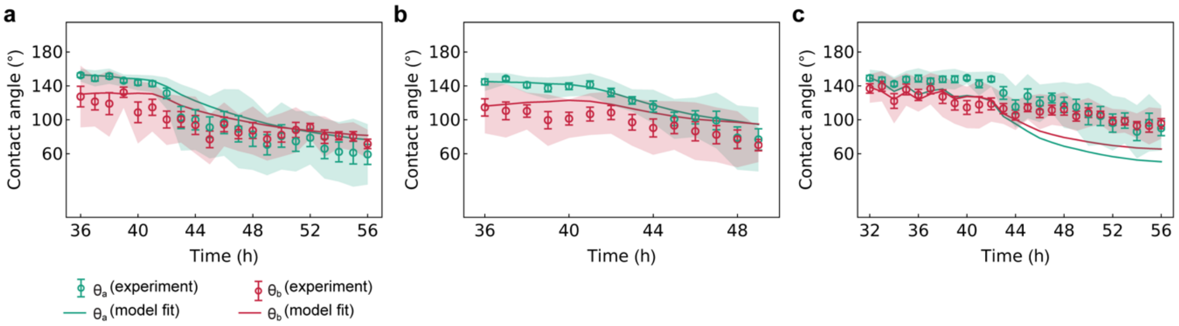

Appendix Figure S4. Leave-one-out validation of the droplet-wetting model (Sec. 2.2 in Appendix). Within three standard deviations of the mean, given by the bands, our model predicts relaxation of the contact angle in two embryos (panels a–b) The onset of the wetting in third embryo agrees with the prediction of the model (panel c), but the contact angle decreases more rapidly after 44h in experiments. Error bars denote one s.e.m.

Appendix Figure S5

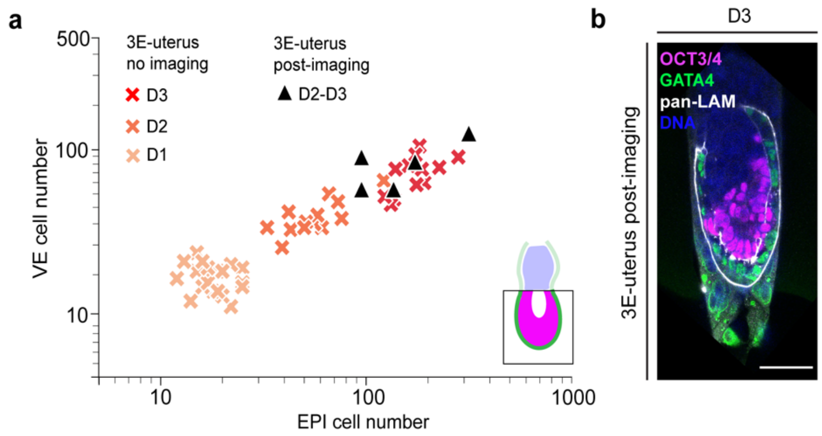

Appendix Figure S5. Evaluation of embryo morphology after live imaging. a, The numbers of epiblast (EPI) cells (x-axis) vs the numbers of visceral endoderm (VE) cells (y-axis) that cover EPI (the bottom right scheme) in 3E-uterus embryos developed in the incubator for three days (D1-D3, no imaging), and 3E-uterus embryos developed in the incubator, and then live imaged with MuVi-SPIM for 20 – 24 hours up to day 3 (D3, after imaging). N = 12, pooled from three experimental replicates (D3, no imaging), n = 5, pooled from five experimental replicates (D3, after imaging). The groups of imaged and not imaged D3 embryos did not significantly differ in terms of EPI (P = 0.69) and VE (P=0.37) cell numbers. Student's t-test *P*-values. XY scale, log 10. **b**, immunofluorescence of the day 3 embryo after live imaging with MuVi-SPIM showing OCT3/4 (magenta), GATA4 (green), pan-Laminin (pan-LAM, white), and nuclei (DNA, blue). Scale bar, 50 μm.

Appendix Figure S6

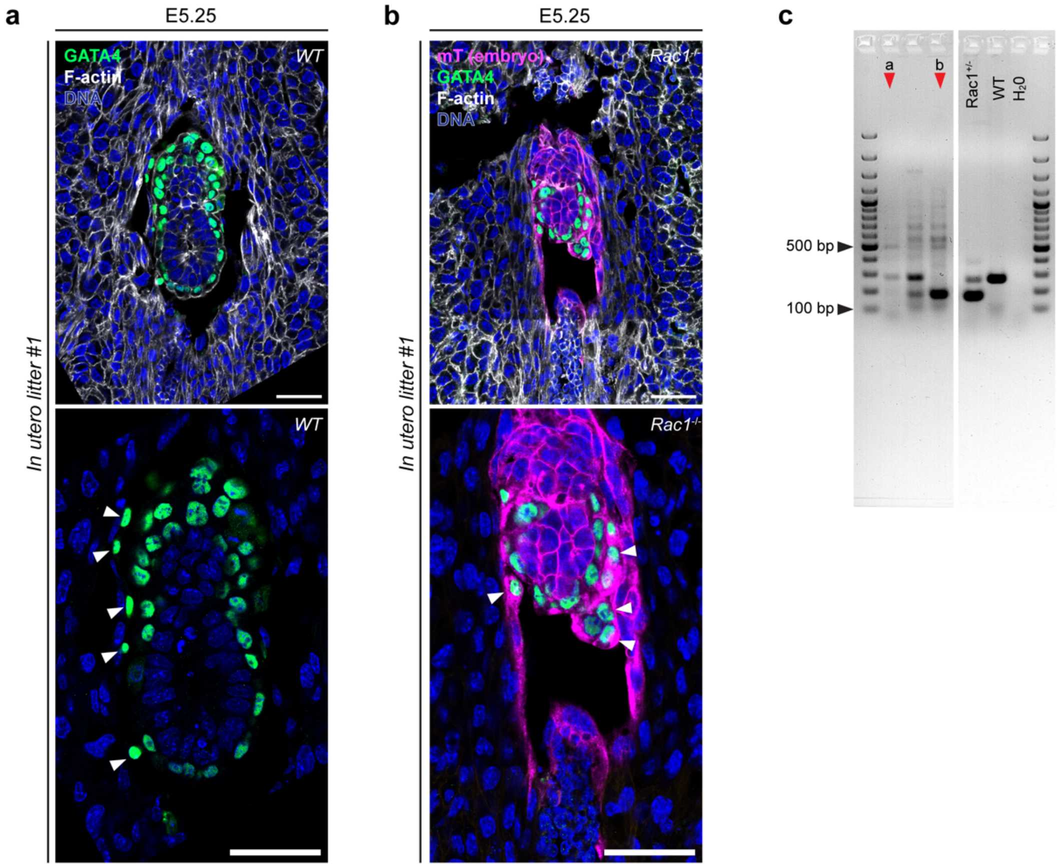

Appendix Figure S6. Examination of embryonic Rac1 KO *in utero* at E5.25. a, b Immunostaining of the two E5.25 pregnant uteri cross-sections from the same Rac1$^{+/-}$ female mouse mated with a double-transgenic mTmG (het) and Rac1$^{+/-}$ male, showing the embryo (mT, magenta), GATA4 (green), F-actin (white), and DNA (DAPI, blue). Bottom, 2x zoom. The embryo in **(a)** was developed normally whereas the embryo in **(b)** had abnormal egg cylinder size, lack of parietal endoderm cells (marked with white arrows) on the embryo perimeter, and accumulation of GATA4-positive cells on one side of the egg cylinder. **c**, Agarose gel showing PCR results of embryo genotyping with Rac1 primers (see Appendix Table S1). Red arrows point to the samples of the embryos in **(a)** and **(b)**. After imaging, egg cylinders were carefully dissected from the corresponding tissue sections (20 µm thick) and used as a source of DNA for genotyping. Scale bars, 50 µm, 25 µm (2x zoom).

Appendix Figure S7

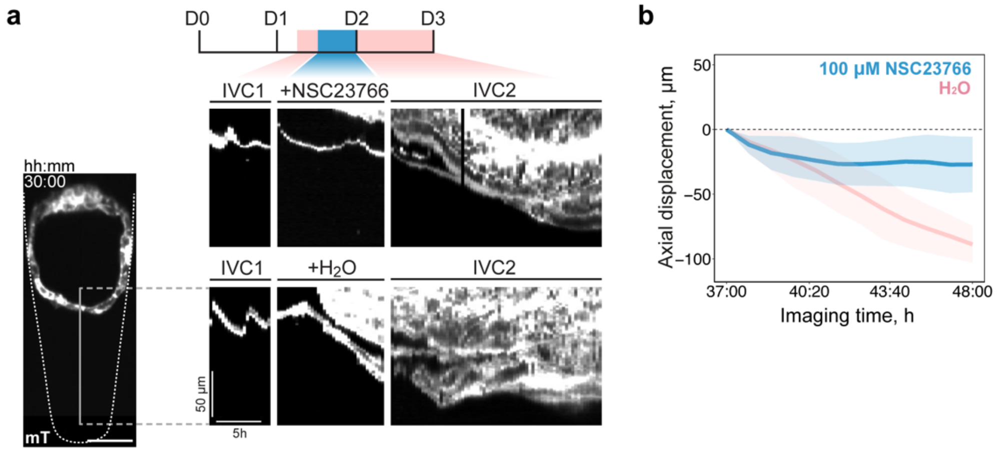

Appendix Figure S7. Pharmacological inhibition of trophoblast motility. a, Kymographs showing mural TE (mTE) leading-edge displacement along the Y-axis, indicated with the solid line on the left-most panel. Embryos from the same litter were incubated with 100 µM NSC23766 (top) and water (bottom) in IVC1 between 37 h and 48 h after recovery at E3.5; mTomato (grey). Scale bar, 50 µm. **b**, mTE leading-edge displacement along the Y-axis in embryos, incubated with 100 µM NSC23766 (blue) and water (pink) in IVC1 between 37 h and 48 h. n = 4, 4, respectively. Average values (solid lines) and standard deviations (shaded area) are shown.

Appendix Figure S8

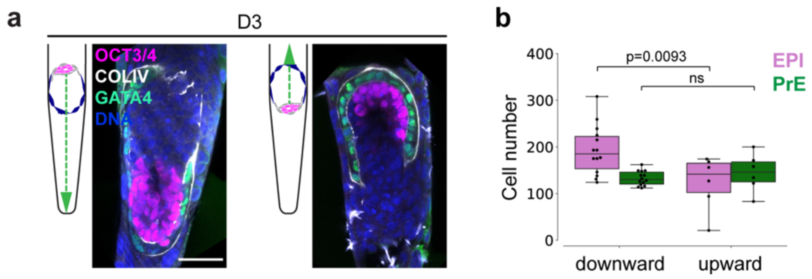

Appendix Figure S8. The effect of *ex vivo* uterine geometry on epiblast growth. a, immunofluorescence of 3E-uterus embryos from day 3 in the downward (left) and upward (right) orientations showing OCT3/4 (magenta), GATA4 (green), and nuclei (DAPI, blue). Scale bar, 50 μm. **b**, Numbers of epiblast (OCT4+, EPI) and primitive endoderm (GATA4+, PrE) cells in successfully developed 3E-uterus embryos from day 3 growing in a downward (n = 14, pooled from 3 replicates) and upward (n = 6, pooled from 4 replicates) orientations. Note lower efficiency of 3E-uterus in an upward embryo orientation (19%). Mann-Whitney's U test *P*-value.

Appendix Figure S9

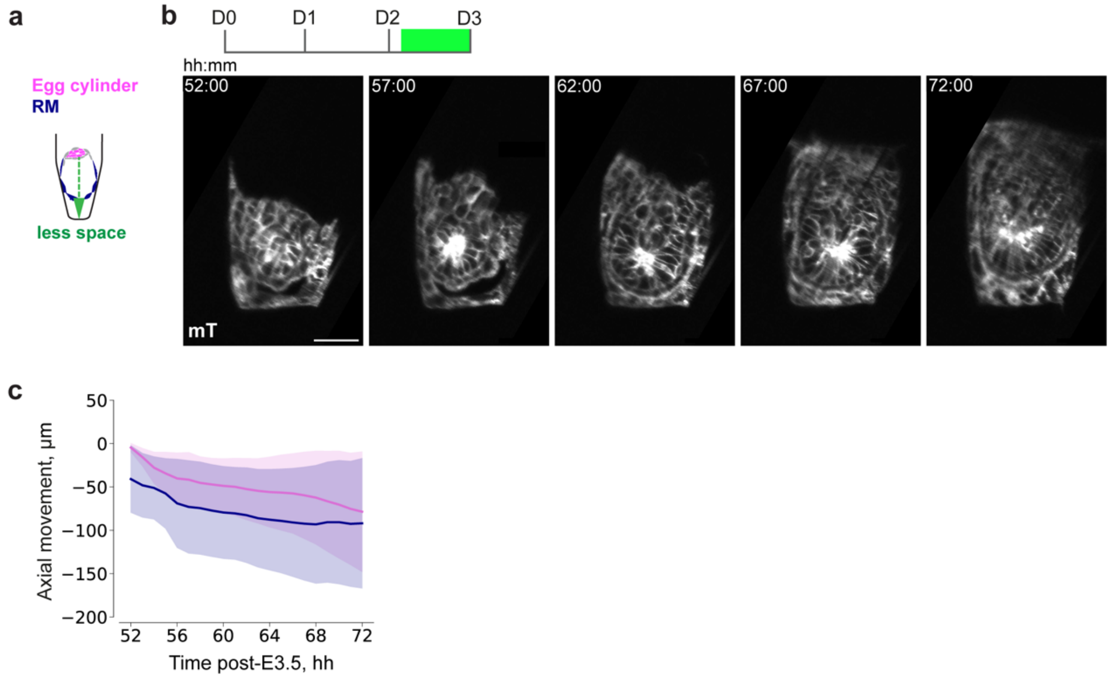

Appendix Figure S9. a, Schematic of the egg cylinder tip (magenta) and the Reichert's Membrane (RM, blue) movement within a limited space of a shallow microwell (green). Coordinates are scaled to the starting coordinate of the egg cylinder's tip. **b**, Time-lapse images of mTmG (grey) developing embryos. **c**, Movement of the egg cylinder tip (magenta) and RM (blue) along the crypt axis. Solid lines and shaded regions indicate average and SD values across several imaging planes in three imaged embryos, n = 3. t =00:00, hours: minutes from recovery at E3.5. Scale bars, 50 µm.

Appendix Table S1

Mouse Line	Primer ID	Primer Sequence	PCR Product Size, bp
mTmG and mG	oIMR7318<br>oIMR7319<br>oIMR7320	CTCTGCTGCCTCCTGGCTTCT<br>CGAGGCGGATCACAAGCAATA<br>TCAATGGGCGGGGGTCGTT	WT allele, 330; Knock- in allele, 250
H2B-GFP	CAG-Fw<br>EXFP-Rv	GGCTTCTGGCGTGTGACCGGC<br>GTCTTGTAGTTGCCGTCGTC	Tg allele, 900
Myh9-GFP	GFP-Myh9_1<br>GFP-Myh9_2<br>GFP-Myh9_3	CTGTCACATGGCTCATGTTC<br>GCCGGACACGCTGAACTTGT<br>GCCCTGAGTAGTATCGCTCC	WT allele, 400; Knock- in allele, 200
Cdx2-GFP	Cdx2-Fw<br>GFP-Rv<br>Cdx2-EX3	ATGGTTCCGTTCCCTGGTTC<br>GCGGACTTGAAGAAGTCGTGCTGCTT<br>AGGCTTGTTTGGCTCGTTACAC	WT allele, 1400; Knock- in allele, 750
Rac1-flox/del	Rac1_1<br>Rac1_2<br>Rac1_3	ATTTTGTGCCAAGGACAGTGACAAGCT<br>GAAGGAGAAGAAGCTGACTCCCATC<br>CAGCCACAGGCAATGACAGATGTTC	WT allele, 300; Flox, 330; del 130
Lifeact-GFP	LifeAct for 2<br>VenCeru-geno rev	TCAAGAAATTCGAAAGCATCTCAAAGG<br>GACCATGTGATCGCGCTTCTCGTT	Tg allele, 725
ZO1-GFP	ZO1-GFP-for<br>ZO1-GFP-rev	GCTTTCAGATGATTGTAGCC<br>GAACTTGTGGCCGTTTACGTCG	Tg allele, 400
	ZO1-WT-for<br>ZO1-WT-rev	CTTTCAGATGATTGTAGCCAGC<br>CCTTCATCAGTTCCAACAAATGC	WT allele, 420

Appendix Table S1. Genotyping primers and PCR product sizes

Appendix Table S2

	0 mM RGD	0.5 mM RGD	1 mM RGD
RGD	0 µL	5 µL	10
TEA Buffer	10	10	10
ddH2O	62.5	60	55
PEG-VS	9.65	10.45	12.09
PEG-PEP	15.35	14.55	12.91

Appendix Table S2. Composition and the recipe for PEG hydrogel preparation.

Appendix Table S3

Figure	Panel	Microscopy	Lazers	Voxel size, µm (XYZ)
**1**	a	Confocal, airyscan	405, 488, 633	0.0824 x 0.0824 x 0.1917
	b	Confocal, airyscan	405, 488, 633	0.0824 x 0.0824 x 0.1917
	d	Confocal	405, 488, 546, 633	0.207 x 0.207 x 1
	e	Confocal	405, 488, 546, 633	0.207 x 0.207 x 1
	i	Confocal	405, 488, 546	0.207 x 0.207 x 1
	j	Confocal	405, 488, 546	0.207 x 0.207 x 1
**3**	a	Confocal	405, 546, 633	0.232 x 0.232 x 1
	d	Confocal	405, 488, 633	0.232 x 0.232 x 1
	e	Confocal, airyscan	405, 488, 633	0.0824 x 0.0824 x 0.1917
	g	Confocal, airyscan	405, 488, 633	0.0824 x 0.0824 x 0.1917
	i	Confocal, airyscan	405, 488	0.0824 x 0.0824 x 0.1917
	j	InVi-SPIM	488, 561	0.104 × 0.104 × 1.000
**4**	c	MuVi-SPIM	561	0.295 × 0.295 × 1.000
	d	Confocal, airyscan	405, 488, 561	0.0824 x 0.0824 x 0.1917
	i	MuVi-SPIM	561	0.295 × 0.295 × 1.000
**5**	a	MuVi-SPIM	488, 561	0.295 × 0.295 × 2.000, 1.000
	c	MuVi-SPIM	488, 561	0.295 × 0.295 × 2.000, 1.000
	e	MuVi-SPIM	488, 561	0.295 × 0.295 × 2.000, 1.000
**6**	b	InVi-SPIM	488	0.104 × 0.104 × 1.000
	e	InVi-SPIM	561	0.104 × 0.104 × 1.000
	f	Confocal	405, 488, 546, 633	0.6919 x 0.6919 x 2
	i	MuVi-SPIM	488, 561	0.295 × 0.295 × 2.000, 1.000
	j	MuVi-SPIM	488, 561	0.295 × 0.295 × 2.000, 1.000

Appendix Table S3. Summary of the microscopy and imaging settings.

Appendix Table S4

Parameter	Prior support	Embryo 210513	Embryo 210810	Embryo 210905	Units
$\lambda_1 = \lambda_2$	[1, 100]	33 ± 3	32 ± 5	8 ± 2	γ_0 × hh
$\lambda_3 = \lambda_4$	[1, 100]	91 ± 8	29 ± 9	49 ± 9	γ_0 × hh
c_1	[−1, 1]	0.8 ± 0.1	0.6 ± 0.1	0.4 ± 0.1	γ_0
c_2	[−1, 1]	−0.5 ± 0.1	−0.6 ± 0.1	−0.9 ± 0.1	γ_0
t_0	[−0.1 τ, 1.1τ]	40.1 ± 0.9	41.5 ± 1.0	42.4 ± 0.7	Post-E3.5 hh
Δt	[0.01 τ, 2.0 τ]	11 ± 8	18 ± 10	11 ± 6	hh
a	[0, 50]	31 ± 11	26 ± 13	27 ± 13	

Appendix Table S4. Simulation-based inference of the model parameter values: dissipative coefficients for the positions of contact lines $\lambda_{1,2}$ and for the heights of embryo caps $\lambda_{3,4}$; the initial and final values of the Young tension c_1 and c_2 respectively; mid time t_0, duration Δt and modulation parameter a of the time-dependent Young tension $\Delta\gamma$ [Appendix, Eq. (S16)]; τ is the total time of experimental observations. Values of t_0 have been converted to the post-E3.5 time. Error bounds are given by one standard deviation.

APPENDIX NOTE ON THE THEORETICAL MODEL

1 Droplet wetting model of embryo implantation

A thermodynamic theory of capillary phenomena can be formulated by using the free energy H of an incompressible liquid droplet [3, Sec. 5.6]. Considering the whole embryo as such a droplet and the 3E-uterus as a solid substrate, we then pose

$$H = \gamma_0 A_0 + \Delta\gamma A_\mathrm{S} - \Delta P V, \tag{S1}$$

in which γ_0 is the surface tension between the medium and the embryo, $\Delta\gamma = \gamma_\mathrm{E} - \gamma_\mathrm{M}$ is the Young tension—the difference between the surface tension of embryo-substrate (γ_E) and medium-substrate (γ_M) contacts,—and A_0 and A_S are the areas of the embryo-medium and embryo-substrate contacts respectively (Fig. 4f). The Laplace pressure ΔP acts as a Lagrange multiplier to the volume of the embryo $V = \text{const}$.

1.1 Equilibrium solutions in cylindrical geometry

First we consider the simplest limiting case of an embryo within a cylindrical confinement and seek the equilibrium solutions for the droplet shape. Given uniform interfacial tensions, the droplet must manifest cylindrical symmetry (Fig. EV4a). For positive γ_0, the droplet-medium interface therefore takes the minimal surface corresponding to a spherical cap. We denote by h the distance between the contact lines, by y the height of the spherical caps, and by r the radius of the cylinder. When the height y is negative, the spherical caps curve inwards into the embryo. With these definitions, the contact areas and volume are given by

$$A_0 = 2\pi\left(r^2 + y^2\right), \quad A_\mathrm{S} = 2\pi r h, \tag{S2}$$

$$V = \pi h r^2 + \frac{\pi}{3} y \left(3r^2 + y^2\right). \tag{S3}$$

The value of h is determined by the volume constraint

$$h = \frac{V}{\pi r^2} - y - \frac{y^3}{3r^2}, \tag{S4}$$

and the equilibrium conditions for h and y read

$$\frac{\partial H}{\partial h} = \pi r(2\Delta\gamma - r\Delta P) = 0, \qquad \frac{\partial H}{\partial y} = \pi\left[4\gamma_0 y - \Delta P(r^2 + y^2)\right] = 0 \tag{S5}$$

which recapitulate the Laplace law for the pressure $\Delta P = 2\Delta\gamma/r$ [3, Sec. 5.6] and yield

$$y_\pm = \frac{r\gamma_0}{\Delta\gamma}\left(1 \pm \sqrt{1 - \frac{\Delta\gamma^2}{\gamma_0^2}}\right). \tag{S6}$$

Equilibrium solutions exist only for the partial wetting regime $-1 < \Delta\gamma/\gamma_0 < 1$ (Fig. EV4c). For $\Delta\gamma/\gamma_0 < -1$, the droplet spreads along the surface of the confinement completely (total wetting), whereas for $\Delta\gamma/\gamma_0 > 1$, an interface between the droplet and the substrate is not energetically favored (dewetting) and therefore a droplet with a volume below the confinement limit detaches from the substrate and takes on a spherical shape (Fig. EV4d). In the partial wetting regime, Eq. (S6) has a stable and an unstable solution y_- and y_+ respectively, for which

$$\left.\frac{\partial^2 H}{\partial y^2}\right|_{y=y_-} > 0, \qquad \left.\frac{\partial^2 H}{\partial y^2}\right|_{y=y_+} < 0. \tag{S7}$$

The contact angle $\theta = \varphi + \pi/2$ is related to the angle of the spherical cap φ and therefore

$$\cos\theta = -\sin\varphi = -\frac{r}{R} = -\frac{2ry}{r^2+y^2}, \tag{S8}$$

in which we used the formula for the radius of curvature of the spherical cap $R = (r^2+y^2)/(2y)$.

If we consider only the stable solution in Eq. (S6) we further obtain

$$\cos\theta = \frac{2\gamma_0}{\Delta\gamma}\left(1-\sqrt{1-\frac{\Delta\gamma^2}{\gamma_0^2}}\right) + \mathcal{O}\left(\frac{\Delta\gamma^2}{\gamma_0^2}\right) = -\frac{\Delta\gamma}{\gamma_0} + \mathcal{O}\left(\frac{\Delta\gamma^2}{\gamma_0^2}\right), \tag{S9}$$

where the last equality recapitulates the Young law for the wetting angle [1, Sec. II.A.1].

1.2 Shape dynamics in frustum geometry

To model the changes in trophoblast adhesion during implantation, we now consider a dynamic decrease of the interfacial tension between the embryo and the 3E-uterus, and calculate the resulting shape dynamics by taking into account the broken up-down symmetry of the conical-frustum confinement. We assume that the contact angle remains close to its equilibrium value as the adhesion of the embryo to the 3E-uterus changes.

We describe the system's state by four thermodynamic variables $\boldsymbol{z} = (z_1, z_2, z_3, z_4)$ (Fig. EV4b): positions of the top and bottom contact lines z_1 and z_2, respectively, and the heights of the top and bottom spherical caps z_3 and z_4. The radii of the frustum's horizontal sections through the contact lines are then $r_a = \chi z_1$ and $r_b = \chi z_2$, in which $\chi = \tan(\alpha/2)$ with α the conical angle.

With these definitions we obtain the following expressions for the contact areas and the volume of the droplet:

$$A_0 = \pi\left[\chi^2\left(z_1^2+z_2^2\right)+z_3^2+z_4^2\right], \quad A_S = \pi\chi\sqrt{1+\chi^2}\left(z_1^2-z_2^2\right), \tag{S10}$$

$$V = \frac{\pi}{3}\chi^2\left(z_1^3-z_2^3\right)+\frac{\pi}{6}\left[z_3\left(3\chi^2z_1^2+z_3^2\right)+z_4\left(3\chi^2z_2^2+z_4^2\right)\right]. \tag{S11}$$

The above description yields the cylindrical geometry $r_a \to r, r_b \to r$ as we take the limit $\chi \to 0$.

From the equilibrium condition for the frustum geometry ($\partial H/\partial z_{i=1,2,3,4} = 0$) we can derive the following formulas for the Laplace pressure and the Young tension:

$$\frac{\Delta P}{\gamma_0} = \frac{4z_3}{\chi^2z_1^2+z_3^2} = \frac{4z_4}{\chi^2z_2^2+z_4^2}, \tag{S12}$$

$$\frac{\Delta\gamma}{\gamma_0} = \frac{\chi}{\sqrt{1+\chi^2}}\left[\frac{2z_3(z_1+z_3)}{\chi^2z_1^2+z_3^2}-1\right] = \frac{\chi}{\sqrt{1+\chi^2}}\left[\frac{2z_4(z_2-z_4)}{\chi^2z_2^2+z_4^2}+1\right], \tag{S13}$$

which should hold independently for the upper and lower spherical caps described by equilibrium values of (z_1, z_3) and (z_2, z_4) respectively (Fig. EV4b).

Equations (S12) and (S13) applied to the experimentally measured geometry of the embryos (Sec. 2) yielded different results for the upper and lower caps, (z_1, z_3) and (z_2, z_4) respectively, which are thus inconsistent with the equilibrium state. Therefore we concluded that the observed series of $\boldsymbol{z}(t)$ outline a sequence of nonequilibrium states as discussed shortly below.

Assuming a linear constitutive relation between the energy gradient and the velocities of the variables $z_{i=1,2,3,4}$ with dissipative coefficients $\lambda_{i=1,2,3,4}$, we obtain equations of motion

$$\lambda_i\dot{z}_i = -\frac{\partial H}{\partial z_i} = F_i + \Delta P C_i, \tag{S14}$$

in which

$$F_i = -\frac{\partial(\gamma_0 A_0 + \Delta\gamma A_\mathrm{S})}{\partial z_i}, \qquad C_i = \frac{\partial V}{\partial z_i}. \tag{S15}$$

The Young tension $\Delta\gamma(t)$ is now a time-dependent active parameter, describing the increase in adhesion between the embryo and the substrate. We describe the dependence of the Young tension on time t by a generic sigmoid shape over a finite time domain (Fig. EV4e):

$$\frac{\Delta\gamma(t)}{\gamma_0} = \begin{cases} c_1 \text{ if } t \leq t_1, \\ c_2 \text{ if } t \geq t_2, \\ c_1 + (c_2 - c_1) I_{\frac{t-t_1}{t_2-t_1}}(2+a, 2+a) \text{ otherwise,} \end{cases} \tag{S16}$$

in which c_1 and c_2 are constant values, t_1 and t_2 are respectively the beginning and the end of the adhesion decrease with the mid time $t_0 = (t_1 + t_2)$, $a \in [0, +\infty)$ is a modulation parameter, whereas $I_x(\alpha, \beta)$ is the incomplete regularized beta-function (the cumulative beta-distribution function with shape parameters α and β).

Furthermore, during the implantation process the embryo also regulates its volume, which can be incorporated into our model by making the volume constraint time-dependent:

$$V(\boldsymbol{z}) = V_0(t). \tag{S17}$$

The value of the Lagrange multiplier ΔP can be determined by differentiating the constraint Eq. (S17),

$$\dot{V}_0(t) = \sum_{i=1}^{4} \frac{\partial V}{\partial z_i}\, \dot{z}_i = \sum_{i=1}^{4} \frac{C_i}{\lambda_i}\,(F_i + \Delta P C_i) \tag{S18}$$

which is solved by

$$\Delta P = \frac{\dot{V}_0 - \sum_{i=1}^{4} C_i F_i/\lambda_i}{\sum_{i=1}^{4} C_i^2/\lambda_i}. \tag{S19}$$

Given an initial condition $\boldsymbol{z}(0)$ and the functions $\Delta\gamma(t)$ and $\dot{V}_0(t)$, the equations of motion (S14) can be integrated for $\boldsymbol{z}(t)$.

In the course of motion defined by Eq. (S14) the free energy changes as

$$\begin{aligned} dH &= \sum_{i=1}^{4} \frac{\partial H}{\partial z_i}\, dz_i + \frac{\partial H}{\partial \Delta\gamma} d\Delta\gamma = -\sum_{i=1}^{4} (F_i + \Delta P C_i)\, dz_i + \frac{\partial H}{\partial \Delta\gamma} d\Delta\gamma \\ &= -\sum_{i=1}^{4} F_i dz_i - \Delta P dV + \frac{\partial H}{\partial \Delta\gamma} d\Delta\gamma = dQ + dW, \end{aligned} \tag{S20}$$

which recapitulates the first law of thermodynamics if we identify the heat and the active work, respectively,

$$dQ = -\sum_{i=1}^{4} F_i dz_i, \qquad dW = -\Delta P dV + \frac{\partial H}{\partial \Delta\gamma} d\Delta\gamma. \tag{S21}$$

The two active contributions of the work $dW = dW_1 + dW_2$ correspond to the volume change and the adhesion change:

$$dW_1 = -\Delta P dV, \qquad dW_2 = \frac{\partial H}{\partial \Delta\gamma} d\Delta\gamma. \tag{S22}$$

Note that, when the volume V is conserved, i.e. $V_0(t) \equiv$ const, the forces of constraint remain perpendicular to the system's trajectory $\boldsymbol{dz}(t)$ and thus do no work ($dW_1 = 0$). This observation follows from the geometric analysis of Eqs. (S14)–(S19), which are entirely analogous to the isokinetic thermostat in molecular dynamics as discussed by Evans and Morriss [2, Sec. 5.2].

Given that the dissipative coefficients λ_i are large compared to the speed of the adhesion change $|c_2 - c_1|/(t_2 - t_1)$ (Fig. EV4e), the system relaxes slowly in the response to the Young tension change and, thus, the observed time series of $\boldsymbol{z}(t)$ may correspond to transient states substantially far from equilibrium.

2 Comparison with experimental data

Time series were acquired with a time resolution of 1 hour for the whole-embryo volume V_0, as well as for the contact angles, $\theta_a(\omega_i)$ and $\theta_b(\omega_i)$, and positions of the top and bottom contact lines with respect to z_0, $x_a(\omega_i)$ and $x_b(\omega_i)$, at several points $\omega_{i=1,2...}$ around the conical axis. The heights of the spherical caps were estimated as

$$y_a(\omega_i) = \frac{\chi x_a(\omega_i)}{\sin \varphi_a(\omega_i)} \left[1 - \cos \varphi_a(\omega_i)\right], \qquad y_b(\omega_i) = \frac{\chi x_b(\omega_i)}{\sin \varphi_b(\omega_i)} \left[1 - \cos \varphi_b(\omega_i)\right], \tag{S23}$$

in which

$$\varphi_a(\omega_i) = \theta_a(\omega_i) - (\pi - \alpha)/2, \qquad \varphi_b(\omega_i) = \theta_b(\omega_i) - (\pi + \alpha)/2. \tag{S24}$$

By averaging the above measurements over the points ω_i we find the time series for the thermodynamic variables of interest

$$z_1 = \langle x_a \rangle_\omega, \qquad z_2 = \langle x_b \rangle_\omega, \qquad z_3 = \langle y_a \rangle_\omega, \qquad z_4 = \langle y_b \rangle_\omega, \tag{S25}$$

and their standard deviations $\sigma_{i=1,2,3,4}$.

A smooth representation of the embryo volume was constructed by interpolating the experimentally measured volume with

$$V_0(t) = \eta_0 t + \sum_{k=1}^{K-1} \eta_k \sin\left(\frac{\pi k t}{\tau}\right), \tag{S26}$$

in which τ is the total observation time, and K is the number of timepoints, which yields a derivative with a spectral accuracy [5, Chapter 4]

$$\dot{V}_0(t) = \eta_0 + \sum_{k=1}^{K-1} \frac{\pi k \eta_k}{\tau} \cos\left(\frac{\pi k t}{\tau}\right). \tag{S27}$$

The frustum-angle tangent is $\chi = 0.1$, as measured from the imaging data.

2.1 Simulation-based inference

To determine the dissipative coefficients $\lambda_{i=1,2,3,4}$ and parameters of the Young tension $\Delta\gamma(t)$ given by Eq. (S16) we used simulation-based inference [4] with the time series of $\boldsymbol{z}(t)$, the standard deviations $\boldsymbol{\sigma}(t)$, and the volume derivative $\dot{V}_0(t)$, which were acquired from the experiments as described above. Furthermore we assume $\lambda_1 = \lambda_2$, $\lambda_3 = \lambda_4$, and $|\Delta\gamma| \leq \gamma_0$. Because time-series of the geometric data do not provide complete information about quantities involving energy and mass, we adopt a custom system of physical units based on hours for time, μm for length, and γ_0 for tension.

In total we have seven fitting parameters: λ_1, λ_3, c_1, c_2, t_0, $\Delta t = t_2 - t_1$, and a (Table S4). Assuming uniform prior distributions of these parameters, we applied two rounds of sequential neural posterior estimation [4] with

10^6 simulations in each round and Gaussian kernel-mixture representation of the probability density. The neural posterior estimator was trained directly on the time series $\boldsymbol{z}(t)$ generated by Eq. (S14) with the experimentally determined $\dot{V}_0(t)$ and a superimposed random Gaussian error of the zero mean and the observed standard deviation $\boldsymbol{\sigma}(t)$. The parameter values thus estimated are mostly consistent across the three embryos for which we have measurements, with a somewhat larger variability of the dissipative coefficients λ_i.

2.2 Model validation

To check how well our model describes the experimental data, we used a leave-one-out validation test as follows. We leave one of the three embryos as a trial example, and average values of the parameters (λ_1, λ_3, c_1, c_2, t_0, $\Delta t = t_2 - t_1$, and a) fitted to the two other embryos. Using these average values we attempt to predict dynamics of the contact line in the trial embryo. For three embryos we obtain three such tests (Appendix Fig. S4).

Overall our model captures the dynamics of contact lines between the embryo and 3E-uterus. It reasonably well predicts the onset and duration of the implantation process. Within three standard deviations the experimental measurements of contact lines' positions agree with our predictions in the validation tests of two embryos (Appendix Fig. S4a–b), whose fitted parameters are also close by value in Table S4. The onset of the contact-angle decrease in the third embryo is also predicted by our model (Appendix Fig. S4c), but at later stages the measured contact angle decreases more rapidly.

References

[1] P. G. de Gennes. Wetting: statics and dynamics. *Rev. Mod. Phys.*, 57:827–863, Jul 1985. doi: 10.1103/RevModPhys.57.827.

[2] D.J. Evans and G.P. Morriss. *Statistical Mechanics of Nonequilibrium Liquids.* DOAB Directory of Open Access Books. ANU E Press, 2007. ISBN 9781921313233.

[3] D. Kondepudi and I. Prigogine. *Modern Thermodynamics: From Heat Engines to Dissipative Structures.* CourseSmart Series. Wiley, 2014. ISBN 9781118371817.

[4] Alvaro Tejero-Cantero, Jan Boelts, Michael Deistler, Jan-Matthis Lueckmann, Conor Durkan, Pedro J. Gonçalves, David S. Greenberg, and Jakob H. Macke. sbi: A toolkit for simulation-based inference. *Journal of Open Source Software*, 5(52):2505, 2020. doi: 10.21105/joss.02505.

[5] L.N. Trefethen. *Spectral Methods in MATLAB.* Software, Environments, and Tools. Society for Industrial and Applied Mathematics, 2000. ISBN 9780898714654.
