# Supplementary material for: Embryo‐uterine interaction coordinates mouse embryogenesis during implantation
Source: EMBO J. 2023 Jul 31;42(17):e113280. doi: 10.15252/embj.2022113280 (PMC10476174; doi:10.15252/embj.2022113280)
Supplement: Supplementary file 2 — Expanded View Figures PDF [file EMBJ-42-e113280-s003.pdf]

Expanded View Figures

Figure EV1. ***Ex vivo*** **Engineering Uterine Environment with topographically patterned hydrogels.**

A Schematic showing the lineages and the layers of extracellular matrix, comprising *in utero* peri-implantation mouse embryo at E5.25. EPI, epiblast; ExE, extraembryonic ectoderm; TB, trophoblast; PE, parietal endoderm; VE, visceral endoderm; BM, the basal membrane between EPI/ExE and VE; RM, Reichert's membrane; the embryo is surrounded by maternal decidua, the egg cylinder is delineated with dashed lines.

B Immunostaining of E4.75 (top) and E5.25 (bottom) pregnant uteri cross sections, showing Fibronectin (FN1, white), Collagen IV (COLIV, white), and Laminin (LAM, white) (from left to right), GATA4 (green), and nuclei (DNA, blue). White asterisks mark the implanted embryos. Red arrowheads point at the uterine ECM.

C Schematic of the 3D hydrogel-embedded embryo culture. *Inset*, Immunostaining of the embryos embedded and cultured 3D inside hydrogel drops until Day 2 (D2) and Day 3 (D3) showing OCT3/4 (magenta), GATA4 (green), and nuclei (DAPI, blue). White arrowheads point at the Reichert's membrane.

D Comparison of the epiblast (EPI) cell numbers between *in utero* E3.5–E5.5 embryos and embryos embedded and cultured 3D inside hydrogel drops until Days 2–3 (D2–3). $n = 7$ (D2) and $n = 14$ (D3). The midline marks the median, and the boxes indicate the interquartile range. Mann–Whitney's U test *P*-value.

E Schematic of the embryo morphology criteria (I-III), based on which the efficiency of the *ex vivo* culture is evaluated.

E′ Immunostaining of 3E-uterus embryos from Day 3 showing OCT3/4 (magenta), GATA4 (green), Laminin (LAM, white), and nuclei (DNA, blue). The embryos that form egg cylinder (I) show the egg cylinder axis in line with the crypt axis (II), and form Reichert's membrane (III), are considered to be successfully developed (outlined in green; 46%; $n = 12$ of 26, pooled from three independent experiments). White arrowheads point at Reichert's membrane.

F 3E-uterus efficiency for embryo culture inside cylindrical crypts with different diameters, calculated across 2 (80 μm), 3 (100 μm), 3 (120 μm), 5 (140 μm), and 3 (160 μm) independent experiments.

G 3E-uterus efficiency for embryo culture inside funnel-shaped microwells made of nonbiodegradable PEG with RGD, calculated across 2 (1.5% PEG concentration), 2 (1.7%), 2 (2%), and 1 (3%) independent experiments.

H Immunostaining of 3E-uterus embryos from Day 3 (D3) grown in 1.5%, 2%, 2.5%, and 7% PEG precursor concentrations (from left to right), showing OCT3/4 (magenta), GATA4 (green), Collagen IV (COLIV, white), and nuclei (DNA, blue).

I 3E-uterus efficiency at Day 3 at a 1.5–7% range of PEG precursor content, calculated across 3 (1.5%), 4 (1.75%), 3 (2%), 3 (2.25%), 3 (2.5%), 3 (2.75%), 3 (6%), and 2 (7%) independent experiments. Inset, rheological measurement showing linear relationship between the PEG precursor content (%, w/v) and the Shear modulus (kPa).

J Total cell number (EPI + VE) vs *in utero* developmental stage. The days of 3E-uterus culture were matched with the *in utero* stages based on the log-linear regression. Equation of the regression line for the total cell number (EPI and VE) is y = 0.133e1.489x; that for the EPI cell number is y = 0.036e1.617x. $n = 6$ (E3.5), $n = 21$ (E4.5), $n = 28$ (E4.75), $n = 20$ (E5.0), $n = 20$ (E5.25), $n = 21$ (E5.5), $n = 21$ (E5.75) and 22 (E6.0). Y scale, log 10.

K Immunostaining of E5.25 pregnant uterus cross section (left) and 3E-uterus embryo from Day 3 (right) showing H2B-GFP (marks the embryo in green), Cytokeratin 8 (KRT8, red), pan-Laminin (pan-LAM, white), and nuclei (DNA, blue). Right, 4× zoom; bottom, 2× zoom. White arrowheads point at Reichert's membrane. Scale bars, 50 μm, 100 μm (A), 12.5 μm (J, zoom-in).

© 2023 The Authors

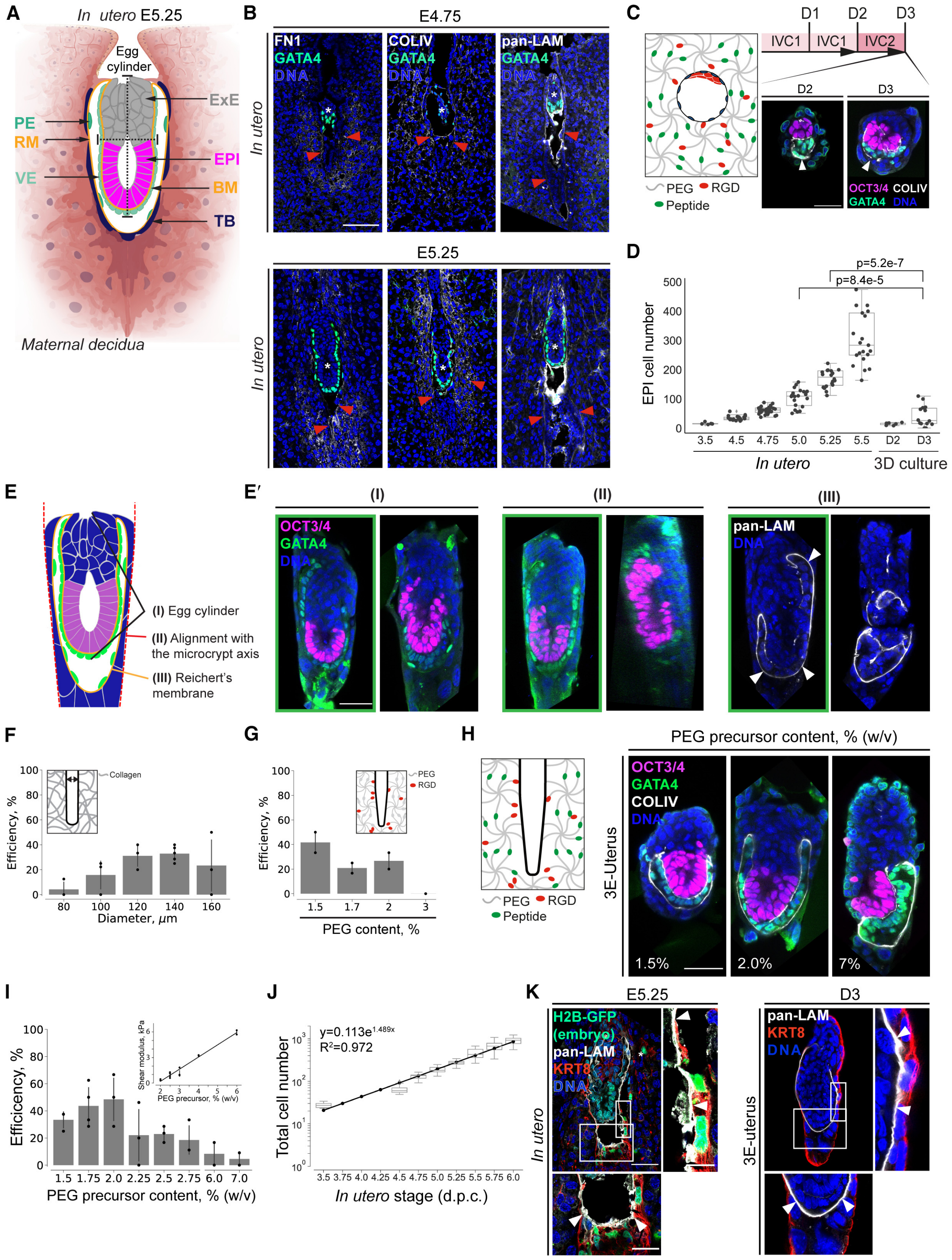

Figure EV1.

© 2023 The Authors

Figure EV2. Expression of the lineage marker genes.

A–D UMAPs colored by the normalized expression of epiblast (A, EPI), polar trophectoderm/extraembryonic ectoderm (B, pTE/ExE), visceral, anterior visceral, and parietal endoderm (C, VE/AVE/PE), and mural trophectoderm/trophoblast (D, mTE/TB) across *In utero* (top, n = 566) and 3E-uterus (bottom, n = 668) cells.

E The UMAP colored by the experimental conditions: 3E-uterus (D2, light blue; D3, dark blue) and *in utero* (E.4.5, red; E5.25, yellow), total n = 1,234.

F The numbers of single-cell transcriptomes per experimental condition and cell type.

© 2023 The Authors

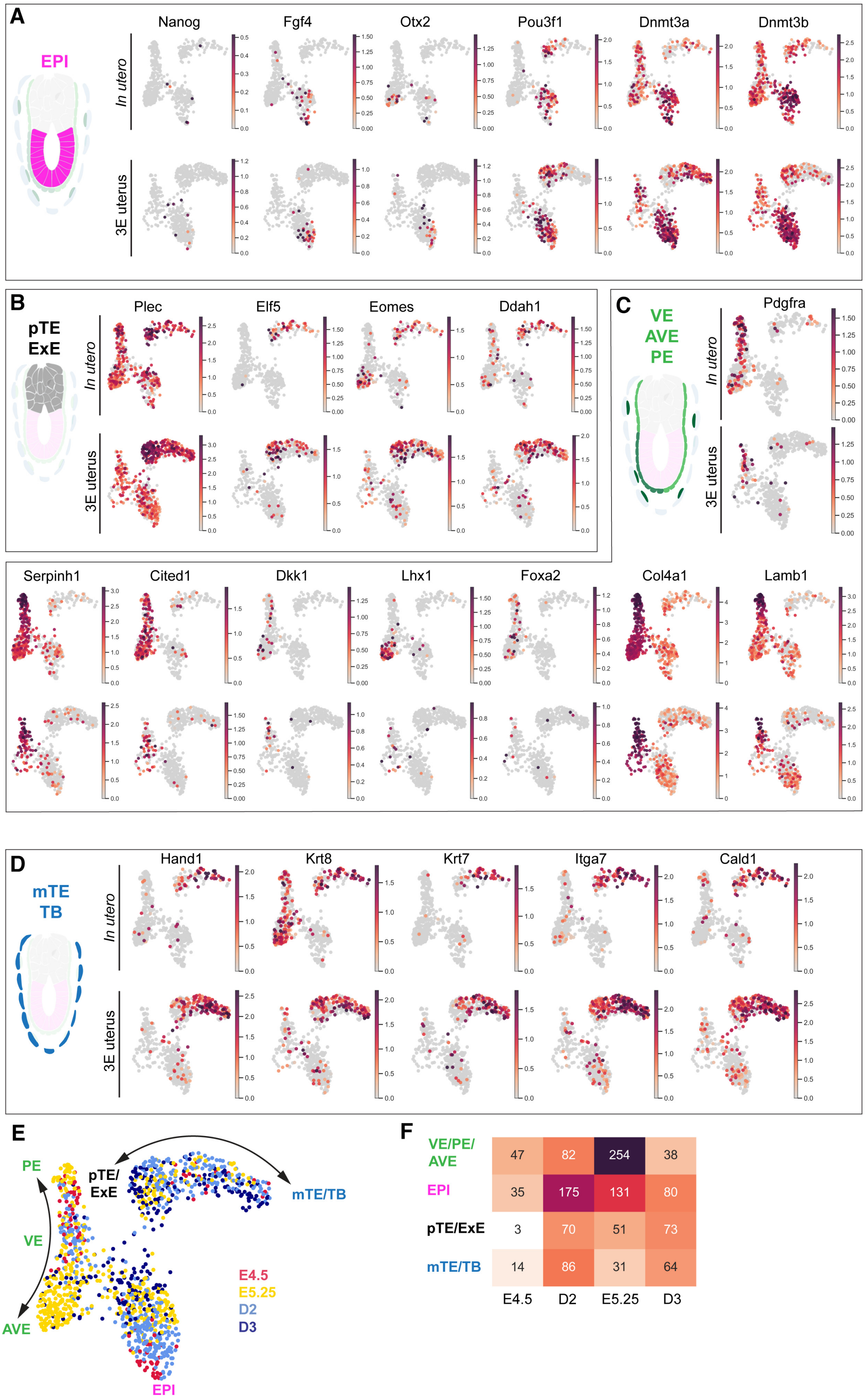

Figure EV2.

 © 2023 The Authors

Figure EV3. Characterization of trophoblast cell polarity and cytoskeletal dynamics.

A, B Immunofluorescence of E4.5 embryos showing nuclei (DNA, blue), integrin beta 1 (ITGB1, red) (A), and active ITGB1 (12G10, green) (B). Right, 4× zoom-ins. Arrowheads point to the apicobasal integrin localization in mural TE.

C Immunofluorescence of the blastocyst-stage embryo (E3.5) showing integrin beta 1 (ITGB1, red), and nuclei (DAPI, blue). Right, 4× zoom. Arrowheads point to the basal integrin localization in TE.

D 3D projections of time-lapse images of the developing ZO1-GFP (green);mTmG (magenta) embryo. Bottom, 2.5× zoom into the TE cell; white arrowheads mark cell–cell interfaces.

E Immunofluorescence of the 3E-uterus embryo after live imaging, simultaneously stained for ZO1-GFP (green) PARD6B (red), and nuclei (DNA, blue). From left to right, ZO1-GFP, PARD6B, composite image channels. Bottom, 4× zoom of the TB cell.

F Intensity profile of ZO1 and PARD6B signals along the cell surface outlined in (E, bottom), including apical and basolateral regions.

G Time-lapse images of the developing Myh9-GFP (green);mTmG (magenta) embryo. The crypt surface is outlined.

H Immunofluorescence of the 3E-uterus embryo after live imaging showing Myh9-GFP (green) phosphor-MLC (T18/S19) (red), and nuclei (DNA, blue). Bottom, 2× zoom. White arrowheads point at the apical TB cell surface.

I Immunofluorescence of Day 3 3E-uterus embryo, showing maximum Z-projection of F-actin signal (white). Bottom, 2× zoom; right, 4× zoom. Yellow arrows mark trophoblast cell membrane protrusions. Invasive trophoblast cell protrusions at least 10 µm deep inside the biodegradable LDTM PEG matrix are consistently observed in 86% of all WT embryos at the Day 3 of 3E-uterus.

J, K immunofluorescence of the mural TE (mTE) cell of the embryo grown *in utero* until E4.5 (left) and 3E-uterus embryo from Day 2 (right) showing ZO-1 (green), phosphor-Ezrin/Radixin/Moesin (pERM, red), and nuclei (DNA, blue) without the outline, corresponding to Fig 3E and G. t = 00:00, Hours: Minutes from recovery at E3.5. Scale bars, 50 µm, 25 µm (2× zoom), 20 µm (2.5× zoom), 12.5 µm (4× zoom).

© 2023 The Authors

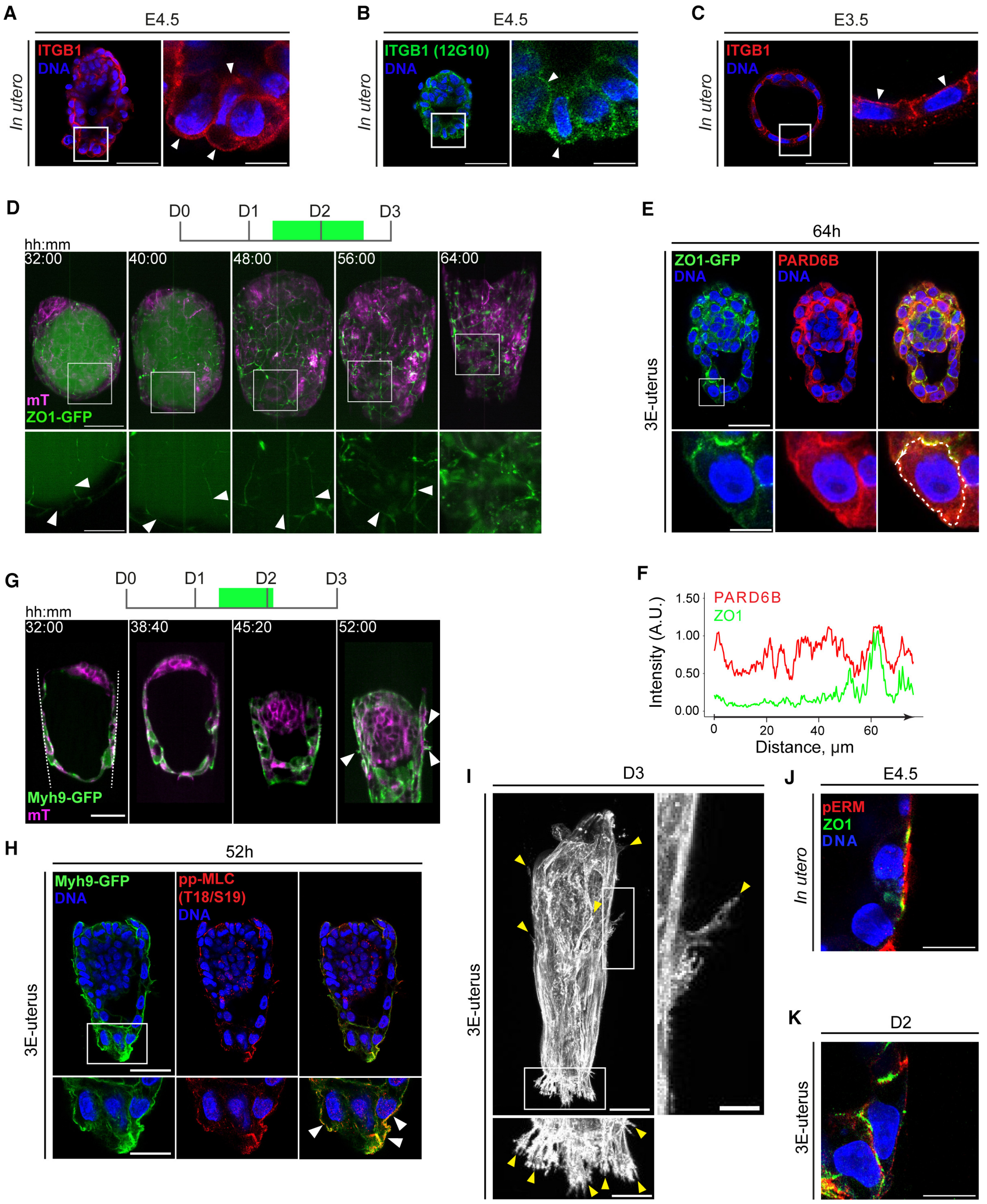

Figure EV3.

 © 2023 The Authors

Figure EV4. Characterization of the droplet-wetting model.

A The equilibrium shape of the droplet in a cylindrical confinement of radius r is described by the distance h between the two contact lines and by the height y of the top and bottom spherical caps, corresponding to polar and mural TE, respectively. These caps can also be characterized by the curvature radius R and angle φ. The contact angle θ depends on the droplet–medium tension γ_0 and the Young tension $\Delta\gamma$.

B The droplet in a conical frustum with angle α is described by the positions of the top and bottom contact lines z_1, z_2 measured from the conical tip $z_0 = 0$, and by the heights of the top and bottom spherical caps z_3 and z_4, respectively. When the caps curve into the embryo, their heights assume negative values.

C Bifurcation diagram for the equilibrium solutions Equation S6 (Appendix) of the droplet in cylindrical confinement. The solid line corresponds to the stable solution y_{-}, whereas the dashed line denotes the unstable branch y_{+}.

D Top, Calculated equilibrium shapes of the droplet in cylindrical confinement at the transition to total wetting (I), in the regime of partial wetting (II, III), and dewetting (IV). Bottom, Time-lapse images of mTmG signal (magenta) in the embryos growing in 3E-uterus with RGD, corresponding to the I-III wetting regimes and without RGD, corresponding to dewetting (IV). T = 00:00, hours: minutes after recovery at E3.5. The crypt surface is outlined.

E Sigmoid model of the Young tension adaptation Equation S16 drawn for three values of the modulation parameter $a \geq 0$. Constants c_1 and c_2 specify the initial and final values of the normalized tension. The adaptation begins at a time instance t_1 and ends at a time instance t_2. A full specification of the model requires five constants, for example, the mid-time $t_0 = (t_1 + t_2)/2$, the duration $\Delta t = t_2 - t_1$, the constants c_1 and c_2, and the modulation parameter a.

F Volume dynamics in the developing embryos between 36 and 56 h after E3.5. Colors correspond to different embryos; $n = 3$.

G Contact angle (θ_b) dynamics in developing embryos. Colors correspond to different embryos imaged in time intervals between 20 and 72 h after E3.5; $n = 10$.

H From left to right, simulated contact angle dynamics for constant tension (dashed line), and decreasing tension (solid line), with experimental data points (green and red points for θ_a and θ_b, respectively) for three different embryos between 36 and 56 h from recovery at E3.5. Error bars denote SEM. Scale bar, 50 µm.

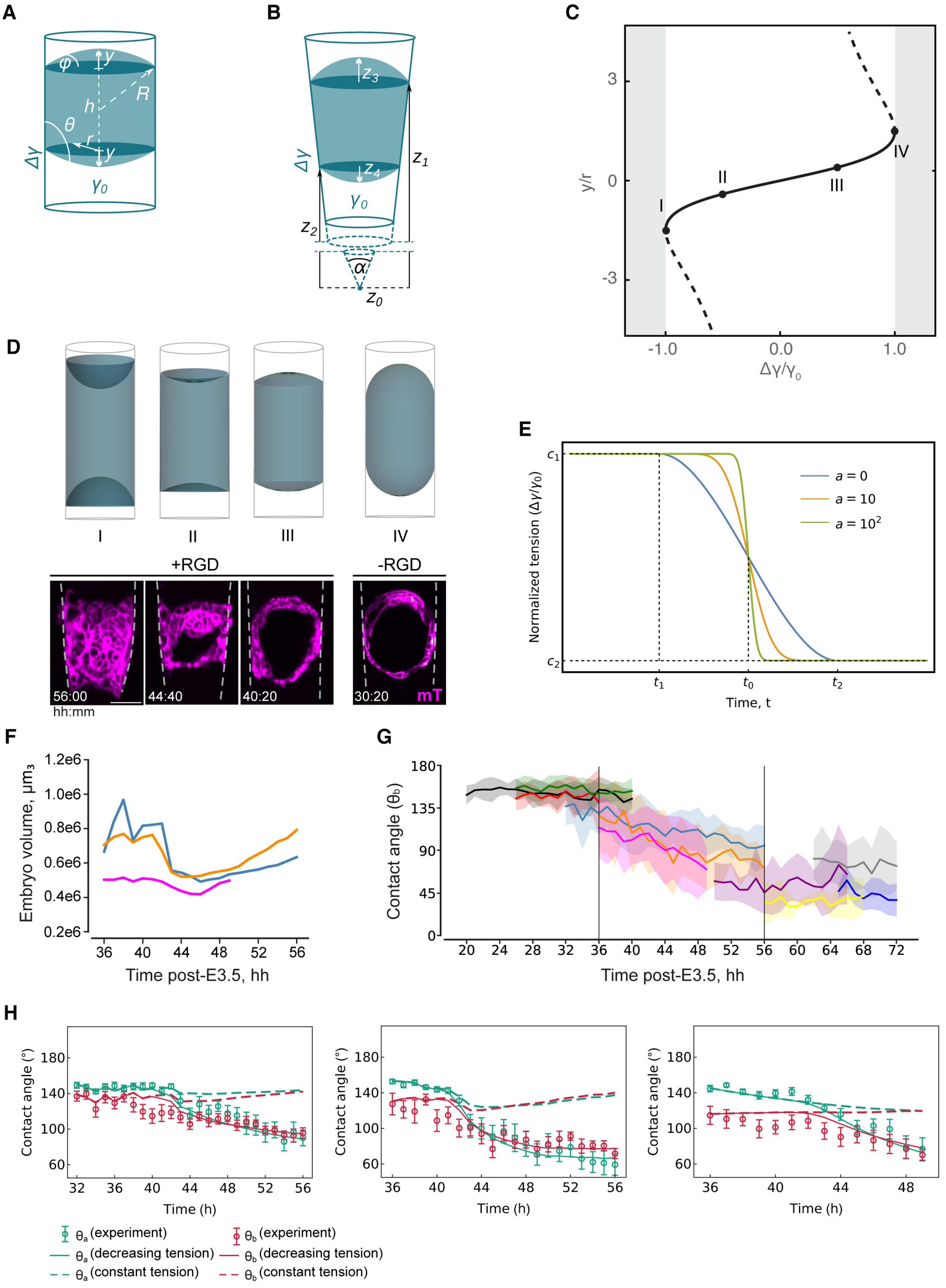

Figure EV4.

 © 2023 The Authors

Figure EV5. Characterization of collective trophoblast migration.

A Directionality of the mTE/TB migration along the X, Y, and Z axes (green, red, and blue, respectively) between subsequent hours of live imaging. $n = 29$.

B Left, Mural TE (mTE) cell trajectories for three different embryos; coordinates in XY plane are normalized to the starting coordinates. End coordinates are marked with red dots. Right, Displacement of mTE cells along the Y-axis vs imaging time post-E3.5. From top to the bottom, $n = 61, 58, 51$, respectively. The linear regression fit is shown as a black line.

C Directionality of the mTE/TB migration along the X, Y, and Z axes (green, red, and blue, respectively) between subsequent hours of live imaging for three embryos (from top to bottom). $n = 61, 58, 51$, respectively.

D Distribution density of the average TB velocities (μm/h). $n = 255$, pooled from six embryos.

E TB migration speed (μm/h) vs imaging time post-E3.5. Colors correspond to the three embryos from (B) and (C).

F Persistence of the nearest mTE/TB four-cell neighborhood between subsequent hours of live imaging.

© 2023 The Authors

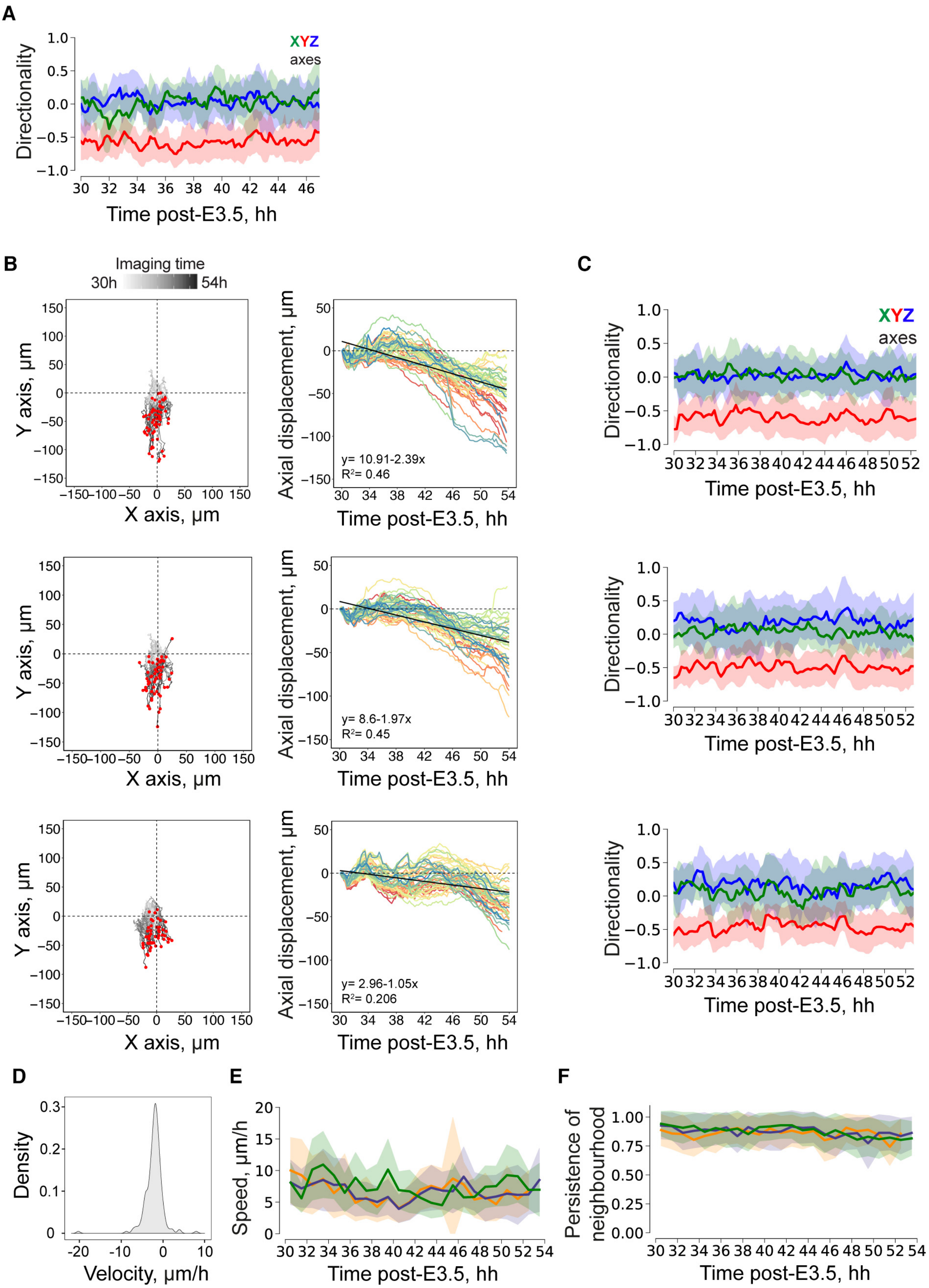

Figure EV5.

© 2023 The Authors
