# Supplementary figures and images for: Embryo‐uterine interaction coordinates mouse embryogenesis during implantation
Source: EMBO J. 2023 Jul 31;42(17):e113280. doi: 10.15252/embj.2022113280 (PMC10476174; doi:10.15252/embj.2022113280)

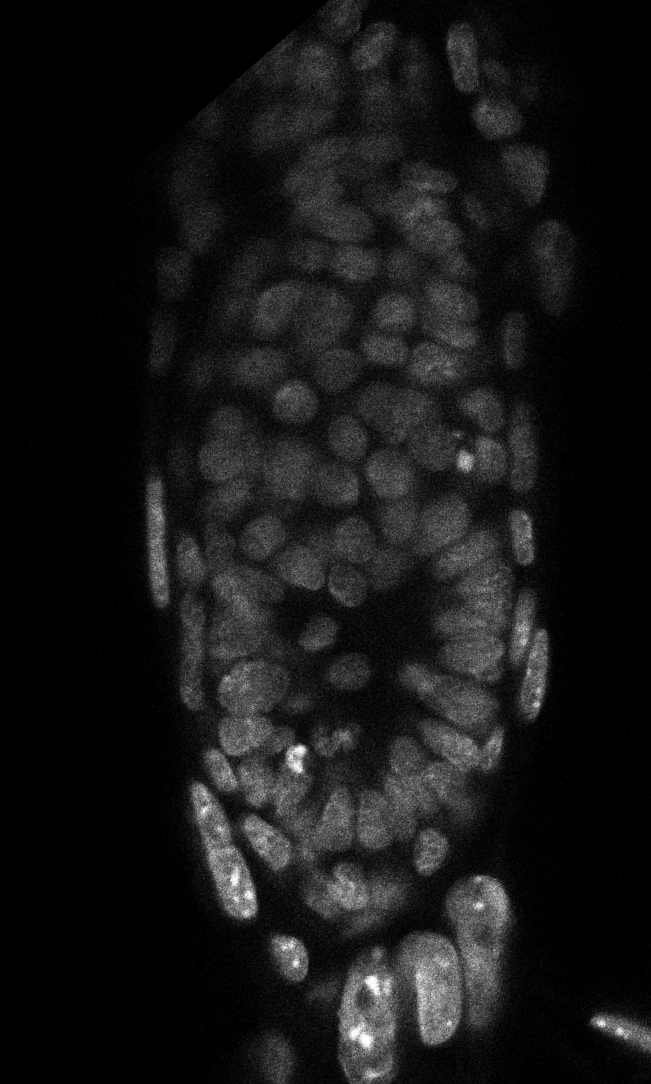

Supplement: Supplementary file 14 — Source Data for Figure 1 [file EMBJ-42-e113280-s011.zip › Figure 1/Panel e/D3_3E-uterus.tif]

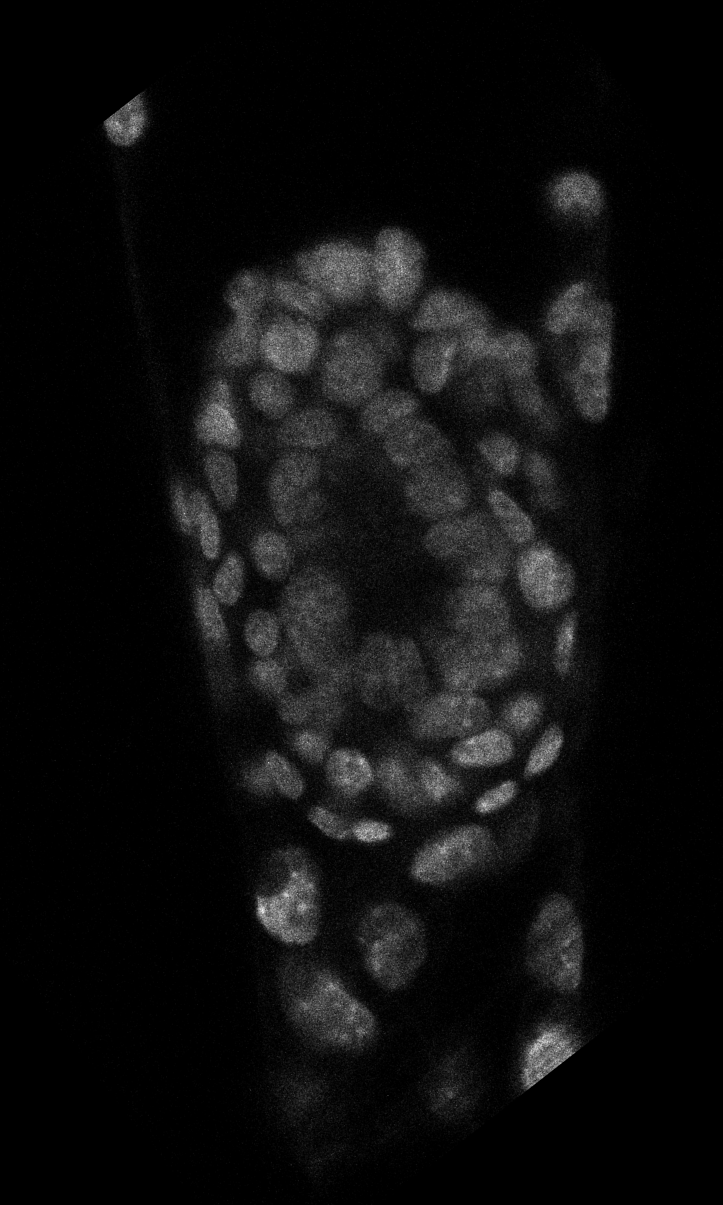

Supplement: Supplementary file 14 — Source Data for Figure 1 [file EMBJ-42-e113280-s011.zip › Figure 1/Panel e/D2_3E-uterus.tif]

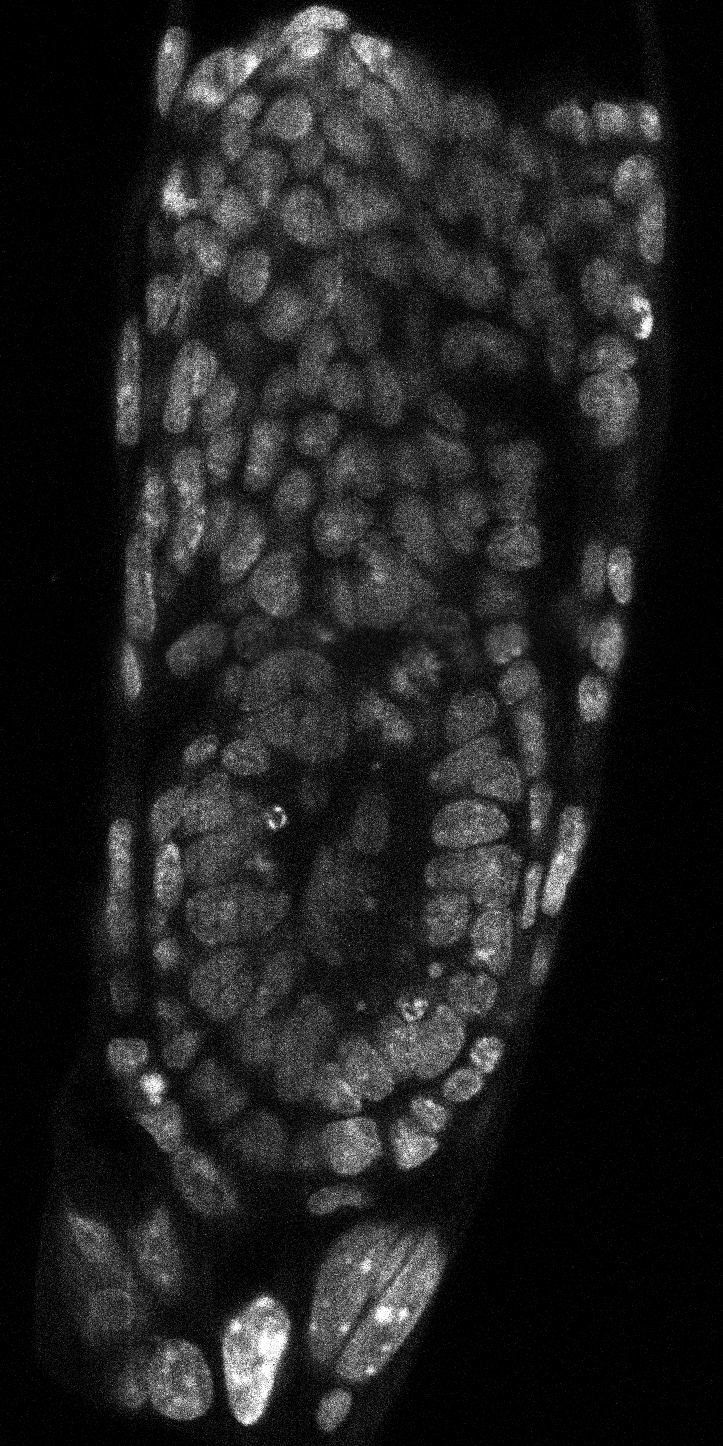

Supplement: Supplementary file 14 — Source Data for Figure 1 [file EMBJ-42-e113280-s011.zip › Figure 1/Panel j/D3_3E-uterus.tif]

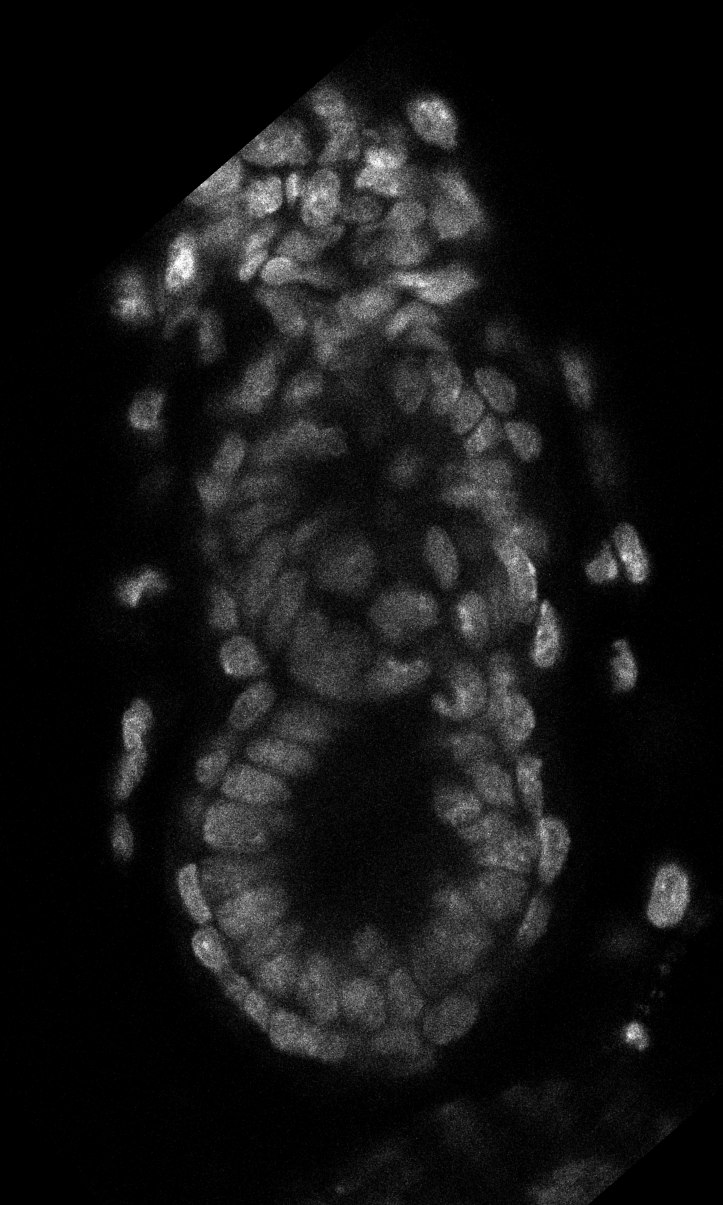

Supplement: Supplementary file 14 — Source Data for Figure 1 [file EMBJ-42-e113280-s011.zip › Figure 1/Panel d/E5.25_in_utero.tif]

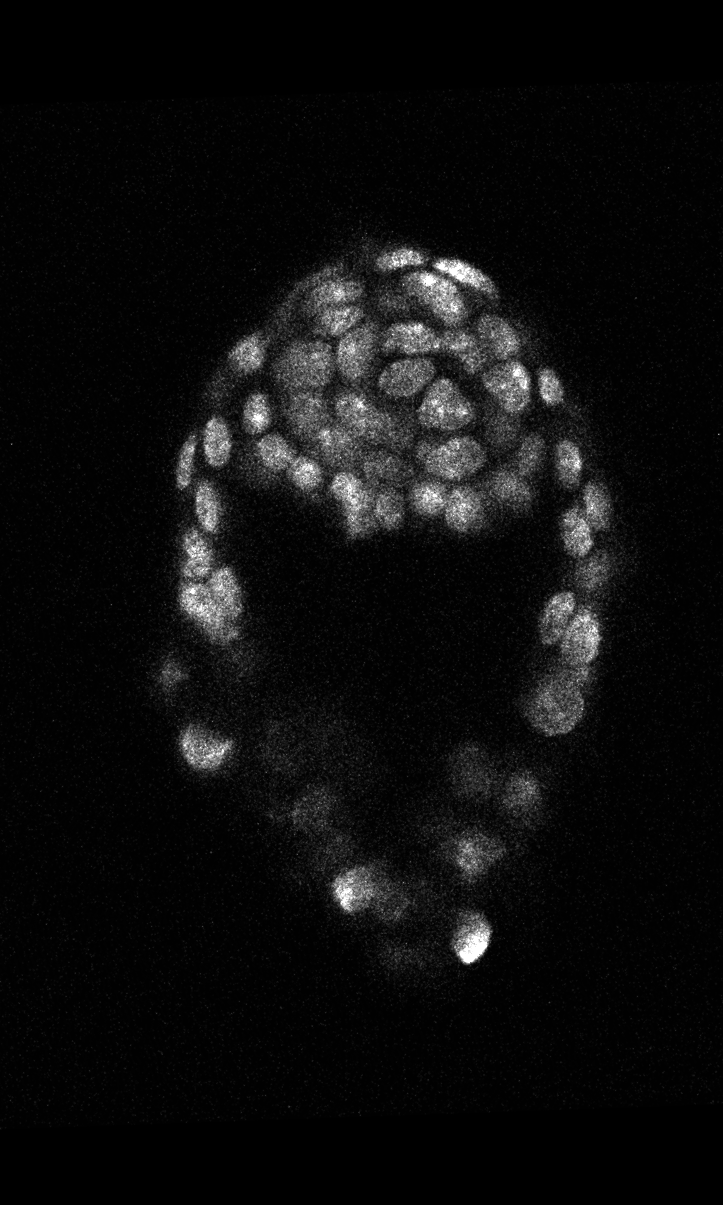

Supplement: Supplementary file 14 — Source Data for Figure 1 [file EMBJ-42-e113280-s011.zip › Figure 1/Panel d/E4.5_in_utero.tif]

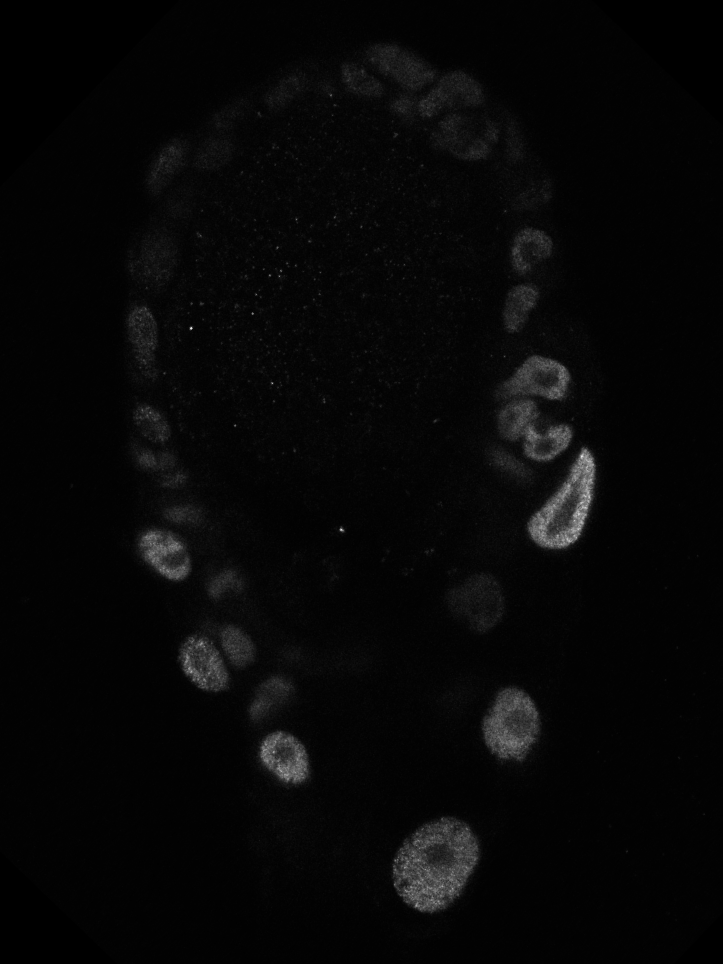

Supplement: Supplementary file 14 — Source Data for Figure 1 [file EMBJ-42-e113280-s011.zip › Figure 1/Panel i/D2_3E-uterus.tif]

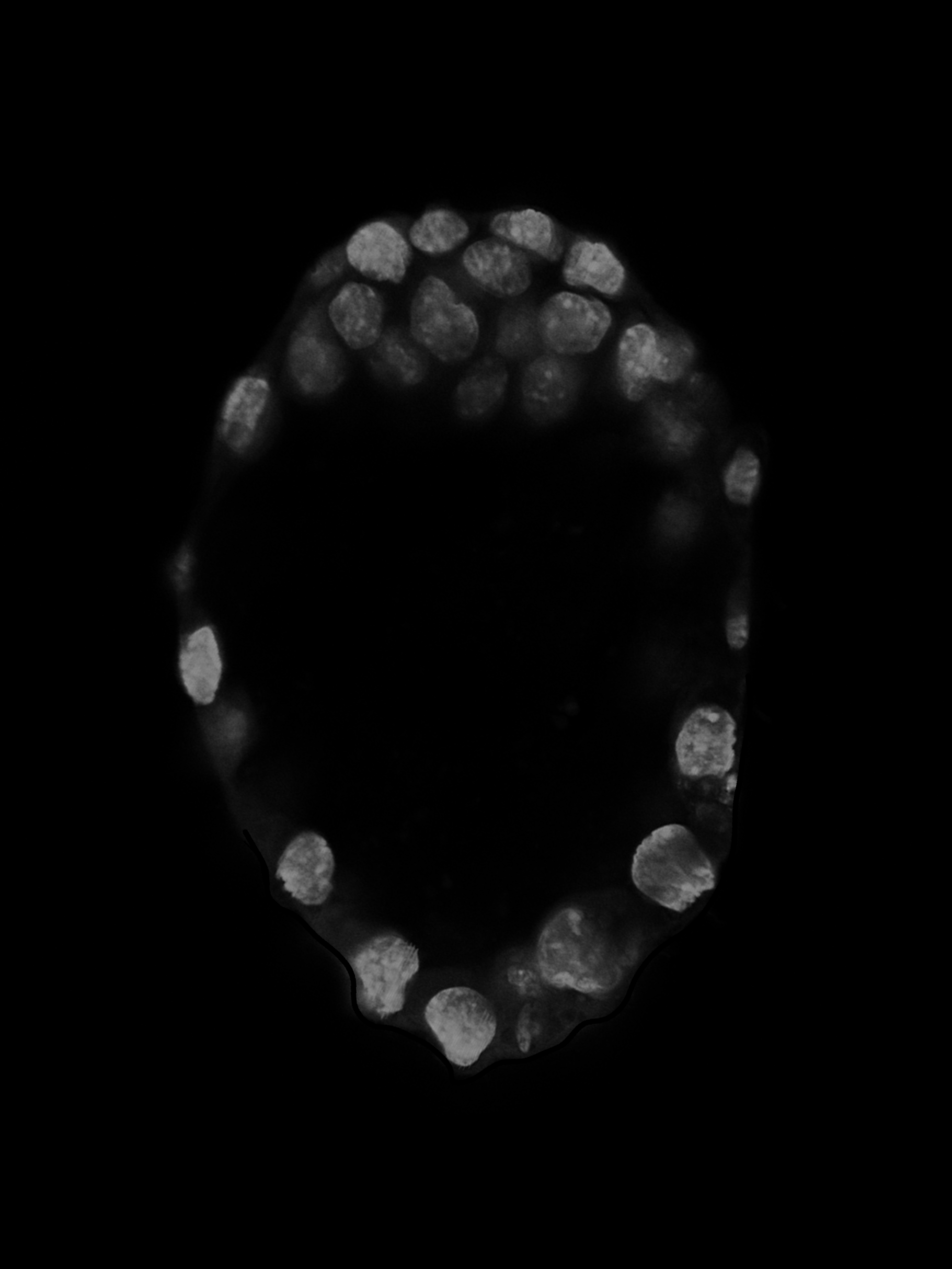

Supplement: Supplementary file 16 — Source Data for Figure 3 [file EMBJ-42-e113280-s017.zip › Figure 3/Panel e/E4.5_in_utero.tif]

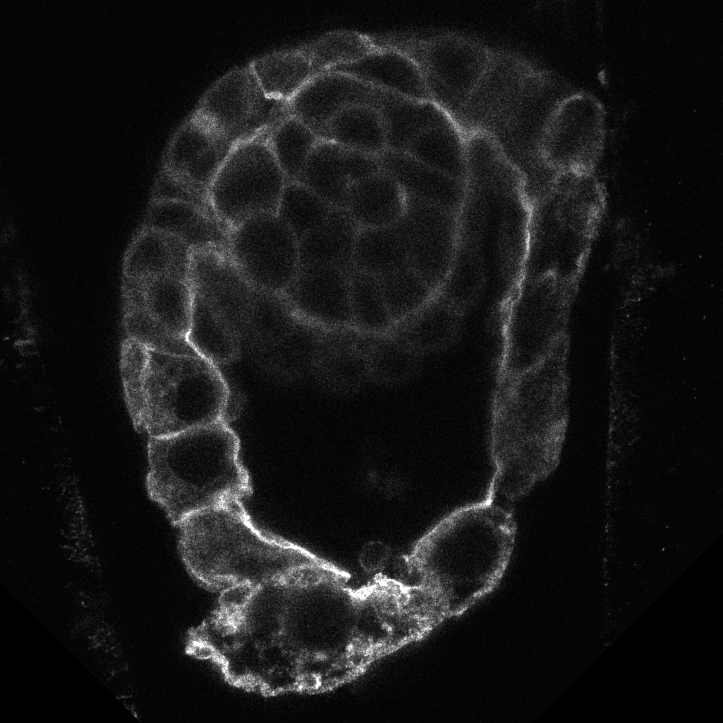

Supplement: Supplementary file 16 — Source Data for Figure 3 [file EMBJ-42-e113280-s017.zip › Figure 3/Panel d/D2_3E-uterus.tif]

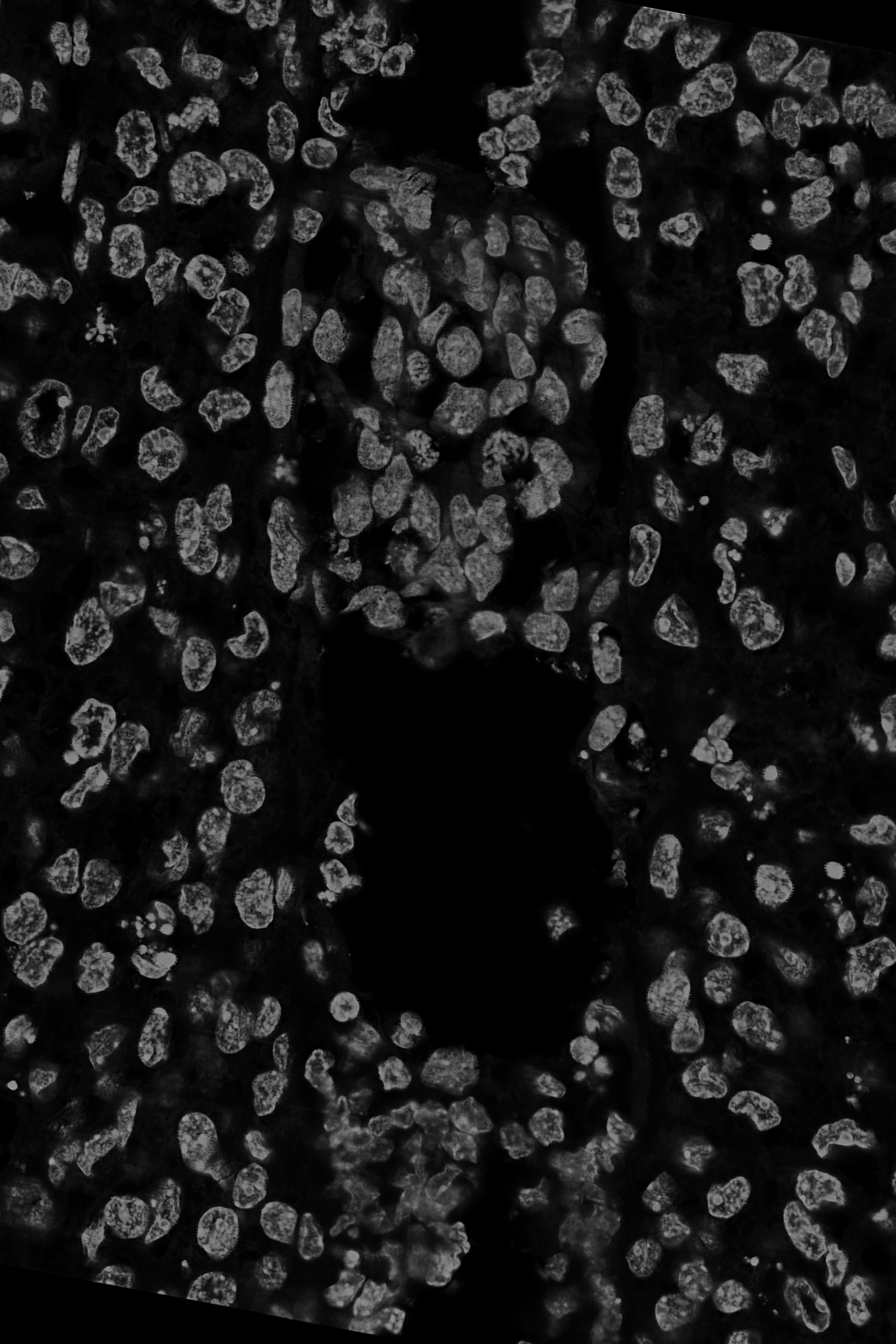

Supplement: Supplementary file 16 — Source Data for Figure 3 [file EMBJ-42-e113280-s017.zip › Figure 3/Panel i/E5.0_in_utero.tif]

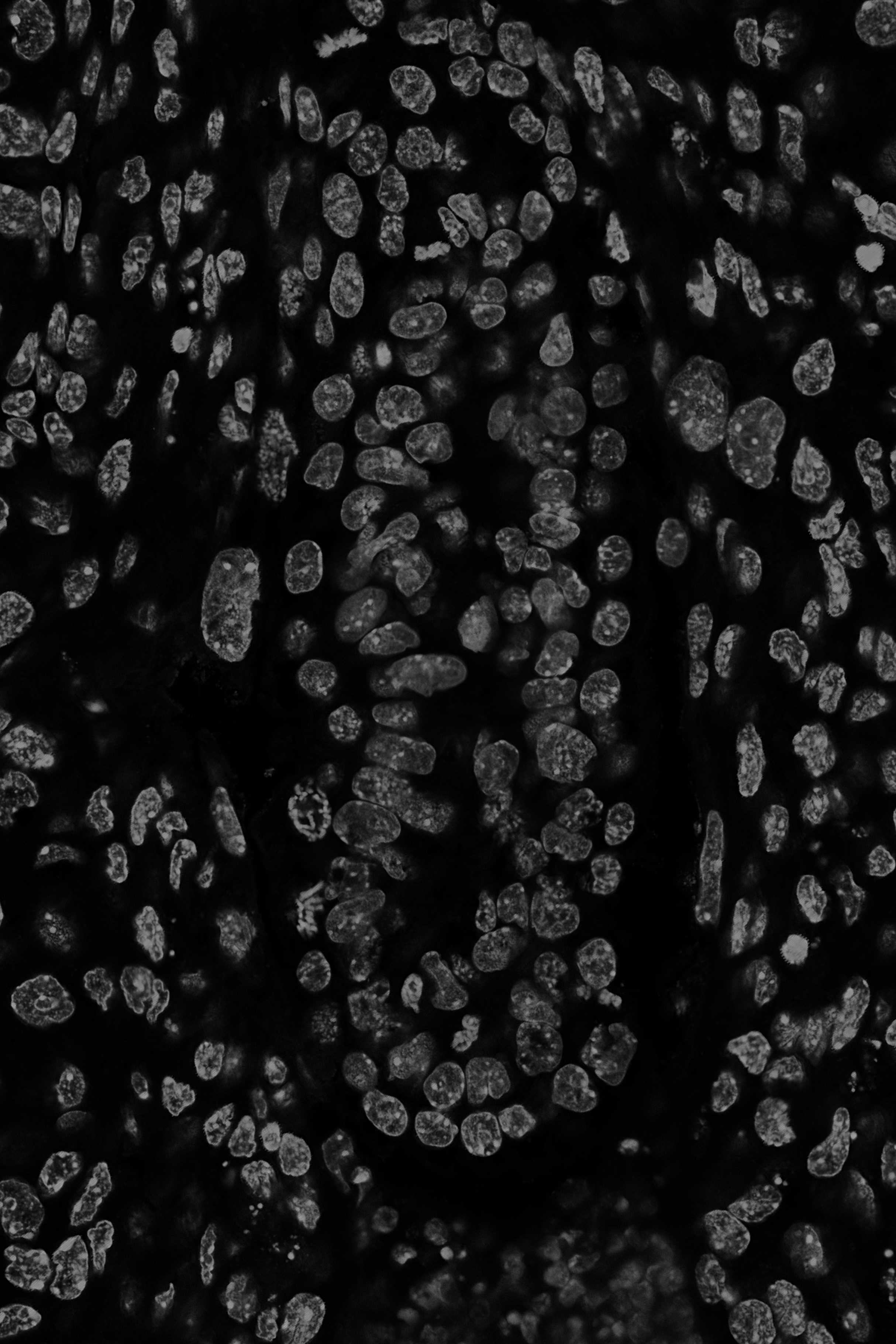

Supplement: Supplementary file 16 — Source Data for Figure 3 [file EMBJ-42-e113280-s017.zip › Figure 3/Panel i/E5.25_in_utero.tif]

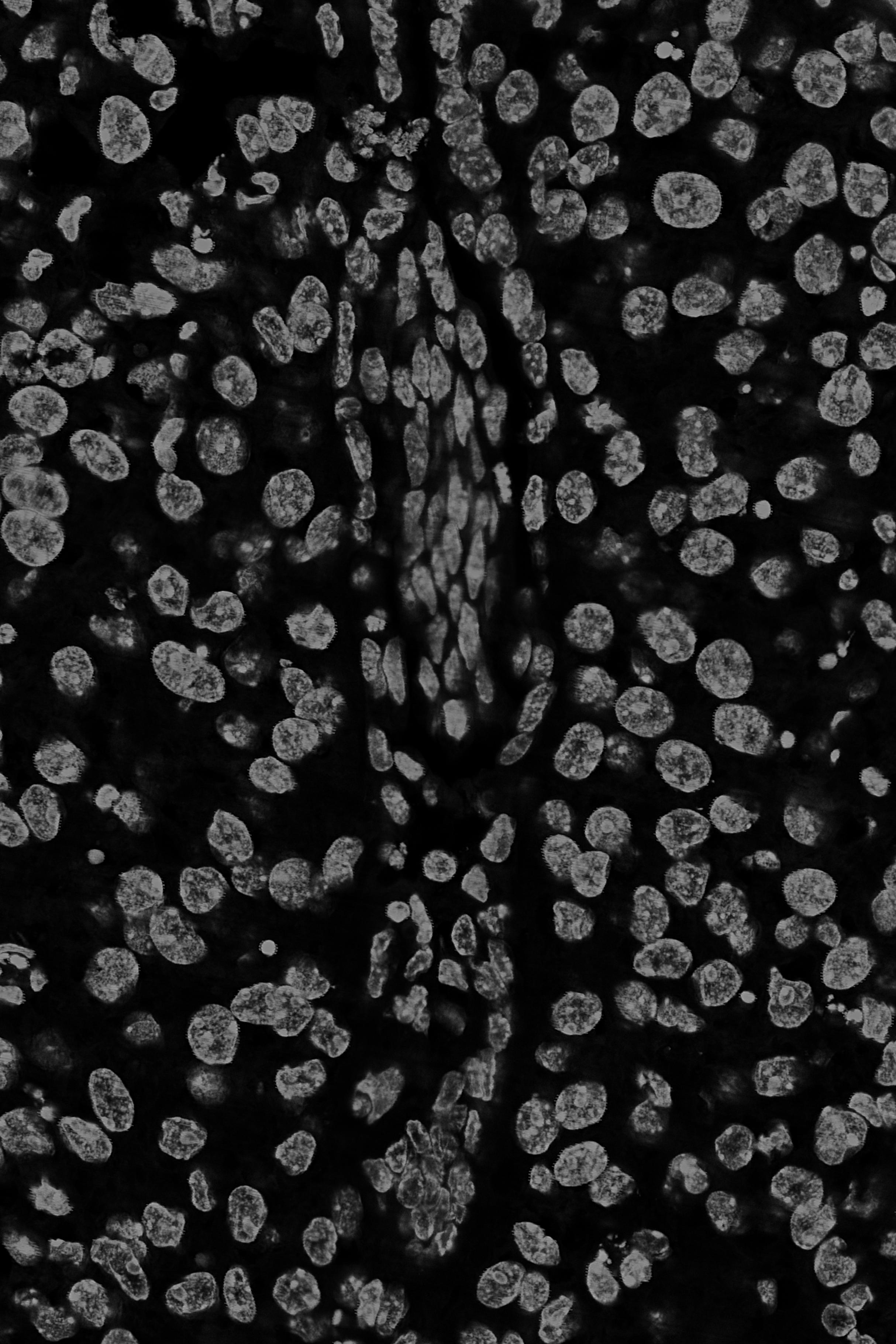

Supplement: Supplementary file 16 — Source Data for Figure 3 [file EMBJ-42-e113280-s017.zip › Figure 3/Panel i/E4.75_in_utero.tif]

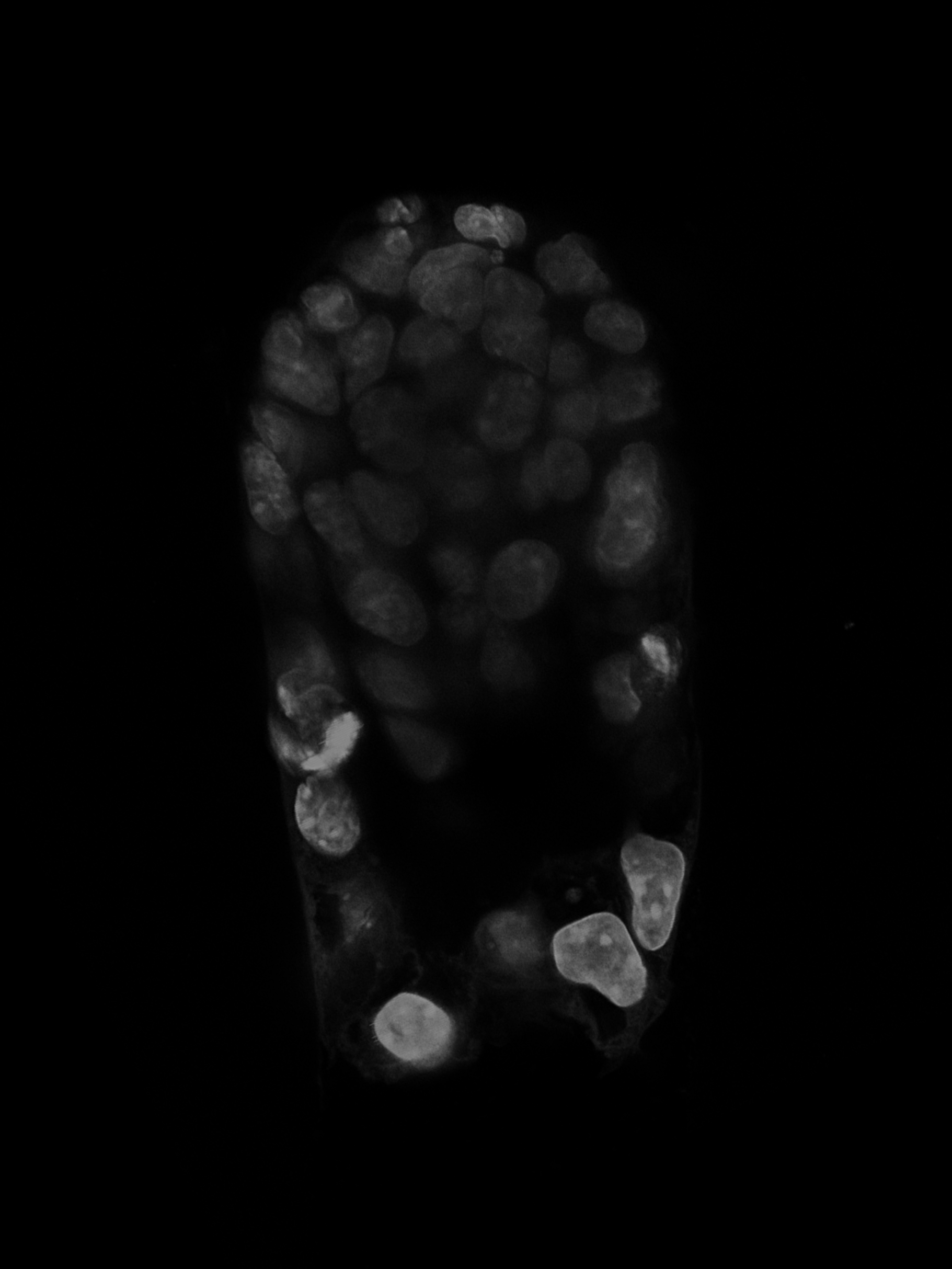

Supplement: Supplementary file 16 — Source Data for Figure 3 [file EMBJ-42-e113280-s017.zip › Figure 3/Panel g/D2_3E-uterus.tif]

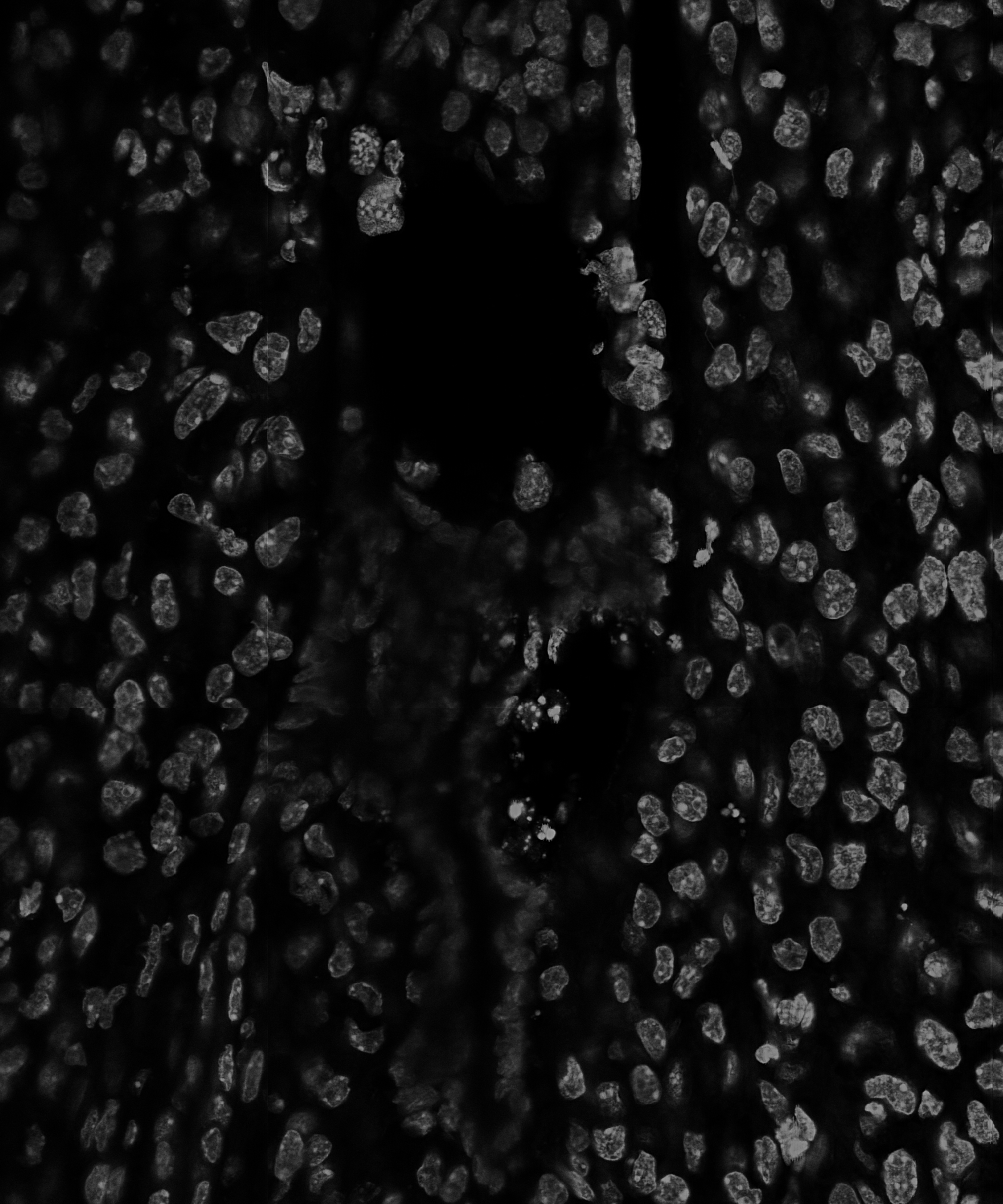

Supplement: Supplementary file 17 — Source Data for Figure 4 [file EMBJ-42-e113280-s009.zip › Figure 4/Panel d/E4.75_in_utero.tif]

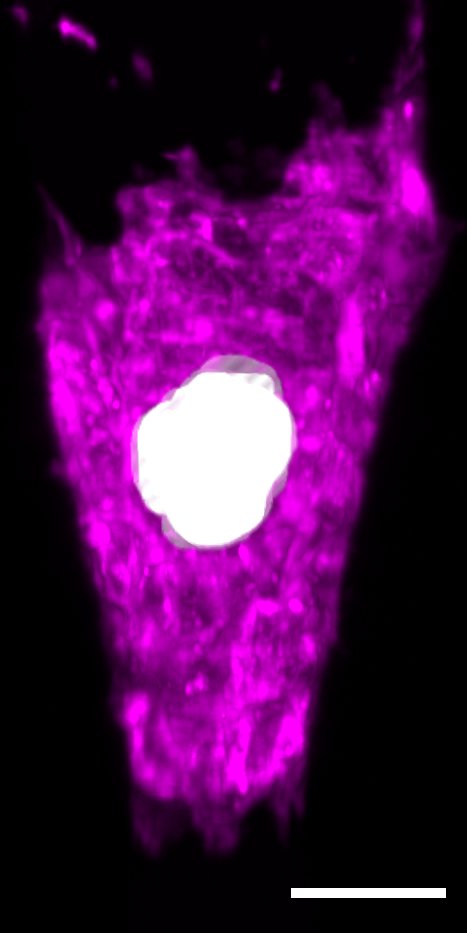

Supplement: Supplementary file 18 — Source Data for Figure 5 [file EMBJ-42-e113280-s014.zip › Figure 5/Panel f/ZMAX_t56.jpg]

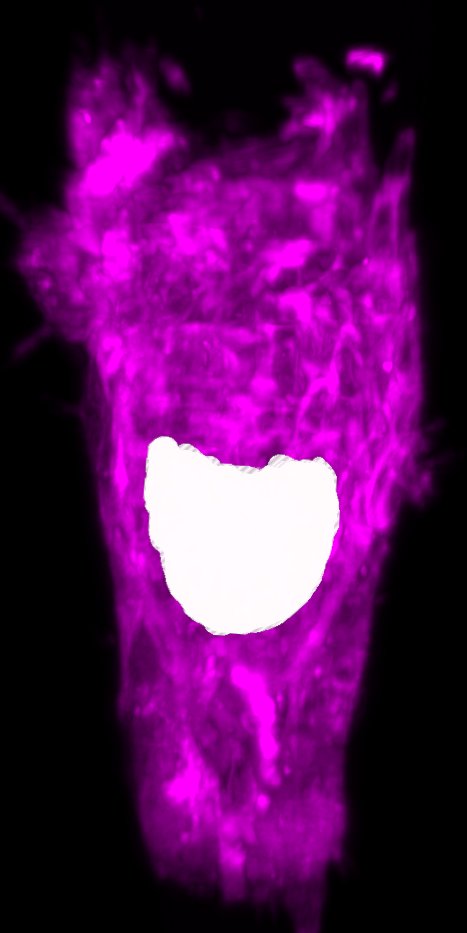

Supplement: Supplementary file 18 — Source Data for Figure 5 [file EMBJ-42-e113280-s014.zip › Figure 5/Panel f/ZMAX_t64.jpg]

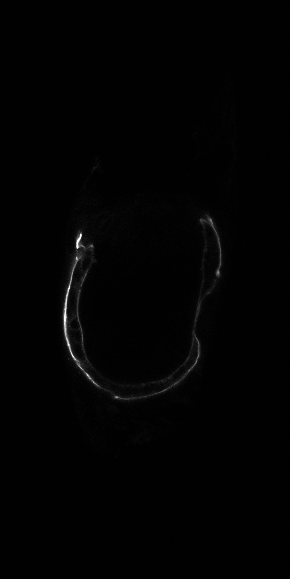

Supplement: Supplementary file 19 — Source Data for Figure 6 [file EMBJ-42-e113280-s015.zip › Figure 6/Panel f/D3_3E-uterus_Rac1 het.tif]

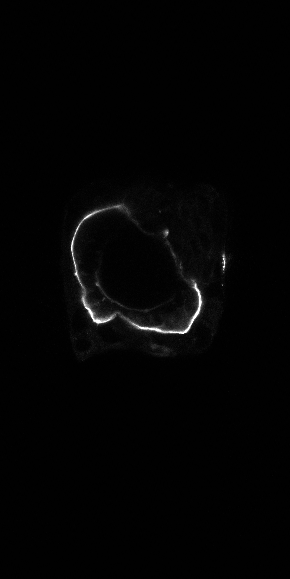

Supplement: Supplementary file 19 — Source Data for Figure 6 [file EMBJ-42-e113280-s015.zip › Figure 6/Panel f/D3_3E-uterus_Rac1 del.tif]

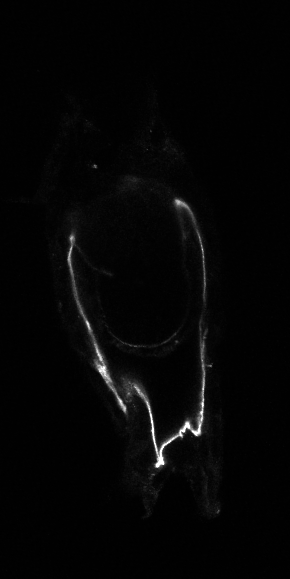

Supplement: Supplementary file 19 — Source Data for Figure 6 [file EMBJ-42-e113280-s015.zip › Figure 6/Panel f/D3_3E-uterus_Rac1 WT.tif]
