# Supplementary material for: Embryo‐uterine interaction coordinates mouse embryogenesis during implantation
Source: EMBO J. 2023 Jul 31;42(17):e113280. doi: 10.15252/embj.2022113280 (PMC10476174; doi:10.15252/embj.2022113280)
Supplement: Supplementary file 20 [file EMBJ-42-e113280-s006.pdf]

THE EMBO JOURNAL

EMBRYO-UTERINE INTERACTION COORDINATES MOUSE EMBRYOGENESIS DURING IMPLANTATION

Vladyslav Bondarenko, Mike Nikolaev, Dimitri Kromm, Roman Belousov, Adrian Wolny, Marloes Blotenburg, Peter Zeller, Saba Rezakhani, Johannes Hugger, Virginie Uhlmann, Lars Hufnagel, Anna Kreshuk, Jan Ellenberg, Alexander van Oudenaarden, Anna Erzberger, Matthias Lutolf, and Takashi Hiiragi
DOI: 10.15252/embj.2022113280

Corresponding author(s): Takashi Hiiragi (t.hiiragi@hubrecht.eu) , Matthias Lutolf (matthias.lutolf@epfl.ch), Anna Erzberger (anna.erzberger@embl.de)

Review Timeline:

Transfer from Review Commons:	13th Dec 22
Editorial Decision:	16th Dec 22
Revision Received:	2nd May 23
Editorial Decision:	6th Jun 23
Revision Received:	16th Jun 23
Accepted:	27th Jun 23

Review COMMONS

Editor: Ieva Gailite

Transaction Report: This manuscript was transferred to The EMBO JOURNAL following peer review at Review Commons.

Review #1

1. Evidence, reproducibility and clarity:

Evidence, reproducibility and clarity (Required)

Summary:

To recapitulate mouse peri-implantation development ex vivo, the authors engineered a uterus-like microenvironment by fabrication of topographically patterned hydrogels and identified the roles of the physical interaction between embryo and hydrogels for egg cylinder formation. Notably, integrin-mediated adhesion between trophoblast and matrix facilitates egg-cylinder shape. Moreover, Live-imaging with light-sheet microscopy led them to propose the hypothesis that the interaction between embryos and hydrogels appeared to be described by a droplet wetting process.

Major comments:

Although the authors claim that the interaction between the uterus and embryo is crucial for egg-cylinder formation, they did not utilize uterus-derived cells nor analyze these. They just observed how blastocysts grow autonomously into the egg-cylinder shape in the hydrogel which has solely physical properties of the uterus but not biochemical features except for the RGD peptide-mediated cell adhesion process. Thus, it is still uncertain if similar mechanisms contribute to egg-cylinder formation in utero. To fulfill the gap between ex vivo morphogenesis and in utero, the authors would be expected to analyze the interaction between trophoblast and uterus in utero if uterine mechanisms can follow the integrin-mediated adhesion and a droplet wetting process. For example, whether integrin can contribute to egg-cylinder formation in utero can be proved by analyzing knock-out phenotypes of integrin-related genes. It will take around six months to conduct such suggested experiments. Otherwise, the authors should modify their statement "the interaction between embryo and uterine" into "the interaction between embryos and uterine-like hydrogels" throughout the manuscript.

Specific point:

Supplemental figure 1h, page 4 lines 20-23:
The authors claim that 1.5-2 % PEG generated the shear modulus at 100-300 Pa, which is in the stiffness range of the E5.5 mouse decidua (Govindasamy et al., 2021). In Govindasamy's paper, elasticity measurements were performed in Petri dishes

using an MFP-3D Classic AFM (Asylum Research, Wiesbaden, Germany) and cantilevers with a force constant of 0.08 with spherical tips (2 mm; NanoWorld, Neuchatel, Switzerland). In the present paper, the shear modulus (G′) of hydrogels was determined by performing small-strain oscillatory shear measurements on a Bohlin CVO 120 rheometer with plate-plate geometry. Therefore, it is not appropriate that Govindasamy's modulus is compared to the authors' modulus directly. For a direct comparison of the two modulus values, the authors can measure the stiffness of PEG with AFM or the shear modulus of the E5.5 mouse decidua by their rheometer.

2. Significance:

Significance (Required)

Strong points:

As described in summary, the authors have newly identified that integrin-mediated adhesion between trophoblast and matrix facilitates egg-cylinder shape and the interaction between embryos and hydrogels is followed by a droplet-wetting process. These findings are considered to be novel by their excellent ex vivo imaging method.

Limitations:

It remains unaddressed that ex vivo mechanisms they have identified contribute to the egg-cylinder formation in utero.

Audience:

The results demonstrated here by the authors are fascinating in terms of general interest after the above concerns are appropriately addressed.

Since I am an experimental biologist, I don't have sufficient expertise to assess if the physical droplet model can recapitulate the interaction of the embryo and pre-patterned hydrogel for egg cylinder formation in detail.

3. How much time do you estimate the authors will need to complete the suggested revisions:

Estimated time to Complete Revisions (Required)

(Decision Recommendation)

Between 3 and 6 months

4. *Review Commons* values the work of reviewers and encourages them to get credit for their work. Select 'Yes' below to register your reviewing activity at Web of Science Reviewer Recognition Service (formerly Publons); note that the content of your review will not be visible on Web of Science.

Web of Science Reviewer Recognition

Yes

Review #2

1. Evidence, reproducibility and clarity:

Evidence, reproducibility and clarity (Required)

The authors developed a new method of ex vivo culture of mammalian embryos. This engineered uterus recapitulates some features of peri-implantation development of the mouse embryo. The authors show that integrin adhesion to the uterine wall through integrin beta 1 is required for proper peri-implantation. They also demonstrate collective migration of the trophoblast on the synthetic hydrogel surface. The authors interpret their results through the physics of wetting, which allows them to conclude that a release of tension enables shape changes in the embryo. Finally, light sheet imaging allows the authors to visualize the interplay between growth and collective motion.

Major

1. The article will be of potential interest to a broader community than mammalian embryo peri-implantation researchers. This broader community will likely not be familiar with the structure and nomenclature of the embryo and surrounding tissues. The introduction of terms in the second paragraph of the introduction should be paralleled by a comprehensive image in Fig. 1. This image should clarify what is considered apical and basal in this context. Similarly, when the model is introduced a more comprehensive scheme should also be provided.

2. The failure of 2D hydrogels to support mouse blastocysts through peri-implantation (Supp Fig. 1) is insufficiently described. Some panels in this figure and not mentioned in the main text. This discussion should be expanded, especially considering that 2D approaches has been quite successful. A detailed discussion of the authors' cylinder approach compared to the best 2D systems published should be provided (Govindasamy et al, for example).
3. Is the hydrogel purely elastic or viscoelastic? The mechanical properties of the hydrogel (viscoelasticity and degradability) should be presented in the main text.
4. The authors call the finding that integrins are required for peri-implantation "striking", but a role for integrins in this process is are already known (see Sutherland et al, for example). The novelty of the authors findings in this regard should be better presented.
5. Figures 2ef (in utero) and 2gh (3E-uterus) show rather different results. In uterus, pERM and ZO1 look quite compartmentalized in the outer region. This is not the case in 3E-uterus shown in Figure 2gh. These data do not seem to support an agreement between in utero and 3E-uterus as mentioned in the text.
6. The authors claim that mTE cells lose cell polarity upon adhesion to the uterine matrix and acquire mesenchymal properties. This claim should be clarified. Cells protrude and become migratory but invasion seems to be collective, suggestive that epithelial features such as cadherin adhesion remain. Are these cells mesenchymal or are they simply epithelial cells with motile capacity (as in wound healing, for example)? How do mesenchymal vs epithelial features compare between in utero and 3E-uterus?
7. If I understand correctly, the model assumes that tension of the droplet-medium interface and is the same in the upper and lower sides of the embryo. However, the mechanical and geometrical properties of cells in both sides are quite different. Is the assumption of same tension justified? Can these tensions be measured or inferred to test this assumption?
8. Along similar lines, attributing a surface tension to a system that is thick (ie several cell layers) and that undergoes apical constriction (ie a bending modulus) is an oversimplification that should be justified. Cells in the pTE change (potentially) their apical, basal and lateral tension during apical constriction. How do these three components relate to what the model simply refers as tension? Additionally, how does the presence of a bending moment alter the wetting picture?
9. The physics of wetting were recently generalized to include additional terms attributed to active components (main associated with polarity, see works by Alert and Casademunt). These active components are not explicitly taken into account in the authors' model. Are they not needed? A brief discussion of this aspect should be provided.
10. In utero, the cavity in which the embryo is implanted is created during implantation. In this situation, the analogy with wetting seems harder to establish

because the embryo spreads as it forms the cavity. How does this alter the authors interpretation?
11. The first paragraph of the supplementary note refers to Fig. 4D. This reference here does not seem correct.

Referees cross-commenting

The three of us coincide in appreciating the novelty and potential impact of the new method.

There is an agreement between all 3 referees to request additional evidence of how well the 3E-uterus captures the in vivo phenomenon. I believe the suggestions provided by my two colleagues in this regard are on point and seem feasible for the applicants within a 6 month period.

I also agree that tension measurements with laser ablation (or other inference techniques) would provide stronger support to the model.

2. Significance:

Significance (Required)

This article provides an important technical advance to study peri-implantation of the mammalian embryo beyond current methods based on 2D substrates. This work will be of interest to the community of early mammalian embryogenesis but also to the broader field of engineering multicellular systems.

As list above, main limitations concern 1) The extent to which their method properly captures peri-implantation, 2) The novelty of some of the authors observations, 3) The soundness of the theoretical model.

My expertise is in experimental biophysics of multicellular systems.

3. How much time do you estimate the authors will need to complete the suggested revisions:

Estimated time to Complete Revisions (Required)

(Decision Recommendation)

Between 1 and 3 months

4. *Review Commons* values the work of reviewers and encourages them to get credit for their work. Select 'Yes' below to register your reviewing activity at [Web of Science Reviewer Recognition Service] (formerly Publons); note that the content of your review will not be visible on Web of Science.

Web of Science Reviewer Recognition

No

Review #3

1. Evidence, reproducibility and clarity:

Evidence, reproducibility and clarity (Required)

Summary:

Provide a short summary of the findings and key conclusions (including methodology and model system(s) where appropriate).
In this paper the authors develop an engineered uterus-like microenvironment to recapitulate peri-implantation development of the whole mouse embryo ex vivo. This new model (3E-uterus) is used for mechanistic studies of embryo implantation. They hint that integrin-mediated adhesion of the embryo to the uterine wall is required for peri-implantation mouse development. The authors use this model also to study the role of tension for embryo development. They postulate that release of tension from the polar side of the embryo upon implantation allows for extra embryonic development. By using mathematical modeling of the implanting embryo as a wetting droplet, the authors link the embryo shape dynamics to the underlying changes in trophoblast adhesion and suggests that the adhesion-mediated tension release facilitates egg cylinder formation. Finally, the authors uncover the role of coordination between trophoblast motility and embryo growth, where trophoblast mobility displaces the Reichart's membrane giving the embryo space to grow. In summary, the authors technically advance the field of developmental biology by providing a model to study peri implantation morphogenesis of the mouse embryo.

Major comments:

- Are the key conclusions convincing?

The key conclusions the authors derive from their experiments are somewhat convincing. Suggested experiments below will strengthen their claims.
- Would additional experiments be essential to support the claims of the paper? Request additional experiments only where necessary for the paper as it is, and do not ask authors to open new lines of experimentation.

Claim 1: The 3E-uterus is representative of mouse embryo peri-implantation.
To claim this a more extensive validation of the embryos cultured in their 3E uterus both via scRNA seq and IF for pluripotency, visceral and parietal endoderm markers is required.
It is also interesting that embryos cultured in the 3E-uterus lose the correct timing of development. Could the authors please comment on this? scRNA seq of the embryos cultured in their system at different timepoint (i.e. Day 1-3) compared to control pre, peri and post implantation embryos could help answer this question.

Claim 2: The release of tension from the polar side upon implantation allows for extra embryonic development.
To quantitatively measure the difference in tension before and after implantation is technically very challenging. However, this paper could benefit of further validations including IF stainings for markers such as E-cadherin, F-actin and Phospho myosin. In addition to this, treatments with Y27 and blebbistatin of the embryo would allow to further study the role of cell tension on embryo implantation. Finally, a laser ablation experiment at the cell junctions of the polar region before and after implantation would help to answer this question but this could be technically challenging due to the curvature nature of embryos.

Claim 3: Integrin-mediated adhesion between the trophoblast and the uterine matrix is required for in utero-like transition of the blastocyst to the egg cylinder.
In Figure 2a the authors show that embryos cultured in 3E-uterus without RGD do not develop and hypothesize this is due to lack of integrin binding. A control experiment using a non-integrin binding peptide is beneficial here.

Claim 4: The spatial orientation of the embryo plays a key role in mouse peri implantation development.
In Figure 5i-j, the authors place embryos in a downward (i) and upward (j) orientation. Could the authors also please comment on whether they believe the orientation, the way the embryo feels the gravity plays a role in implantation?
Is the amount of space that the embryo has to grow in the limiting factor on

development? Could the authors use 3E-uterus models with different lengths (by using molds with different spacing) to see the role of geometry and space that the embryo has for trophoblast mobility and embryo growth. What would happen if the embryo were very close to the bottom of the hydrogel?

Claim 5: A mathematical model based on the wetting droplet recapitulates the embryo in their system.
Could the authors comment on whether their mathematical model considers proliferation, and would proliferation have an impact on the system's kinetics? What is the role of polar TE proliferation and how does that influence the trophectoderm morphology? If the embryo is geometrically confined, can the authors exclude that this confinement is influencing cell shape?
- Should the authors qualify some of their claims as preliminary or speculative, or remove them altogether?

In this technical advance paper, the claims will become more robust with the suggested experiments above. Claims of implantation should be changed to accurately reflect that the 3E-uterus models peri implantation as there is no invasion in the 3D hydrogel matrix. In addition to this, the uterine cells are missing which are required to fully recapitulate the mechanisms of embryo peri-implantation.
- Are the suggested experiments realistic in terms of time and resources? It would help if you could add an estimated cost and time investment for substantial experiments.

3- 6 months will allow the authors to address all questions above. Yet, the laser ablation experiment might be difficult to perform due to the curvature of the embryo.
- Are the data and the methods presented in such a way that they can be reproduced?

We appreciate the details of the materials and methods section particularly of the imaging.
- Are the experiments adequately replicated and statistical analysis adequate?

Yes

Minor comments:

- Specific experimental issues that are easily addressable.

1. Laminin staining in Figure 2A is only done in in vitro embryos but not in in vivo embryos. Could the authors add the missing staining?
2. It would be beneficial to have both active and normal integrin stainings in E4.5

embryos.
3. Could the authors provide stainings for mesenchymal markers for E4.5 and D2 3E uterus?
4. Can the authors comment on Figure Supp. 3D where the timing seems to be flipped?
5. Why was 600 um chosen for the depth of the 3E-uterus?
- Are prior studies referenced appropriately?

How do the findings in this paper relate to the findings in Weberling et al (PMID 33472064), where they show that in vivo, the polar trophectoderm exerts physical force upon the epiblast, causing it to transform from an oval into a cup shape?
- Are the text and figures clear and accurate?

Overall the text and figures are clear and accurate.
In figure 2E and 2G, the outline covers the staining. Would it be possible to have it without the outline in the supplementary?
- Do you have suggestions that would help the authors improve the presentation of their data and conclusions?
It would be very informative to have for each panel in which a representative image is used for that image to be marked into the quantified data (graphs).

Overall, the manuscript is very well written and the conclusions are informative and clear.

2. Significance:

Significance (Required)

- Describe the nature and significance of the advance (e.g. conceptual, technical, clinical) for the field.

The advance presented in this paper is technical. In this methodology paper, the authors use their novel model to investigate the mechanics of embryo peri-implantation and hint at new conceptual findings such as the role of wetting properties of the embryo onto the ECM of the uterus.
- Place the work in the context of the existing literature (provide references, where appropriate).

Since embryos become hidden in the womb upon implantation, ex vivo cultures provide an experimental setting to monitor, measure and manipulate embryonic development. Ex vivo culture of peri-implantation (mouse) embryos so far relied on

embryonic growth on 2D plastic surfaces (PMID: 4930085, 4562729 and 24529478) or 3D bioreactors (PMID: 33731940). Although important, these assays do not recapitulate the interaction with the uterine cells and ECM (the in vivo scenario). In this study initial steps are taken to recapitulate the interaction of the embryo with the uterine ECM during peri-implantation. Uterine cells are however missing from this new system, which is important for understanding the full mechanism of implantation.
- State what audience might be interested in and influenced by the reported findings.

Developmental biologists
- Define your field of expertise with a few keywords to help the authors contextualize your point of view. Indicate if there are any parts of the paper that you do not have sufficient expertise to evaluate.

Bioengineer, bioinformatician and developmental biologists working with embryo models and hydrogels. There is not sufficient expertise to evaluate the mathematical modeling.

3. How much time do you estimate the authors will need to complete the suggested revisions:

Estimated time to Complete Revisions (Required)

(Decision Recommendation)

Between 3 and 6 months

4. *Review Commons* values the work of reviewers and encourages them to get credit for their work. Select 'Yes' below to register your reviewing activity at [Web of Science Reviewer Recognition Service]() (formerly Publons); note that the content of your review will not be visible on Web of Science.

Web of Science Reviewer Recognition

No

Revision Plan

Manuscript number: RC-2022-01701
Corresponding author(s): Takashi Hiiragi

[The "revision plan" should delineate the revisions that authors intend to carry out in response to the points raised by the referees. It also provides the authors with the opportunity to explain their view of the paper and of the referee reports.

The document is important for the editors of affiliate journals when they make a first decision on the transferred manuscript. It will also be useful to readers of the reprint and help them to obtain a balanced view of the paper.

If you wish to submit a full revision, please use our "Full Revision" template. ***It is important to use the appropriate template to clearly inform the editors of your intentions.****]*

1. General Statements

We greatly thank all three referees for constructive comments for our manuscript, appreciating the technical advances of our study, excellent live imaging, data quality, and overall soundness of our highly interdisciplinary work. The substantial challenges and limitations for the hypothesis-driven study of embryo implantation in utero motivated us to undertake a bottom-up approach, where the complexity of the system can be decomposed to a finite number of controllable – in this case, biomechanical - parameters. The present study marks the very beginning of mechanistic understanding of a complex interaction between the embryo and the maternal uterine environment using ex vivo engineering, live imaging, and biophysical modeling. As was pointed out by the referees, we envision that the next generations of methods will increase the complexity to unleash new aspects of embryo-maternal interactions and feedback mechanisms across the scales of this fascinating biological system.

This following description incorporates our answers to the reviewers' comments, the revisions that we've already carried out to address several major points, and the remaining revisions we will perform during the next 2 months.

2. Description of the planned revisions

Insert here a point-by-point reply that explains what revisions, additional experimentations and analyses are planned to address the points raised by the referees.

Reviewer #1 (Evidence, reproducibility and clarity (Required)):
Summary:
To recapitulate mouse peri-implantation development ex vivo, the authors engineered a uterus-like microenvironment by fabrication of topographically patterned hydrogels and identified the roles of the physical interaction between embryo and hydrogels for egg cylinder formation. Notably, integrin-mediated adhesion between trophoblast and matrix facilitates egg-cylinder shape. Moreover, Live-imaging with light-sheet microscopy led them to propose the hypothesis that the interaction between embryos and hydrogels appeared to be described by a droplet wetting process.

Major comments:
Although the authors claim that the interaction between the uterus and embryo is crucial for egg-cylinder formation, they did not utilize uterus-derived cells nor analyze these. They just observed how blastocysts grow autonomously into the egg-cylinder shape in the hydrogel which has solely physical properties of the uterus but not biochemical features except for the RGD peptide-mediated cell adhesion process. Thus, it is still uncertain if similar mechanisms contribute to egg-cylinder formation in utero. To fulfill the gap between ex vivo morphogenesis and in utero, the authors would be expected to analyze the interaction between trophoblast and uterus in utero if uterine mechanisms can follow the integrin-mediated adhesion and a droplet wetting process. For example, whether integrin can contribute to egg-cylinder formation in utero can be proved by analyzing knock-out phenotypes of integrin-related genes. It will take around six months to conduct such suggested experiments. Otherwise, the authors should modify their statement "the interaction between embryo and uterine" into "the interaction between embryos and uterine-like hydrogels" throughout the manuscript.

This is an important point raised by several reviewers. To address the *in vivo* relevance of our findings of the involvement of a wetting-like integrin-mediated interaction with the uterine environment in the embryo morphogenesis, we will analyze the ITGB1 knockout embryos (mouse line available in the laboratory) for their implantation phenotype and morphology *in utero* at the onset of implantation using tissue sectioning and immunofluorescence. The embryo will be differentially labeled from the surrounding maternal tissues with an endogenously expressed fluorescent membrane protein provided by a double-transgenic male.

Reviewer #2 (Evidence, reproducibility and clarity (Required)):

The authors developed a new method of ex vivo culture of mammalian embryos. This engineered uterus recapitulates some features of peri-implantation development of the mouse embryo. The authors show that integrin adhesion to the uterine wall through integrin beta 1 is required for proper peri-implantation. They also demonstrate collective migration of the trophoblast on the synthetic hydrogel surface. The authors interpret their results through the physics of wetting, which allows them to conclude that a release of tension enables shape changes in the embryo. Finally, light sheet imaging allows the authors to visualize the interplay between growth and collective motion.

Revision Plan

Major

1) The article will be of potential interest to a broader community than mammalian embryo peri-implantation researchers. This broader community will likely not be familiar with the structure and nomenclature of the embryo and surrounding tissues. The introduction of terms in the second paragraph of the introduction should be paralleled by a comprehensive image in Fig. 1. This image should clarify what is considered apical and basal in this context. Similarly, when the model is introduced a more comprehensive scheme should also be provided.

We thank the reviewer for this suggestion to improve the clarity of the manuscript for a broader audience. We will include the proposed explanatory schematics in the revised manuscript.

2) The failure of 2D hydrogels to support mouse blastocysts through peri-implantation (Supp Fig. 1) is insufficiently described. Some panels in this figure and not mentioned in the main text. This discussion should be expanded, especially considering that 2D approaches has been quite successful. A detailed discussion of the authors' cylinder approach compared to the best 2D systems published should be provided (Govindasamy et al, for example).

We have corrected the reference to these panels in the main text and will provide a more comprehensive discussion of our approach in comparison to the published methods, such as Govindasamy et al., 2021 and Ichikawa et al., 2022.

Govindasamy et al., 2021, doi: 10.1016/j.devcel.2021.10.014.
Ichikawa et al., 2022, https://doi.org/10.1016/j.devcel.2021.12.023.

4) The authors call the finding that integrins are required for peri-implantation "striking", but a role for integrins in this process is are already known (see Sutherland et al, for example). The novelty of the authors findings in this regard should be better presented.

We thank the reviewer for raising this point. The discovered role of integrin-mediated embryo-uterine interaction in peri-implantation embryo morphogenesis will be emphasized more explicitly in the manuscript text and in a proposed mechanistic model.

7) If I understand correctly, the model assumes that tension of the droplet-medium interface and is the same in the upper and lower sides of the embryo. However, the mechanical and geometrical properties of cells in both sides are quite different. Is the assumption of same tension justified? Can these tensions be measured or inferred to test this assumption?

8) Along similar lines, attributing a surface tension to a system that is thick (ie several cell layers) and that undergoes apical constriction (ie a bending modulus) is an oversimplification that should be justified. Cells in the pTE change (potentially) their apical, basal and lateral tension during apical constriction. How do these three components relate to what the model simply refers as tension? Additionally, how does the presence of a bending moment alter the wetting picture?

9) The physics of wetting were recently generalized to include additional terms attributed to active components (main associated with polarity, see works by Alert and Casademunt). These active components are not explicitly taken into account in the authors' model. Are they not needed? A brief discussion of this aspect should be provided.

Response to points 7 – 9:

Our goal here is to find a mechanical description, which is sufficiently simple to make a quantitative match with the experimental observables. Thereby we also identify the key drivers of embryo shape dynamics during implantation. Indeed, the quantitative agreement between our simple model and the experimentally measured contact angles in both bottom and top parts of the embryo suggests that the predominant driving mechanism underlying embryo shape dynamics in 3E-uterus is—in addition to volume changes—an increase in adhesion between TE cells and substrate. Given that the volume variation is known from experiments, our minimal model is well-constrained and permits the embryo-specific inference of the adhesion dynamics.

To further support the soundness of our model, we performed leave-one-out cross validation, and recovered that we can not only fit, but also predict the measured dynamics within error bounds in almost all cases (Review Figure 1). We will include these results in the manuscript as a supplementary figure.

Figures for referees not shown.

With the new revision we will give a more detailed account of our rationale and biophysical aspects, which the reviewer mentioned above. In particular, as we will explain, extending the model to account for additional effects, e.g. mechanical differences between mural and polar side, the morphogenesis of the ExE, or active migratory polarity of the cells (Perez-Gonzales et al. 2019) would lead to a large number of unconstrained parameters.

In Review Figure 2, we show quantifications of the relative double-phosphorylated myosin levels at the apical cell surfaces of mural and polar TE, to approximate the relative difference in tension. At the onset of adhesive interaction corresponding to day 1 of 3E-uterus, the tensions in both polar and mural

TE are comparable, although on average, mural TE cells have higher ppMyo levels than polar TE (Review Figure 2b, d). Although this result is insufficient to conclude that the surface tensions are equal, we also did not observe a striking difference and, hence, a clear justification for increasing the complexity of the model based on the incorporation of a set of unknown (and potentially non-measurable) parameters.

Figures for referees not shown.

Perez-Gonzales et al. 2019, https://doi.org/10.1038/s41567-018-0279-5.

10) In utero, the cavity in which the embryo is implanted is created during implantation. In this situation, the analogy with wetting seems harder to establish because the embryo spreads as it forms the cavity. How does this alter the authors interpretation?

This is an excellent point. On the basis of our findings here, we might propose that implantation into the uterine wall in vivo can be understood as a soft wetting problem (Andreotti and Snoeijer, 2020), i.e. where a droplet interacts with and deforms a soft substrate. Although addressing the dynamic nature of embryo-uterine interaction is beyond the scope of the current study, we will certainly elaborate our discussion of this interesting aspect.

Andreotti and Snoeijer, 2020, https://doi.org/10.1146/annurev-fluid-010719-060147.

11) The first paragraph of the supplementary note refers to Fig. 4D. This reference here does not seem correct.

We will correct the reference in the supplementary note.

Reviewer #3 (Evidence, reproducibility and clarity (Required)):

Summary:
Provide a short summary of the findings and key conclusions (including methodology and model system(s) where appropriate).
In this paper the authors develop an engineered uterus-like microenvironment to recapitulate peri-implantation development of the whole mouse embryo ex vivo. This new model (3E-uterus) is used for mechanistic studies of embryo implantation. They hint that integrin-mediated adhesion of the embryo to the uterine wall is required for peri-implantation mouse development. The authors use this model also to study the role of tension for embryo development. They postulate that release of tension from the polar side of the embryo upon implantation allows for extra embryonic development. By using mathematical modeling of the implanting embryo as a wetting droplet, the authors link the embryo shape dynamics to the underlying changes in trophoblast adhesion and suggests that the adhesion-mediated tension release facilitates egg cylinder formation. Finally, the authors uncover the role of coordination between trophoblast motility and embryo growth, where trophoblast mobility displaces the Reichart's membrane giving the embryo space to grow. In summary, the authors technically advance the field of developmental biology by providing a model to study peri implantation morphogenesis of the mouse embryo.

Major comments:

- Are the key conclusions convincing?
The key conclusions the authors derive from their experiments are somewhat convincing. Suggested experiments below will strengthen their claims.

- Would additional experiments be essential to support the claims of the paper? Request additional experiments only where necessary for the paper as it is, and do not ask authors to open new lines of experimentation.
Claim 1: The 3E-uterus is representative of mouse embryo peri-implantation.
To claim this a more extensive validation of the embryos cultured in their 3E uterus both via scRNA seq and IF for pluripotency, visceral and parietal endoderm markers is required.

It is also interesting that embryos cultured in the 3E-uterus lose the correct timing of development. Could the authors please comment on this? scRNA seq of the embryos cultured in their system at different timepoint (i.e. Day 1-3) compared to control pre, peri and post implantation embryos could help answer this question.

Indeed, 3E-uterus embryos reached the E4.5 stage in two days instead of one day of *in utero* development. The early developmental delay was also observed in the previous *ex vivo* studies (Bedzhov et al., 2014; Govindasamy et al., 2021), suggesting that additional cues might be required to enable timely adaptation of the trophectoderm to the *ex vivo* environment. Uterine endometrium provides a complex composition of adhesive molecules before the embryo reaches the underlying ECM (Dey et al., 2004). Incorporating cellular components into our platform may facilitate understanding of the role of cell-cell interactions and signaling between the embryo and the uterus. The present methods provide a powerful experimental platform for such mechanistic studies in the future. In perspective, they can help understanding the functional cross-talk between the uterine tissue dynamics, embryo morphology, and size. To additionally validate our embryo culture method, we will perform scRNA-seq or a more extensive IF analysis for pluripotency, visceral and parietal endoderm markers.

Bedzhov et al., 2014, https://doi.org/10.1038/nprot.2014.186
Govindasamy et al, 2021, https://doi.org/0.1016/j.devcel.2021.10.014
Dey et al., 2004, doi: 10.1210/er.2003-0020

Claim 5: A mathematical model based on the wetting droplet recapitulates the embryo in their system.
Could the authors comment on whether their mathematical model considers proliferation, and would proliferation have an impact on the system's kinetics? What is the role of polar TE proliferation and how does that influence the trophectoderm morphology? If the embryo is geometrically confined, can the authors exclude that this confinement is influencing cell shape?

Our model fully accounts for the volume dynamics of the embryo throughout implantation. These are dominated by the inflation-collapse dynamics of the blastocoel (Chan et al., 2019) and not proliferation - the analysis of live imaging data indicated that mural TE cells rarely divide (12% of mTE/TB cell nuclei divided during 24 hours of live imaging; n=158 - but any effect of proliferation is captured with our volume term. We will add a clarification of this point to the manuscript.

Chan et al., 2019, doi: 10.1038/s41586-019-1309-x.

Minor comments:

- Specific experimental issues that are easily addressable.

2. It would be beneficial to have both active and normal integrin stainings in E4.5 embryos.

Such immunostaining will be shown in the Supplementary figure.

3. Could the authors provide stainings for mesenchymal markers for E4.5 and D2 3E uterus?

We will provide such immunostaining for E4.5 and D2 3E-uterus, in addition to D3 3E-uterus (Review Figure 4).

It would be very informative to have for each panel in which a representative image is used for that image to be marked into the quantified data (graphs).

We will modify the relevant figure panels according to this suggestion.

How do the findings in this paper relate to the findings in Weberling et al (PMID 33472064), where they show that in vivo, the polar trophectoderm exerts physical force upon the epiblast, causing it to transform from an oval into a cup shape?

We will discuss in more detail how our findings relate to this study.

3. Description of the revisions that have already been incorporated in the transferred manuscript

Please insert a point-by-point reply describing the revisions that were already carried out and included in the transferred manuscript. If no revisions have been carried out yet, please leave this section empty.

Reviewer #1 (Evidence, reproducibility and clarity (Required)):
Specific point:
Supplemental figure 1h, page 4 lines 20-23:
The authors claim that 1.5-2 % PEG generated the shear modulus at 100-300 Pa, which is in the stiffness range of the E5.5 mouse decidua (Govindasamy et al., 2021).
In Govindasamy's paper, elasticity measurements were performed in Petri dishes using an MFP-3D Classic AFM (Asylum Research, Wiesbaden, Germany) and cantilevers with a force constant of 0.08 with spherical tips (2 mm; NanoWorld, Neuchatel, Switzerland). In the present paper, the shear modulus (G′) of hydrogels was determined by performing small-strain oscillatory shear measurements on a Bohlin CVO 120 rheometer with plate-plate geometry. Therefore, it is not appropriate that Govindasamy's modulus is compared to the authors' modulus directly. For a direct comparison of the two modulus values, the authors can measure the stiffness of PEG with AFM or the shear modulus of the E5.5 mouse decidua by their rheometer.

Reviewer #1 makes a valid point about the differences in methods used for stiffness measurements and questions the appropriateness of a comparison between hydrogel stiffness in our study and mouse decidual stiffness in Govindasamy et al. 2021. The relationship between the shear modulus (G) and elastic modulus (E) of hydrogels are generally considered to be related by the Trouton ratio E = 3G (simplification of E = 2G (1+v), assuming v = 0.5, where v is the Poisson ratio for soft incompressible materials). We note, however, that at lower crosslinking degrees and hence larger mesh sizes and lower stiffnesses, modulus values determined from AFM and bulk rheology data indeed start to diverge, with AFM commonly generating higher moduli compared to bulk rheology. For example, in Abrego et al., 2022, the authors compared elastic and shear moduli of PEG (8-arm Vs with DTT, similar to conventional PEG-PEP gels in terms of network architecture, and softer than LDTM gels) and Fibrin gels, illustrating that low concentrations of PEG (2.5% W/V) led to larger E/G’ ratios (i.e., up to 8.0) and therefore a more non-affine network. As LDTM PEGs contain lower structural defects compared to conventional PEGs (Rezakhani et al., 2020), and therefore have higher elastic modulus at lower PEG content, we cautiously motivated relating the stiffness measurements by using the E = 2G (1+v) relationship, assuming v = 0.5, where v is the Poisson ratio for soft incompressible materials. Figure 2D of Govindasamy et al, 2021 shows the elastic modulus of the decidual tissue as 400 Pa, which can be comparable to the 100-200Pa shear modulus of our LDTM PEG used in the study.

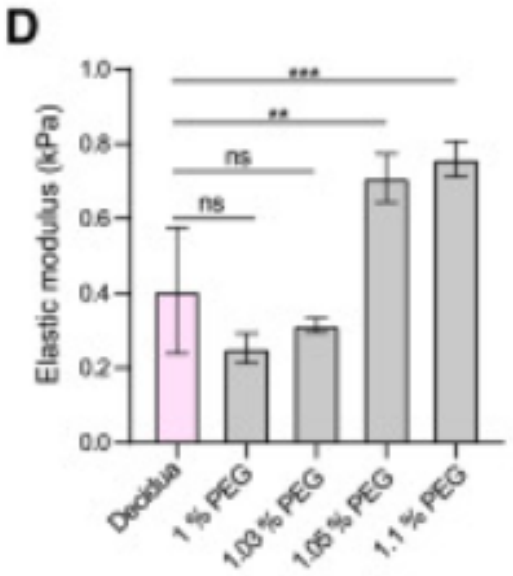

Figure 2D, Govindasamy et al, 2021

Abrego et al., 2022, https://doi.org/10.1002/macp.202100366
Govindasamy et al, 2021, https://doi.org/0.1016/j.devcel.2021.10.014
Rezakhani et al., 2020, https://doi.org/10.1002/adfm.202000761

Reviewer 2

3) Is the hydrogel purely elastic or viscoelastic? The mechanical properties of the hydrogel (viscoelasticity and degradability) should be presented in the main text.

The mechanical properties of the Low-Defect Thiol-Michael (LDTM) PEG, which we used in this study, were thoroughly characterized in our previous work (Rezakhani et al., 2020).

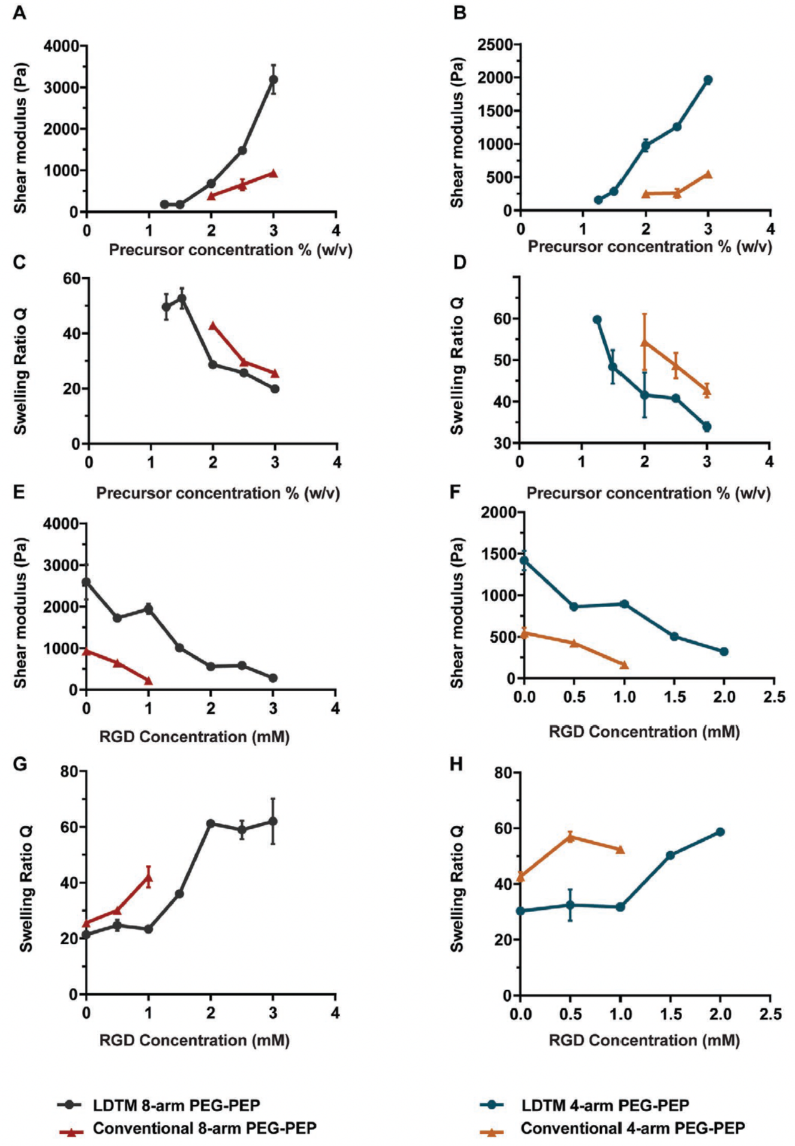

Figure 2. Network characterization. A,B) 4- and 8-arm LDTM hydrogels (no RGD) have a higher shear modulus and C,D) lower swelling ratios compared to conventional PEG-PEP gels. E–H) Shear moduli and swelling ratios of hydrogels formed at 3% (w/v) and at $r = 0.8$ as a function of RGD concentration. All data are reported as mean ± S.D. ($n = 3$).

In the figure above, we characterized the elastic moduli and swelling ratios for different polymer contents and RGD concentrations. The effect of the stoichiometry of functional groups (or a number

of elastically active chains) in conventional and LDTM hydrogels is also evaluated in Supplementary Figures 3 and 4 of the same study.

There, we also characterized the bulk degradation due to the hydrolysis of acrylate groups (Figure 4) by quantifying the elastic moduli and the biological effects on organoid growth.

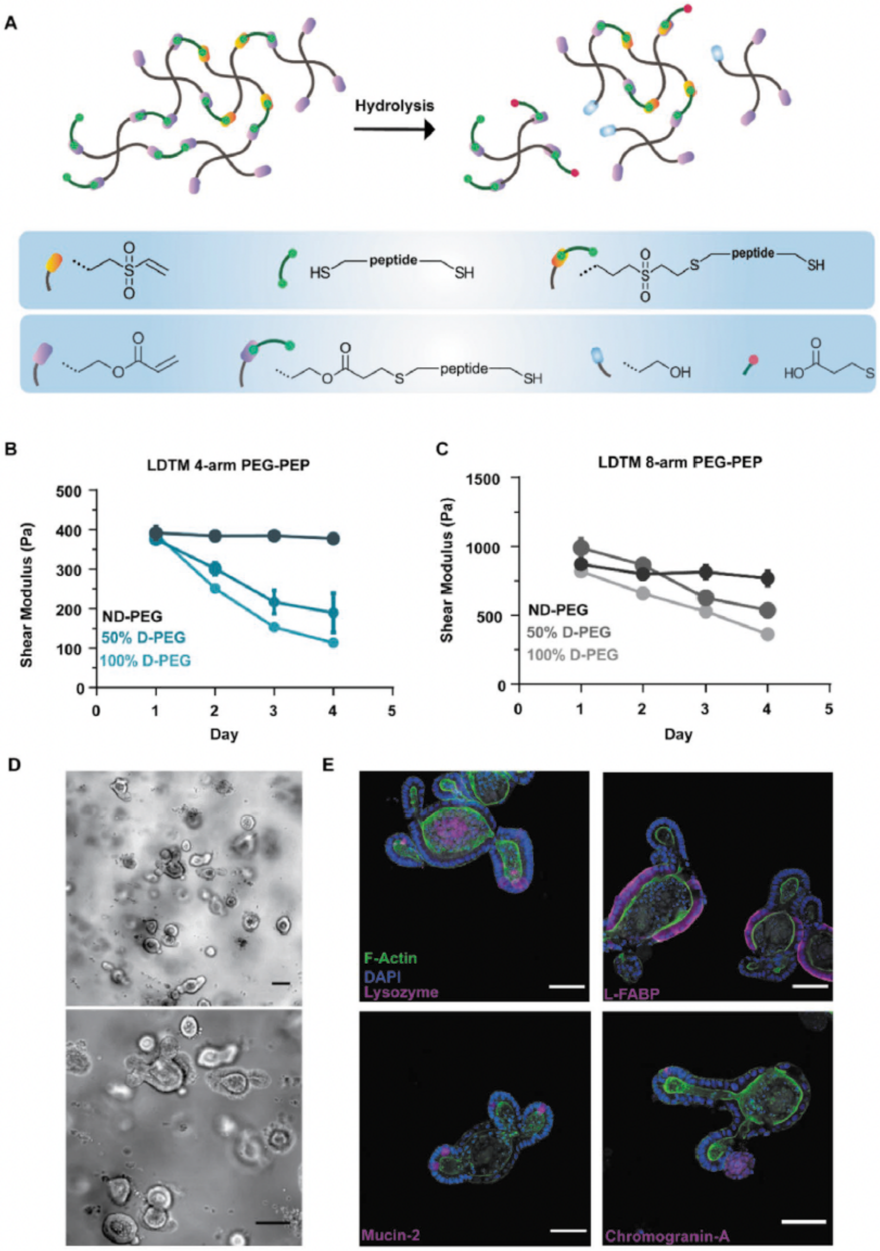

Figure 4. Dynamic matrices for organoid formation in one step. A) Schematic illustrating the ester hydrolysis of LDTM hydrogels containing peptide-functionalized PEG-Acr and PEG-VS macromers. B,C) Softening rate in 4- and 8-arm LDTM hydrogels (2.5%, 1×10^{-3} M RGD) with different ratios of PEG-Acr to PEG-VS over 4 d of culture (from nondegradable [ND-PEG] to fully degradable [100% D-PEG]). D) 4-arm LDTM 100% D-PEG (2.5%, 1×10^{-3} M RGD) permits efficient intestinal organoid formation. E) Immunostaining of Paneth cells (lysozyme), goblet cells (mucin-2), enterocytes (L-FABP), and enteroendocrine cells (chromogranin-A) in the formed organoids. All data are reported as mean ± S.D. ($n = 3$). Scale bars for bright-field images: 100 μm and for immunostaining images: 50 μm.

The LDTM hydrogel that we used in this study is biodegradable due to the presence of Metalloprotease (MMP)-sensitive peptides. We evaluated the biological effect of such biodegradability on embryo growth in a set of experiments below (Review Figure 3), where a non-biodegradable hydrogel leads to lower efficiency of embryo culture compared to the biodegradable hydrogel (Figure S1H). The biodegradability by the growing embryo is further confirmed with a consistent observation of trophoblast cell membrane invasion 10-80 um deep inside the matrix (Review Figure 6). We included this figure into the Supplementary Figure 1 (panel g) and modified the main text to refer to the above-mentioned analysis.

Figures for referees not shown.

5) Figures 2ef (in utero) and 2gh (3E-uterus) show rather different results. In uterus, pERM and ZO1 look quite compartmentalized in the outer region. This is not the case in 3E-uterus shown in Figure 2gh. These data do not seem to support an agreement between in utero and 3E-uterus as mentioned in the text.

Exactly. In this figure panel (Figure 2G, H), we aimed to illustrate the loss of trophoblast cell polarity when the adhesion to the uterine matrix is firmly established. Unfortunately, there was no experimental possibility to make such an observation *in utero* with the same clarity, as we could not distinguish the trophoblast and the uterine apical cell surfaces due to their close proximity and even intercalation of the latter into the trophoblast during phagocytosis. Hence, we analyzed the latest *in utero* point when the embryo can be recovered and, to avoid duplicating published findings, referred to the Kim et al., 2022 study for the same analysis at E3.5.

Kim et al., 2022, https://doi.org/10.1242/dev.200140.

6) The authors claim that mTE cells lose cell polarity upon adhesion to the uterine matrix and acquire mesenchymal properties. This claim should be clarified. Cells protrude and become migratory but invasion seems to be collective, suggestive that epithelial features such as cadherin adhesion remain. Are these cells mesenchymal or are they simply epithelial cells with motile capacity (as in wound healing, for example)? How do mesenchymal vs epithelial features compare between in utero and 3E-uterus?

We thank the reviewer for pointing the important validation of mesenchymal or epithelial trophoblast cell properties. The immunofluorescence of E- and N-cadherins at day 3 of 3E-uterus demonstrated that trophoblast cells lack N-cadherin and preserve weak E-cadherin expression, in contrast with a mesenchymal phenotype (Review Figure 4). We will additionally confirm this observation *in utero*, but we already corrected the manuscript and do not refer to the trophoblast motion as ‘mesenchymal’. In the context of collective motion, we would like to note that the majority of trophoblast cells remain adherent to the underlying Reichert’s lamina both *ex vivo* and *in utero* (Figure S1K), suggesting that such cell-ECM contact at the basal side could be responsible for collective motility.

Reviewer 3

Major

Claim 2: The release of tension from the polar side upon implantation allows for extra embryonic development.
To quantitatively measure the difference in tension before and after implantation is technically very challenging. However, this paper could benefit of further validations including IF stainings for markers such as E-cadherin, F-actin and Phospho myosin. In addition to this, treatments with Y27 and blebbistatin of the embryo would allow to further study the role of cell tension on embryo implantation. Finally, a laser ablation experiment at the cell junctions of the polar region before and after implantation would help to answer this question but this could be technically challenging due to the curvature nature of embryos.

We would like to clarify that when we talk about 'tension release', we mean a change of the tension at the interface between embryo and substrate, i.e., an increase of adhesion there, and not that there is a change in the tension at the embryo-medium surfaces.
The currently available techniques, such as micropipette aspiration, cannot yield a direct measurement in our setup due to: 1) mechanical inaccessibility because of the hydrogel, the upright orientation of the crypt, and 2) stickiness of mural TE for such micromanipulation (Ichikawa et al., 2022). As was suggested by the reviewer, an approximation for such tensions can be made by quantification of the relative double-phosphorylated myosin levels at the apical cell surfaces of mural and polar TE. In agreement with our model, the tension of the mural TE is decreased relative to the polar TE when embryo-matrix interaction is established (Review Figure 2d).

Claim 4: The spatial orientation of the embryo plays a key role in mouse peri implantation development.
In Figure 5i-j, the authors place embryos in a downward (i) and upward (j) orientation. Could the authors also please comment on whether they believe the orientation, the way the embryo feels the gravity plays a role in implantation?
Is the amount of space that the embryo has to grow in the limiting factor on development? Could the authors use 3E-uterus models with different lengths (by using molds with different spacing) to see the role of geometry and space that the embryo has for trophoblast mobility and embryo growth.
What would happen if the embryo were very close to the bottom of the hydrogel?

We thank the reviewer for raising this point, which is relevant to the proposed tissue coordination model. To address this question, in the experiment below (Review Figure 5), we manufactured shallow microwells and implanted embryos in a downward orientation, providing less space below the embryo. Live imaging showed that the downward elongation of the egg cylinder became restricted shortly after the trophoblast reached the microwell bottom. Such perturbation, however, did not fully terminate embryo growth as it continued upward and eventually outside the microwell. We note that such behavior is abnormal because, *in utero*, epiblast does not extend the upper edge of the cup formed by the Reichert's membrane, in agreement with the model, where space extension is required from the abembryonic (mural TE/trophoblast) side. We did not notice any substantial differences in the dynamics of such growth between the shallow microwell condition (Review Figure 5) and the counter-axial embryo orientation (Figure 5J). It is unclear, however, whether we can make any firm

assumptions about the significance of the relative influence of gravitational force on the embryonic and abembryonic embryo sides as well as how those can be perceived by different parts of the embryo.

Figures for referees not shown.

We have updated the manuscript with this experiment as the Supplementary Figure 8.

In this technical advance paper, the claims will become more robust with the suggested experiments above. Claims of implantation should be changed to accurately reflect that the 3E-uterus models peri implantation as there is no invasion in the 3D hydrogel matrix. In addition to this, the uterine cells are missing which are required to fully recapitulate the mechanisms of embryo peri-implantation.

We thank the reviewer for pointing out the aspect of invasion. We realized that although we frequently observed invasion of the hydrogel matrix, we did not sufficiently highlight it in the manuscript. Invasive trophoblast cell protrusions at least 10 μm deep inside the biodegradable LDTM PEG matrix are consistently observed in 86% of all WT embryos at the day 3 of 3E-uterus (Review Figure 6). This figure has been incorporated into the revised manuscript as a panel g of the Supplementary Figure 2.

Revision Plan

Figures for referees not shown.

`Minor comments:

- Specific experimental issues that are easily addressable.
1. Laminin staining in Figure 2A is only done in in vitro embryos but not in in vivo embryos. Could the authors add the missing staining?

Please refer to the Supplementary Figure 1K, where exactly this immunostaining is performed.

4. Can the authors comment on Figure Supp. 3D where the timing seems to be flipped?

We would like to clarify that the timing is correct and not flipped on that figure. We chose to show it in the reverse order to illustrate the change of shape dynamics according to the Figure Supp. 3C.

5. Why was 600 um chosen for the depth of the 3E-uterus?

The depth of the microwell was chosen to be: 1) sufficient to accommodate growth of the embryos up to the studied developmental stage, 2) robust to the range of blastocyst sizes, which affect how deep embryos can implant within the microwell, 3) feasible to manufacture with the methods available to us.

In figure 2E and 2G, the outline covers the staining. Would it be possible to have it without the outline in the supplementary?

Such figure without the outline was added to the Supplementary Figure 2H.

4. Description of analyses that authors prefer not to carry out

Please include a point-by-point response explaining why some of the requested data or additional analyses might not be necessary or cannot be provided within the scope of a revision. This can be due to time or resource limitations or in case of disagreement about the necessity of such additional data given the scope of the study. Please leave empty if not applicable.

Claim 3: Integrin-mediated adhesion between the trophoblast and the uterine matrix is required for in utero-like transition of the blastocyst to the egg cylinder.
In Figure 2a the authors show that embryos cultured in 3E-uterus without RGD do not develop and hypothesize this is due to lack of integrin binding. A control experiment using a non-integrin binding peptide is beneficial here.

From our experience with organoid systems, where integrin-mediated adhesion to the matrix is also required, the experiment with scrambled RDG peptide yields the same result as no addition of RGD. Scrambled RGD does not have any impact on survival of hMSCs cells and is similar to an inert PEG (Salinas & Anseth, 2008). The design and incorporation of other peptides will be beyond our current technical capabilities, and we do not consider an experiment with a scrambled peptide necessary for this study.

Salinas & Anseth, 2008, doi: 10.1002/term.95.

Dear Takashi,

Thank you for submitting your manuscript for consideration by the EMBO Journal. I have now read your manuscript, the reviewer comments and your response to them. Based on our editorial assessment and the referees' positive evaluations, I would like to invite you to submit a revised version of the manuscript along the lines indicated in your revision plan.

We generally allow three months as standard revision time. As a matter of policy, competing manuscripts published during this period will not negatively impact on our assessment of the conceptual advance presented by your study. However, please contact me as soon as possible upon publication of any related work to discuss the appropriate course of action. Should you foresee a problem in meeting this three-month deadline, please let us know in advance in order to arrange an extension.

When preparing your letter of response to the referees' comments, please bear in mind that this will form part of the Review Process File and will therefore be available online to the community. For more details on our Transparent Editorial Process, please visit our website: https://www.embopress.org/page/journal/14602075/authorguide#transparentprocess. Please also see the attached instructions for further guidelines on preparation of the revised manuscript.

Please feel free to contact me if you have any further questions regarding the revision. Thank you for the opportunity to consider your work for publication. I look forward to receiving your revised manuscript.

With best regards,

Ieva

Ieva Gailite, PhD
Senior Scientific Editor
The EMBO Journal
Meyerhofstrasse 1
D-69117 Heidelberg
Tel: +4962218891309
i.gailite@embojournal.org

Instructions for preparing your revised manuscript:

Please make sure you upload a letter of response to the referees' comments together with the revised manuscript.

Please also check that the title and abstract of the manuscript are brief, yet explicit, even to non-specialists.

When assembling figures, please refer to our figure preparation guideline in order to ensure proper formatting and readability in print as well as on screen:
https://bit.ly/EMBOPressFigurePreparationGuideline
See also figure legend guidelines: https://www.embopress.org/page/journal/14602075/authorguide#figureformat

At EMBO Press we ask authors to provide source data for the main manuscript figures. Our source data coordinator will contact you to discuss which figure panels we would need source data for and will also provide you with helpful tips on how to upload and organize the files.

IMPORTANT: When you send the revision we will require
- a point-by-point response to the referees' comments, with a detailed description of the changes made (as a word file).
- a word file of the manuscript text.
- individual production quality figure files (one file per figure)
- a complete author checklist, which you can download from our author guidelines (https://www.embopress.org/page/journal/14602075/authorguide).
- Expanded View files (replacing Supplementary Information)
Please see out instructions to authors
https://www.embopress.org/page/journal/14602075/authorguide#expandedview

Please remember: Digital image enhancement is acceptable practice, as long as it accurately represents the original data and conforms to community standards. If a figure has been subjected to significant electronic manipulation, this must be noted in the

figure legend or in the 'Materials and Methods' section. The editors reserve the right to request original versions of figures and the original images that were used to assemble the figure.

Further information is available in our Guide For Authors: https://www.embopress.org/page/journal/14602075/authorguide

We realize that it is difficult to revise to a specific deadline. In the interest of protecting the conceptual advance provided by the work, we recommend a revision within 3 months (16th Mar 2023). Please discuss the revision progress ahead of this time with the editor if you require more time to complete the revisions. Use the link below to submit your revision:

Link Not Available

***
Rev_Com_number: RC-2022-01701
New_manu_number: EMBOJ-2022-113280
Corr_author: Hiiragi
Title: EMBRYO-UTERINE INTERACTION COORDINATES MOUSE EMBRYOGENESIS DURING IMPLANTATION

Full Revision

Manuscript number: RC-2022-01701
Corresponding author(s): Takashi Hiiragi

1. General Statements [optional]

We greatly thank all three referees for constructive comments for our manuscript, appreciating the technical advances of our study, high-resolution live imaging, data quality, and highly interdisciplinary work. The substantial challenges and limitations for the hypothesis-driven study of embryo implantation in utero motivated us to undertake a bottom-up approach, where the complexity of the system can be decomposed to a finite number of controllable – in this case, biomechanical - parameters. The present study marks the beginning of mechanistic understanding of a complex interaction between the embryo and the maternal uterine environment using ex vivo engineering, live imaging, and biophysical modeling. As was pointed out by the referees, we envision that the next generations of methods will increase the complexity to unleash new aspects of embryo-maternal interactions and feedback mechanisms across the scales of this fascinating biological system.

As described in the following section, we addressed all comments by the reviewers and successfully carried out necessary experiments and analyses. These substantially improved the quality of our study, and we hope that this study is now suitable for publication in EMBO Journal.

2. Point-by-point description of the revisions

Reviewer #1 (Evidence, reproducibility and clarity (Required)):
Summary:
To recapitulate mouse peri-implantation development ex vivo, the authors engineered a uterus-like microenvironment by fabrication of topographically patterned hydrogels and identified the roles of the physical interaction between embryo and hydrogels for egg cylinder formation. Notably, integrin-mediated adhesion between trophoblast and matrix facilitates egg-cylinder shape. Moreover, Live-imaging with light-sheet microscopy led them to propose the hypothesis that the interaction between embryos and hydrogels appeared to be described by a droplet wetting process.

Major comments:
Although the authors claim that the interaction between the uterus and embryo is crucial for egg-cylinder formation, they did not utilize uterus-derived cells nor analyze these. They just observed how blastocysts grow autonomously into the egg-cylinder shape in the hydrogel which has solely physical properties of the uterus but not biochemical features except for the RGD peptide-mediated cell adhesion process. Thus, it is still uncertain if similar mechanisms contribute to egg-cylinder formation in utero. To fulfill the gap between ex vivo morphogenesis and in utero, the authors would be expected to analyze the interaction between trophoblast and uterus in utero if uterine

mechanisms can follow the integrin-mediated adhesion and a droplet wetting process. For example, whether integrin can contribute to egg-cylinder formation in utero can be proved by analyzing knock-out phenotypes of integrin-related genes. It will take around six months to conduct such suggested experiments. Otherwise, the authors should modify their statement "the interaction between embryo and uterine" into "the interaction between embryos and uterine-like hydrogels" throughout the manuscript.

This is an important point raised by several reviewers. To address the *in vivo* relevance of our findings, we have analyzed the Integrin beta 1 subunit knockout embryos for their implantation phenotype and morphology *in utero* at the onset of implantation. The embryos were differentially labeled from the surrounding maternal tissues with an endogenously expressed fluorescent membrane protein provided by a double-transgenic male, $mTmG^{(hom)}/Itgb1^{+/-}$ and analyzed with immunofluorescence of the pregnant uterine tissue sections from $Itgb1^{+/-}$ female mice at E4.5 and E4.75. The results are presented in the Supplemental Figure 6 and explained in the main text.

Moreover, we also examined *in utero* morphology of Rac1 knockout embryos at E5.25, when the egg cylinder growth becomes more evident. As much as the whole-embryo knock out of Rac1 can be informative, our finding of the retarded embryo growth *in utero* (see Supplemental Figure 11 and the main text) is in agreement with the results *ex vivo* (Fig. 6f – h). While we acknowledge that the tools to directly address the questions *in utero* are still limited, the agreement with *in utero* observations allows us to get, perhaps, the best currently available evidence of *in vivo* relevance.

Specific point:
Supplemental figure 1h, page 4 lines 20-23:
The authors claim that 1.5-2 % PEG generated the shear modulus at 100-300 Pa, which is in the stiffness range of the E5.5 mouse decidua (Govindasamy et al., 2021).
In Govindasamy's paper, elasticity measurements were performed in Petri dishes using an MFP-3D Classic AFM (Asylum Research, Wiesbaden, Germany) and cantilevers with a force constant of 0.08 with spherical tips (2 mm; NanoWorld, Neuchatel, Switzerland). In the present paper, the shear modulus (G′) of hydrogels was determined by performing small-strain oscillatory shear measurements on a Bohlin CVO 120 rheometer with plate-plate geometry. Therefore, it is not appropriate that Govindasamy's modulus is compared to the authors' modulus directly. For a direct comparison of the two modulus values, the authors can measure the stiffness of PEG with AFM or the shear modulus of the E5.5 mouse decidua by their rheometer.

Reviewer #1 makes a valid point about the differences in methods used for stiffness measurements and questions the appropriateness of a comparison between hydrogel stiffness in our study and mouse decidual stiffness in Govindasamy et al. 2021. The relationship between the shear modulus (G) and elastic modulus (E) of hydrogels are generally considered to be related by the Trouton ratio $E = 3G$ (simplification of $E = 2G\ (1+v)$, assuming $v = 0.5$, where v is the Poisson ratio for soft incompressible materials). We note, however, that at lower crosslinking degrees and hence larger mesh sizes and lower stiffnesses, modulus values determined from AFM and bulk rheology data indeed start to diverge, with AFM commonly generating higher moduli compared to bulk rheology. For example, in Abrego et al., 2022, the authors compared elastic and shear moduli of PEG (8-arm Vs with DTT, similar to conventional PEG-PEP gels in

terms of network architecture, and softer than LDTM gels) and Fibrin gels, illustrating that low concentrations of PEG (2.5% W/V) led to larger E/G' ratios (i.e., up to 8.0) and therefore a more non-affine network. As LDTM PEGs contain lower structural defects compared to conventional PEGs (Rezakhani et al., 2020), and therefore have higher elastic modulus at lower PEG content, we cautiously motivated relating the stiffness measurements by using the E = 2G (1+v) relationship, assuming v = 0.5, where v is the Poisson ratio for soft incompressible materials. Figure 2D of Govindasamy et al, 2021 shows the elastic modulus of the decidual tissue as 400 Pa, which can be comparable to the 100-200Pa shear modulus of our LDTM PEG used in the study.

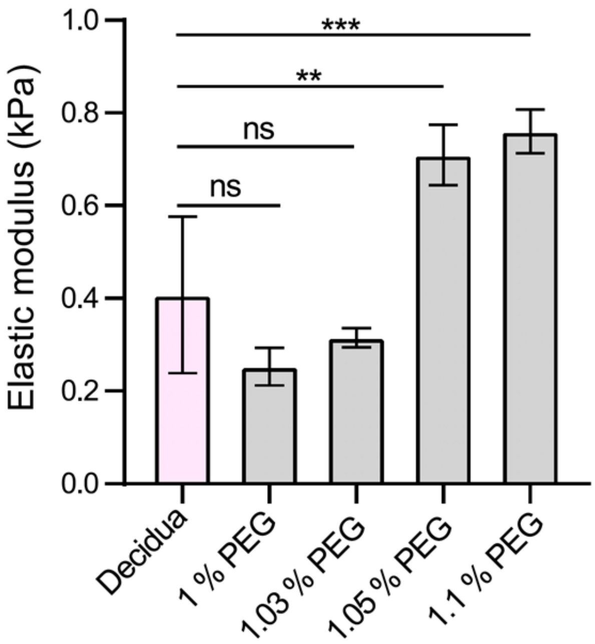

Figure 2D from Govindasamy et al, 2021.

Abrego et al., 2022, https://doi.org/10.1002/macp.202100366
Govindasamy et al, 2021, https://doi.org/0.1016/j.devcel.2021.10.014
Rezakhani et al., 2020, https://doi.org/10.1002/adfm.202000761

Reviewer #2 (Evidence, reproducibility and clarity (Required)):

The authors developed a new method of ex vivo culture of mammalian embryos. This engineered uterus recapitulates some features of peri-implantation development of the mouse embryo. The authors show that integrin adhesion to the uterine wall through integrin beta 1 is required for proper peri-implantation. They also demonstrate collective migration of the trophoblast on the synthetic hydrogel surface. The authors interpret their results through the physics of wetting, which allows them to conclude that a release of tension enables shape changes in the embryo. Finally, light sheet imaging allows the authors to visualize the interplay between growth and collective motion.

Major
1) The article will be of potential interest to a broader community than mammalian embryo peri-implantation researchers. This broader community will likely not be familiar with the structure and nomenclature of the embryo and surrounding tissues. The introduction of terms in the second paragraph of the introduction should be paralleled by a comprehensive image in Fig. 1. This image should clarify what is considered apical and basal in this context. Similarly, when the model is introduced a more comprehensive scheme should also be provided.

We thank the reviewer for suggesting improving the manuscript's clarity for a broader audience. Following the reviewer's suggestion, we added the requested schematic as a Supplemental Fig. 1a. Moreover, we also made an explanatory video of the revised paragraph of the introduction section to familiarize better the reader with the structure, nomenclature of the embryo lineages, and the surrounding tissues in our best manner. We have corrected the nomenclature related to the model throughout the manuscript and removed the terms which might be confusing, such as 'apical' and 'basal'. In the context of apicobasal polarity of the mural TE, we modified the schematics in Figure 4 c and i with the corresponding labels. The biophysical model schematic in the main Figure 4f was modified to include the missing volume parameter. We believe that this, along with the experimental data (Figure 4c and Figure 4i), and the more detailed schematics already presented in the Supplemental Fig. 6 should introduce all key model parameters and address the possible confusion.

2) The failure of 2D hydrogels to support mouse blastocysts through peri-implantation (Supp Fig. 1) is insufficiently described. Some panels in this figure and not mentioned in the main text. This discussion should be expanded, especially considering that 2D approaches has been quite successful. A detailed discussion of the authors' cylinder approach compared to the best 2D systems published should be provided (Govindasamy et al, for example).

We have corrected the reference to these panels in the main text and provided a better explanation of our approach in the context of the published methods, such as Bedzhov et. al., 2014, Govindasamy et al., 2021, and Ichikawa et al., 2022. Please see the manuscript main text.

Bedzhov et al., 2014, https://doi.org/10.1038/nprot.2014.186
Govindasamy et al., 2021, doi: 10.1016/j.devcel.2021.10.014.
Ichikawa et al., 2022, https://doi.org/10.1016/j.devcel.2021.12.023.

3) Is the hydrogel purely elastic or viscoelastic? The mechanical properties of the hydrogel (viscoelasticity and degradability) should be presented in the main text.

The LDTM hydrogel that we used in this study is biodegradable due to the presence of Metalloprotease (MMP)-sensitive peptides. We evaluated the biological effect of such biodegradability on embryo growth in a set of experiments below (Review Figure 1), where a non-biodegradable hydrogel leads to lower efficiency of embryo culture compared to the biodegradable hydrogel. These results were included into Supplemental Fig. 1g next to the other tested conditions. The biodegradability by the growing embryo agrees with a consistent observation of trophoblast cell membrane invasion 10-80 um deep inside the matrix (Supplemental Figure 5i).

Figures for referees not shown.

The mechanical properties of the Low-Defect Thiol-Michael (LDTM) PEG, which we used in this study, were also thoroughly characterized in our previous work (Rezakhani et al., 2020), which we refer to in the second part of our answer below. We also added a clarification in the main text with a corresponding citation.

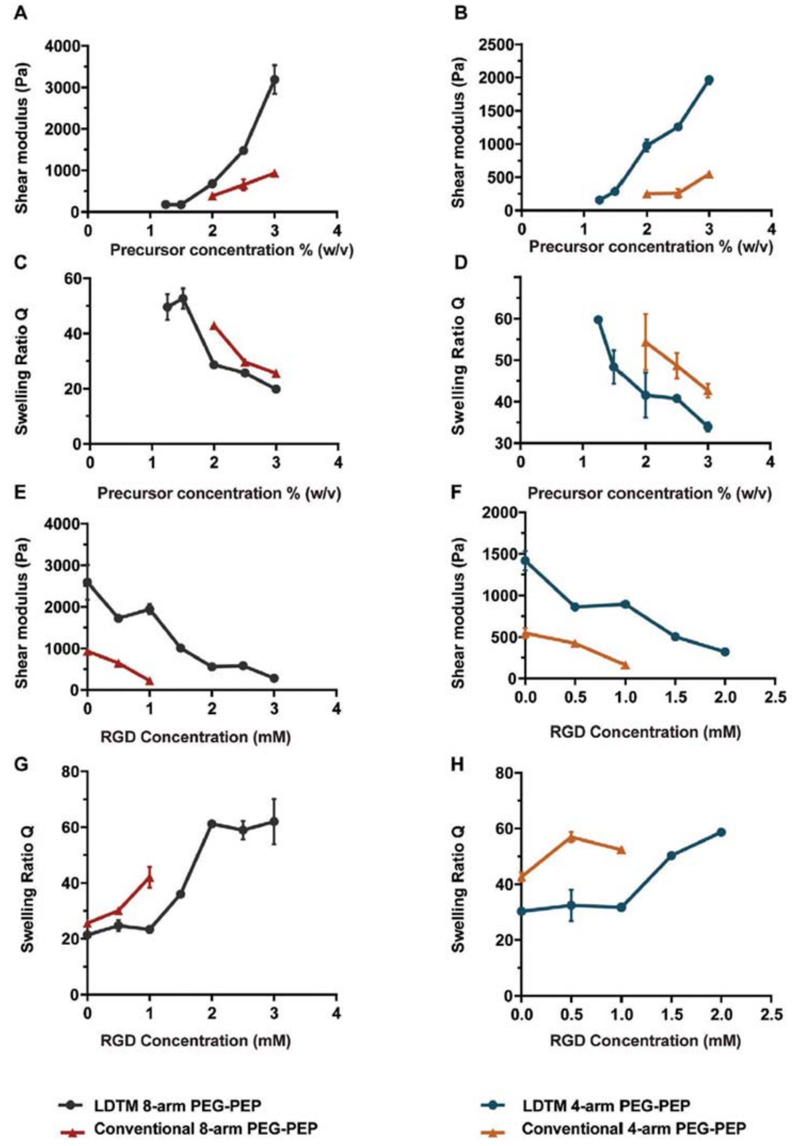

Figure 2. Network characterization. A,B) 4- and 8-arm LDTM hydrogels (no RGD) have a higher shear modulus and C,D) lower swelling ratios compared to conventional PEG-PEP gels. E–H) Shear moduli and swelling ratios of hydrogels formed at 3% (w/v) and at $r = 0.8$ as a function of RGD concentration. All data are reported as mean ± S.D. ($n = 3$).

In the figure above (Rezakhani et al., 2020), we characterized the elastic moduli and swelling ratios for different polymer contents and RGD concentrations. The effect of the stoichiometry of functional groups (or a number of elastically active chains) in conventional and LDTM hydrogels is also evaluated in Supplementary Figures 3 and 4 of the same study.

There, we also characterized the bulk degradation due to the hydrolysis of acrylate groups (Figure 4 below) by quantifying the elastic moduli and the biological effects on organoid growth.

Review COMMONS

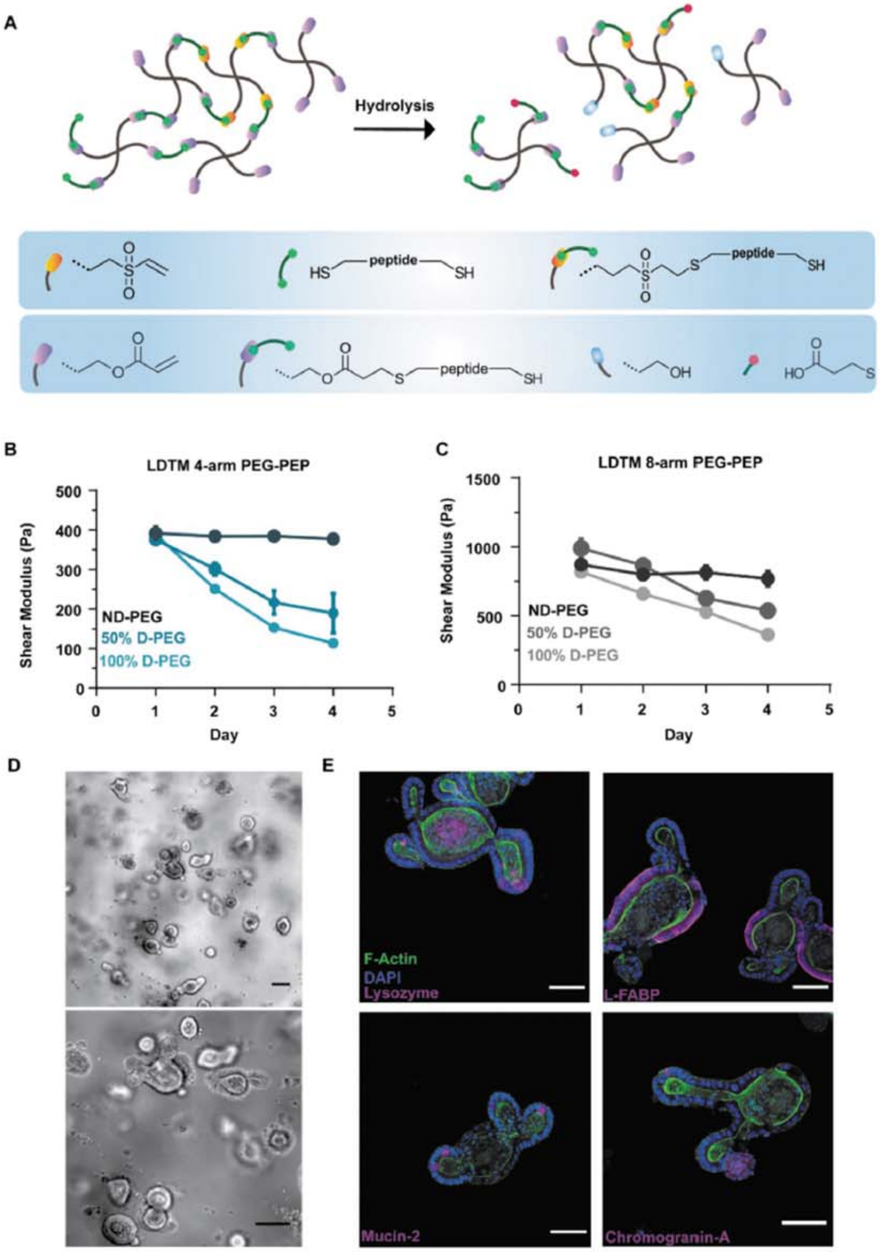

Figure 4. Dynamic matrices for organoid formation in one step. A) Schematic illustrating the ester hydrolysis of LDTM hydrogels containing peptide-functionalized PEG-Acr and PEG-VS macromers. B,C) Softening rate in 4- and 8-arm LDTM hydrogels (2.5%, 1×10^{-3} M RGD) with different ratios of PEG-Acr to PEG-VS over 4 d of culture (from nondegradable [ND-PEG] to fully degradable [100% D-PEG]). D) 4-arm LDTM 100% D-PEG (2.5%, 1×10^{-3} M RGD) permits efficient intestinal organoid formation. E) Immunostaining of Paneth cells (lysozyme), goblet cells (mucin-2), enterocytes (L-FABP), and enteroendocrine cells (chromogranin-A) in the formed organoids. All data are reported as mean ± S.D. ($n = 3$). Scale bars for bright-field images: 100 µm and for immunostaining images: 50 µm.

4) The authors call the finding that integrins are required for peri-implantation "striking", but a role for integrins in this process is are already known (see Sutherland et al, for example). The novelty of the authors findings in this regard should be better presented.

We thank the reviewer for raising this point, and modified the result and discussion sections accordingly. Although integrins were shown to be involved in implantation by the previous studies, the current study provides the mechanistic link between embryo-uterine adhesion and embryo morphogenesis. The latter is also extensively addressed here using biophysical modeling.

5) Figures 2ef (in utero) and 2gh (3E-uterus) show rather different results. In uterus, pERM and ZO1 look quite compartmentalized in the outer region. This is not the case in 3E-uterus shown in Figure 2gh. These data do not seem to support an agreement between in utero and 3E-uterus as mentioned in the text.

The reviewer is correct that this figure panel (Figure 3g, h) shows the loss of trophoblast cell polarity when the adhesion to the uterine matrix is firmly established. However, experimentally, it was impossible for us to make such an observation *in utero* as we could not distinguish the trophoblast and the uterine apical cell surfaces due to their close proximity and even intercalation of the latter into the trophoblast during phagocytosis. Hence, we analyzed the latest *in utero* point when the embryo can be recovered and, to avoid duplicating published findings, referred to the Kim et al., 2022 study for the same analysis at E3.5.

Kim et al., 2022, https://doi.org/10.1242/dev.200140.

6) The authors claim that mTE cells lose cell polarity upon adhesion to the uterine matrix and acquire mesenchymal properties. This claim should be clarified. Cells protrude and become migratory but invasion seems to be collective, suggestive that epithelial features such as cadherin adhesion remain. Are these cells mesenchymal or are they simply epithelial cells with motile capacity (as in wound healing, for example)? How do mesenchymal vs epithelial features compare between in utero and 3E-uterus?

We thank the reviewer for pointing the important validation of mesenchymal or epithelial trophoblast cell properties. The immunofluorescence of E- and N-cadherins at day 3 of 3E-uterus demonstrated that trophoblast cells lack N-cadherin and preserve weak E-cadherin expression, in contrast with a mesenchymal phenotype (Review Figure 2). Similarly, we did not observe N-cadherin expression in *in utero* trophoblast between E4.75 and E5.25, and corrected the manuscript not to refer to the trophoblast motion as ‘mesenchymal’ due to insufficient evidence. In the context of collective motion, we would like to note that the majority of trophoblast cells remain adherent to the underlying Reichert’s lamina both *ex vivo* and *in utero* (Supplemental Fig. 1k), suggesting that in addition to E-cadherin, such cell-ECM contact at the basal side could be responsible for a collective aspect of trophoblast motility.

Figures for referees not shown.

7) If I understand correctly, the model assumes that tension of the droplet-medium interface and is the same in the upper and lower sides of the embryo. However, the mechanical and geometrical properties of cells in both sides are quite different. Is the assumption of same tension justified? Can these tensions be measured or inferred to test this assumption?

8) Along similar lines, attributing a surface tension to a system that is thick (ie several cell layers) and that undergoes apical constriction (ie a bending modulus) is an oversimplification that should be justified. Cells in the pTE change (potentially) their apical, basal and lateral tension during apical constriction. How do these three components relate to what the model simply refers as tension? Additionally, how does the presence of a bending moment alter the wetting picture?

9) The physics of wetting were recently generalized to include additional terms attributed to active components (main associated with polarity, see works by Alert and Casademunt). These active components are not explicitly taken into account in the authors' model. Are they not needed? A brief discussion of this aspect should be provided.

Response to points 7 – 9:

Our goal was to find a mechanical description, which is sufficiently simple to make a quantitative match with the experimental observables to identify the key drivers of embryo shape dynamics during implantation. Indeed, the quantitative agreement between our simple model and the experimentally measured contact angles in both EPI-proximal (top) and EPI-distant (bottom) parts of the embryo suggests that the predominant driving mechanism underlying embryo shape dynamics in 3E-uterus is—in addition to volume changes—an increase in adhesion between TE cells and substrate. Given that the volume variation is known from experiments, our minimal model is well-constrained and permits the embryo-specific inference of the adhesion dynamics.

To further support the soundness of our model, we performed leave-one-out cross validation, and recovered that we can not only fit, but also predict the measured dynamics within some error bounds in almost all cases. We have included these results in the manuscript as a supplementary figure (Supplemental Fig. 8).

To address the Referee's points more specifically, we added a more detailed account of our rationale and biophysical aspects to the manuscript. In response to the inquiry about active components, bending moduli and other aspects we first remark that various *microscopic* mechanisms may give rise to collective phenomena described at a *macroscopic* level by the same thermodynamic model—an advantage which makes thermodynamic models widely applicable. The downside of this approach is that in order to uncover the molecular mechanisms, one must use other tools.

That being said, our model clearly requires active mechanisms: a time-dependent tension and volume are not possible without such components. As shown in Supplementary Note [see Eqs. (S20)–(S22)], we derive how these active components do a thermodynamic work on the system.

Bending moduli, tissue structure, and cellular processes certainly affect how the system's volume and shape evolve in time and should be taken into account, when one *explicitly* models the volume dynamics. At present a realistic modeling of such processes seems to us unfeasible. To avoid it altogether we use directly the *empirical* time-series of volume changes measured in experiments (see Supplementary Note), whereas the shape of the system is naturally constrained. Therefore, the microscopic properties, such as those listed here, are not relevant in our approach.

The microscopic origins of surface tension may be even more complicated than the bulk parameters: the interface properties depend on the coupling between both the environment and the system (i.e. *in utero* and embryo). Still the thermodynamic (macroscopic) definition of a surface tension does not depend on whether the system is in a liquid or solid state, or has a complex structure: it merely assumes a constitutive relation between the surface energy and area.

In the Review Figure 3, we show quantifications of the relative double-phosphorylated myosin levels at the apical cell surfaces of mural and polar TE, to approximate the relative difference in tension. Mural TE cells have higher ppMyo levels than polar TE (Review Figure 3b, d). Although this result is insufficient to conclude that the surface tensions are equal, we also did not observe a substantial difference and, hence, a clear justification for increasing the complexity of the model, in addition to the reasons listed above.

Figures for referees not shown.

10) In utero, the cavity in which the embryo is implanted is created during implantation. In this situation, the analogy with wetting seems harder to establish because the embryo spreads as it forms the cavity. How does this alter the authors interpretation?

This is an excellent point. On the basis of our findings here, we might propose that implantation into the uterine wall *in vivo* can be understood as a soft wetting problem (Andreotti and Snoeijer, 2020), i.e. where a droplet interacts with and deforms a soft substrate. Although addressing the dynamic nature of embryo-uterine interaction is beyond the scope of the current study, we elaborated our discussion of this interesting aspect.

Andreotti and Snoeijer, 2020, https://doi.org/10.1146/annurev-fluid-010719-060147.

11) The first paragraph of the supplementary note refers to Fig. 4D. This reference here does not seem correct.

We have corrected the reference in the supplementary note.

Reviewer #3 (Evidence, reproducibility and clarity (Required)):

Summary:
Provide a short summary of the findings and key conclusions (including methodology and model system(s) where appropriate).
In this paper the authors develop an engineered uterus-like microenvironment to recapitulate peri-implantation development of the whole mouse embryo ex vivo. This new model (3E-uterus) is used for mechanistic studies of embryo implantation. They hint that integrin-mediated adhesion of the embryo to the uterine wall is required for peri-implantation mouse development. The authors use this model also to study the role of tension for embryo development. They postulate that release of tension from the polar side of the embryo upon implantation allows for extra embryonic development. By using mathematical modeling of the implanting embryo as a wetting droplet, the authors link the embryo shape dynamics to the underlying changes in trophoblast adhesion and suggests that the adhesion-mediated tension release facilitates egg cylinder formation. Finally, the authors uncover the role of coordination between trophoblast motility and embryo growth, where trophoblast mobility displaces the Reichart's membrane giving the embryo space to grow. In summary, the authors technically advance the field of developmental biology by providing a model to study peri implantation morphogenesis of the mouse embryo.

Major comments:

- Are the key conclusions convincing?
The key conclusions the authors derive from their experiments are somewhat convincing. Suggested experiments below will strengthen their claims.

- Would additional experiments be essential to support the claims of the paper? Request additional experiments only where necessary for the paper as it is, and do not ask authors to open new lines of experimentation.

Claim 1: The 3E-uterus is representative of mouse embryo peri-implantation.
To claim this a more extensive validation of the embryos cultured in their 3E uterus both via scRNA seq and IF for pluripotency, visceral and parietal endoderm markers is required.
It is also interesting that embryos cultured in the 3E-uterus lose the correct timing of development. Could the authors please comment on this? scRNA seq of the embryos cultured in their system at different timepoint (i.e. Day 1-3) compared to control pre, peri and post implantation embryos could help answer this question.

Accordingly, we have performed scRNA-seq and extended IF analysis for pluripotency markers (Fig. 2, and Supplemental Figs. 2 — 4). *The raw data under GSE228264 is available with a review token: aforkyawphghlkt.*

We agree that it is interesting to find that 3E-uterus embryos reach the E4.5 stage in two days instead of one day of *in utero* development. The early developmental delay was also observed in the previous *ex vivo* studies (Bedzhov et al., 2014; Govindasamy et al., 2021), suggesting that additional cues might be required to enable timely adaptation of the trophectoderm to the *ex vivo* environment. Uterine endometrium provides a complex composition of adhesive molecules before the embryo reaches the underlying ECM (Dey et al., 2004). Future incorporation of cellular components into the

Review COMMONS

current platform may facilitate understanding of the role of cell-cell interactions and signaling between the embryo and the uterus. The present methods provide a powerful experimental platform for such mechanistic studies in the future. In perspective, they can help understanding the functional cross-talk between the uterine tissue dynamics, embryo morphology, and size.

Bedzhov et al., 2014, https://doi.org/10.1038/nprot.2014.186
Govindasamy et al, 2021, https://doi.org/0.1016/j.devcel.2021.10.014
Dey et al., 2004, doi: 10.1210/er.2003-0020

Claim 2: The release of tension from the polar side upon implantation allows for extra embryonic development.
To quantitatively measure the difference in tension before and after implantation is technically very challenging. However, this paper could benefit of further validations including IF stainings for markers such as E-cadherin, F-actin and Phospho myosin. In addition to this, treatments with Y27 and blebbistatin of the embryo would allow to further study the role of cell tension on embryo implantation. Finally, a laser ablation experiment at the cell junctions of the polar region before and after implantation would help to answer this question but this could be technically challenging due to the curvature nature of embryos.

We would like to clarify that when we talk about ‘tension release’, we mean a change of the tension at the interface between embryo and substrate, i.e., an increase of adhesion there, and not that there is a change in the tension at the embryo-medium surfaces.

The currently available techniques, such as micropipette aspiration, cannot yield a direct measurement in our setup due to: 1) mechanical inaccessibility because of the hydrogel, the upright orientation of the crypt, and 2) stickiness of mural TE for such micromanipulation (Ichikawa et al., 2022). As was suggested by the reviewer, an approximation for such tensions can be made by quantification of the relative double-phosphorylated myosin levels at the apical cell surfaces of mural and polar TE.

In the Review Figure 3, we show quantifications of the relative double-phosphorylated myosin levels at the apical cell surfaces of mural and polar TE, to approximate the relative difference in tension. Mural TE cells have higher ppMyo levels than polar TE (Review Figure 3b, d). Although this result is insufficient to conclude that the surface tensions are equal, we also did not observe a striking difference and, hence, a clear justification for increasing the complexity of the model.

Figures for referees not shown.

Our goal was to find a mechanical description, which is sufficiently simple to make a quantitative match with the experimental observables to identify the key drivers of embryo shape dynamics during implantation. Indeed, the quantitative agreement between our simple model and the experimentally measured contact angles in both EPI-proximal (top) and EPI-distant (bottom) parts of the embryo suggests that the predominant driving mechanism underlying embryo shape dynamics in 3E-uterus is—in addition to volume changes—an increase in adhesion between TE cells and substrate. Given that the volume variation is known from experiments, our minimal model is well-constrained and permits the embryo-specific inference of the adhesion dynamics.

To further support the soundness of our model, we performed leave-one-out cross validation, and recovered that we can not only fit, but also predict the measured dynamics within some error bounds

in almost all cases. We have included these results in the manuscript as a supplementary figure (Supplemental Fig. 8).

Claim 4: The spatial orientation of the embryo plays a key role in mouse peri implantation development.
In Figure 5i-j, the authors place embryos in a downward (i) and upward (j) orientation. Could the authors also please comment on whether they believe the orientation, the way the embryo feels the gravity plays a role in implantation?
Is the amount of space that the embryo has to grow in the limiting factor on development? Could the authors use 3E-uterus models with different lengths (by using molds with different spacing) to see the role of geometry and space that the embryo has for trophoblast mobility and embryo growth. What would happen if the embryo were very close to the bottom of the hydrogel?

We thank the reviewer for raising this point, which is relevant to the proposed tissue coordination model. To address this question, in the experiment shown in Supplemental Fig. 14, we manufactured shallow microwells and implanted embryos in a downward orientation, providing less space below the embryo. Live imaging showed that the downward elongation of the egg cylinder became restricted shortly after the trophoblast reached the microwell bottom. Such perturbation, however, did not fully terminate embryo growth as it continued upward and eventually outside the microwell. We note that such behavior is abnormal because, *in utero*, epiblast does not extend the upper edge of the cup formed by the Reichert's membrane, in agreement with the model, where space extension is required from the abembryonic (mural TE/trophoblast) side. We did not notice any substantial differences in the dynamics of such growth between the shallow microwell condition (Supplemental Fig. 14) and the counter-axial embryo orientation (Figure 6j).

Regarding the second point, it is unclear whether we can make any firm assumptions about the significance of the relative influence of gravitational force on the embryonic and abembryonic embryo sides as well as how those can be perceived by different parts of the embryo.

Claim 5: A mathematical model based on the wetting droplet recapitulates the embryo in their system.
Could the authors comment on whether their mathematical model considers proliferation, and would proliferation have an impact on the system's kinetics? What is the role of polar TE proliferation and how does that influence the trophectoderm morphology? If the embryo is geometrically confined, can the authors exclude that this confinement is influencing cell shape?

Our model fully accounts for the volume dynamics of the embryo throughout implantation, because we *directly* use the empirical time series from experiments. The volume variation is dominated by the inflation-collapse dynamics of the blastocoel (Chan et al., 2019) and not proliferation—the analysis of live imaging data indicated that mural TE cells rarely divide (12% of mTE/TB cell nuclei divided during 24 hours of live imaging; n=158), but any effect of proliferation is captured with our volume term. We have added a clarification of this point to the manuscript.

Chan et al., 2019, doi: 10.1038/s41586-019-1309-x.

Review COMMONS

In this technical advance paper, the claims will become more robust with the suggested experiments above. Claims of implantation should be changed to accurately reflect that the 3E-uterus models peri implantation as there is no invasion in the 3D hydrogel matrix. In addition to this, the uterine cells are missing which are required to fully recapitulate the mechanisms of embryo peri-implantation.

We thank the reviewer for pointing out the aspect of invasion. We realized that although we frequently observed invasion of the hydrogel matrix, we did not sufficiently highlight it in the manuscript. Invasive trophoblast cell protrusions at least 10 μm deep inside the biodegradable LDTM PEG matrix are consistently observed in 86% of all WT embryos at the day 3 of 3E-uterus (Supplemental Fig. 5i).

Minor comments:

- Specific experimental issues that are easily addressable.
1. Laminin staining in Figure 2A is only done in in vitro embryos but not in in vivo embryos. Could the authors add the missing staining?

Please refer to the Supplemental Fig. 1k, where exactly this immunostaining is performed comparing *in utero* vs *ex vivo*.

2. It would be beneficial to have both active and normal integrin stainings in E4.5 embryos.

Such immunostainings were included in the Supplementary Fig. 5a–b.

3. Could the authors provide stainings for mesenchymal markers for E4.5 and D2 3E uterus?

The immunofluorescence of E- and N-cadherins demonstrated that trophoblast cells lack N-cadherin and preserve weak E-cadherin expression, in contrast with a mesenchymal phenotype (Review Figure 2). Similarly, we did not observe N-cadherin expression in *in utero* trophoblast between E4.75 and E5.25, and corrected the manuscript not to refer to the trophoblast motion as ‘mesenchymal’.

Figures for referees not shown.

4. Can the authors comment on Figure Supp. 3D where the timing seems to be flipped?

We would like to clarify that the timing is correct and not flipped on that figure. We chose to show it in the reverse order to illustrate the change of shape dynamics according to the Supplemental Fig. 7c (updated).

5. Why was 600 um chosen for the depth of the 3E-uterus?

The depth of the microwell was chosen to be: 1) sufficient to accommodate growth of the embryos up to the studied developmental stage, 2) robust to the range of blastocyst sizes, which affect how deep embryos can implant within the microwell, 3) feasible to manufacture with the currently available methods.

In figure 2E and 2G, the outline covers the staining. Would it be possible to have it without the outline in the supplementary?

Such figure without the outline was added to the Supplemental Fig. 5j, k.

Review COMMONS

It would be very informative to have for each panel in which a representative image is used for that image to be marked into the quantified data (graphs).

We have modified the relevant figure panels accordingly.

How do the findings in this paper relate to the findings in Weberling et al (PMID 33472064), where they show that in vivo, the polar trophectoderm exerts physical force upon the epiblast, causing it to transform from an oval into a cup shape?

Our findings do not contradict the observations presented in Weberling et al., but the lack of direct physical measurements of the forces exhibited by the polar trophectoderm (pTE) and/or modeling and explicit experimental testing of the external pressure on the epiblast shape prohibits us from further conclusions about the role of the physical forces exerted by the pTE in the acquisition of the egg cylinder shape.

1st Revision - Editorial Decision 6th Jun 2023

Dear Takashi,

Thank you for submitting your revised Review Commons manuscript to The EMBO Journal. I sincerely apologise for the protracted handling process of your manuscript due to holidays here in Germany and the resulting backlog. Your manuscript has now been seen by all original reviewers, who find that their main concerns have been addressed and now recommends publication of the manuscript. I will therefore be happy to accept the manuscript for publication in The EMBO Journal after its reformatting as indicated below.

1. Our publisher has done their pre-publication check on your manuscript. I have attached the file here. Please take a look at the word file and the comments regarding the figure legends and respond to the issues.
2. Please limit the number of keywords to five.
3. Please check that the funding information is correct and identical both in the manuscript and our online system.
4. Please remove figures from the manuscript text file, while assembling the figure legends after the "References" section.
5. Please rearrange the order of the sections in the manuscript as follows: abstract, introduction, results, discussion, materials & methods, data availability section, acknowledgments, disclosure statement and competing interests, references, main figure legends, tables, expanded view figure legends.
6. Please make sure that all figure panels are called out and mentioned in the alphabetical order in the manuscript text.
7. We can accommodate up to five Expanded View (EV) Figures that are collapsible/expandable online. EV Figures should be cited as 'Figure EV1, Figure EV2" etc. in the text and their respective legends should be included in the main text after the legends of regular figures. Further information on the format is available here: https://www.embopress.org/page/journal/14602075/authorguide#expandedview.
8. The rest of the supplementary figures should be removed from the main manuscript file, renamed into Appendix Figure S1 etc., and assembled together with the rest of the supplementary information into a single Appendix pdf file that is prefaced by a short table of contents.
9. Please rename movies into Movie EV1 etc, remove the legends from the main manuscript file and zip as a README text file together with the corresponding movie file.
10. CRediT has replaced the traditional author contributions section because it offers a systematic, machine-readable author contributions format that allows for more effective research assessment. Please remove the Authors Contributions from the manuscript and use the free text boxes beneath each contributing author's name in our online submission system to add specific details on the author's contribution. More information is available in our guide to authors.
11. Please rename "Conflict of interest" section into "Disclosure and competing interests statement" (further info: https://www.embopress.org/page/journal/14602075/authorguide#conflictsofinterest).
12. Please update the references according to The EMBO Journal style (up to 10 authors followed by et al.) Further information can be found here: https://www.embopress.org/page/journal/14602075/authorguide#referencesformat
13. Please label the table in the Materials and Methods, Hydrogel formation section as Table 1.
14. Papers published in The EMBO Journal are accompanied online by a 'Synopsis' to enhance discoverability of the manuscript. Please submit synopsis text that consists of A) a short (1-2 sentences) summary of the findings and their significance, B) 3-4 bullet points highlighting key results and C) a synopsis image that is 550x300-600 pixels large (width x height, jpeg or png format). You can either show a model or key data in the synopsis image. Please note that the image size is rather small and that text needs to be readable at the final size.

Please feel free to contact me if you have any questions regarding this final editorial revision. Please use the link below to upload the revised files.

Thank you for the opportunity to consider your work for publication, and I look forward to receiving your revised manuscript.

With best wishes,

Ieva

Ieva Gailite, PhD
Senior Scientific Editor
The EMBO Journal
Meyerhofstrasse 1
D-69117 Heidelberg
Tel: +4962218891309
i.gailite@embojournal.org

Further information is available in our Guide For Authors: https://www.embopress.org/page/journal/14602075/authorguide

We realize that it is difficult to revise to a specific deadline. In the interest of protecting the conceptual advance provided by the work, we recommend a revision within 3 months (4th Sep 2023). Please discuss the revision progress ahead of this time with the editor if you require more time to complete the revisions. Use the link below to submit your revision:

https://emboj.msubmit.net/cgi-bin/main.plex

Referee #1:

The authors have addressed my comments with additional data and text edits. Even if some aspects of the model might be oversimplified, I appreciate the authors goal of providing a basic framework on which to build up in the future. I support publication of the article.

Referee #2:

We congratulate the authors for their excellent revised manuscript, and we recommend publication.
We have one last minor comment to be clarified:
Could the authors explain the difference between their model based on droplet wetting (considered as passive wetting) and active wetting?

Referee #3:

The authors have addressed my concerns raised previously. All additional data further support their conclusion. I have no additional comments. I can recommend publication.

All editorial and formatting issues were resolved by the authors.

Dear Takashi,

Thank you for addressing the final editorial points. I sincerely apologise for the delay in communicating the decision due to the high number of submissions we receive at the moment. I am now pleased to inform you that your manuscript has been accepted for publication.

Before we forward your manuscript to our publishers, I will look into the synopsis text in the next few days and will let you know if any edits to the journal style are needed.

Please note that it is EMBO Journal policy for the transcript of the editorial process (containing referee reports and your response letter) to be published as an online supplement to each paper. If you do NOT want this, you will need to inform the Editorial Office via email immediately. More information is available here:
https://emboj.embopress.org/about#Transparent_Process

Your manuscript will be processed for publication in the journal by EMBO Press. Manuscripts in the PDF and electronic editions of The EMBO Journal will be copy edited, and you will be provided with page proofs prior to publication. Please note that supplementary information is not included in the proofs.

Please note that you will be contacted by Wiley Author Services to complete licensing and payment information. The 'Page Charges Authorization Form' is available here: https://www.embopress.org/pb-assets/embo-site/tej_apc.pdf

EMBO Press participates in many Publish and Read agreements that allow authors to publish Open Access with reduced/no publication charges. Check your eligibility: https://authorservices.wiley.com/author-resources/Journal-Authors/open-access/affiliation-policies-payments/index.html

Should you be planning a Press Release on your article, please get in contact with embojournal@wiley.com as early as possible, in order to coordinate publication and release dates.

If you have any questions, please do not hesitate to call or email the Editorial Office. Thank you for this contribution to The EMBO Journal and congratulations on a successful publication!

Best regards,

Ieva

Ieva Gailite, PhD
Senior Scientific Editor
The EMBO Journal
Meyerhofstrasse 1
D-69117 Heidelberg
Tel: +4962218891309
i.gailite@embojournal.org

***

Rev_Com_number: RC-2022-01701
New_manu_number: EMBOJ-2022-113280R1
Corr_author: Hiiragi
Title: EMBRYO-UTERINE INTERACTION COORDINATES MOUSE EMBRYOGENESIS DURING IMPLANTATION

EMBO Press Author Checklist

Corresponding Author Name: Takashi Hiiragi	
Journal Submitted to: EMBO Journal	
Manuscript Number: RC-2022-01701 / EMBOJ-2022-113280	

USEFUL LINKS FOR COMPLETING THIS FORM
The EMBO Journal - Author Guidelines
EMBO Reports - Author Guidelines
Molecular Systems Biology - Author Guidelines
EMBO Molecular Medicine - Author Guidelines

Reporting Checklist for Life Science Articles (updated January
This checklist is adapted from Materials Design Analysis Reporting (MDAR) Checklist for Authors. MDAR establishes a minimum set of requirements in transparent reporting in the life sciences (see Statement of Task: 10.31222/osf.io/9sm4x). Please follow the journal's guidelines in preparing your
Please note that a copy of this checklist will be published alongside your article.

Abridged guidelines for figures
1. Data
The data shown in figures should satisfy the following conditions:
- the data were obtained and processed according to the field's best practice and are presented to reflect the results of the experiments in an accurate and unbiased manner.
- ideally, figure panels should include only measurements that are directly comparable to each other and obtained with the same assay.
- plots include clearly labeled error bars for independent experiments and sample sizes. Unless justified, error bars should not be shown for technical
- if n<5, the individual data points from each experiment should be plotted. Any statistical test employed should be justified.
- Source Data should be included to report the data underlying figures according to the guidelines set out in the authorship guidelines on Data

2. Captions
Each figure caption should contain the following information, for each panel where they are relevant:
- a specification of the experimental system investigated (eg cell line, species name).
- the assay(s) and method(s) used to carry out the reported observations and measurements.
- an explicit mention of the biological and chemical entity(ies) that are being measured.
- an explicit mention of the biological and chemical entity(ies) that are altered/varied/perturbed in a controlled manner.
- the exact sample size (n) for each experimental group/condition, given as a number, not a range;
- a description of the sample collection allowing the reader to understand whether the samples represent technical or biological replicates (including how many animals, litters, cultures, etc.).
- a statement of how many times the experiment shown was independently replicated in the laboratory.
- definitions of statistical methods and measures:
  - common tests, such as t-test (please specify whether paired vs. unpaired), simple $\chi 2$ tests, Wilcoxon and Mann-Whitney tests, can be unambiguously identified by name only, but more complex techniques should be described in the methods section;
  - are tests one-sided or two-sided?
  - are there adjustments for multiple comparisons?
  - exact statistical test results, e.g., P values = x but not P values < x;
  - definition of 'center values' as median or average;
  - definition of error bars as s.d. or s.e.m.

Please complete ALL of the questions below.
Select "Not Applicable" only when the requested information is not relevant for your study.

Materials

Newly Created Materials	Information included in the manuscript?	In which section is the information available? (Reagents and Tools Table, Materials and Methods, Figures, Data Availability Section)
New materials and reagents need to be available; do any restrictions apply?	Not Applicable	

Antibodies	Information included in the manuscript?	In which section is the information available? (Reagents and Tools Table, Materials and Methods, Figures, Data Availability Section)
For **antibodies** provide the following information:<br>- Commercial antibodies: RRID (if possible) or supplier name, catalogue number and or/clone number<br>- Non-commercial: RRID or citation	Yes	Materials and Methods, Key Resource Table

DNA and RNA sequences	Information included in the manuscript?	In which section is the information available? (Reagents and Tools Table, Materials and Methods, Figures, Data Availability Section)
**Short novel DNA or RNA including primers, probes**: provide the sequences.	Yes	Supplementary Table 1

Cell materials	Information included in the manuscript?	In which section is the information available? (Reagents and Tools Table, Materials and Methods, Figures, Data Availability Section)
**Cell lines:** Provide species information, strain. Provide accession number in repository **OR** supplier name, catalog number, clone number, and/**OR** RRID.	Not Applicable	
**Primary cultures:** Provide species, strain, sex of origin, genetic modification status.	Not Applicable	
Report if the cell lines were recently **authenticated** (e.g., by STR profiling) and tested for mycoplasma contamination.	Not Applicable	

Experimental animals	Information included in the manuscript?	In which section is the information available? (Reagents and Tools Table, Materials and Methods, Figures, Data Availability Section)
**Laboratory animals or Model organisms:** Provide species, strain, sex, age, genetic modification status. Provide accession number in repository **OR** supplier name, catalog number, clone number, **OR** RRID.	Yes	Materials and Methods
**Animal observed in or captured from the field:** Provide species, sex, and age where possible.	Not Applicable	
Please detail **housing and husbandry conditions**.	Yes	Materials and Methods

Plants and microbes	Information included in the manuscript?	In which section is the information available? (Reagents and Tools Table, Materials and Methods, Figures, Data Availability Section)
**Plants:** provide species and strain, ecotype and cultivar where relevant, unique accession number if available, and source (including location for collected wild specimens).	Not Applicable	
**Microbes:** provide species and strain, unique accession number if available, and source.	Not Applicable	

Human research participants	Information included in the manuscript?	In which section is the information available? (Reagents and Tools Table, Materials and Methods, Figures, Data Availability Section)
If collected and within the bounds of privacy constraints report on age, sex and gender or ethnicity for all study participants.	Not Applicable	

Core facilities	Information included in the manuscript?	In which section is the information available? (Reagents and Tools Table, Materials and Methods, Figures, Data Availability Section)
If your work benefited from core facilities, was their service mentioned in the acknowledgments section?	Yes	Acknowledgments

Design

Study protocol	Information included in the manuscript?	In which section is the information available? (Reagents and Tools Table, Materials and Methods, Figures, Data Availability Section)
If study protocol has been **pre-registered, provide DOI in the manuscript**. For clinical trials, provide the trial registration number **OR** cite DOI.	Not Applicable	
Report the **clinical trial registration number** (at ClinicalTrials.gov or equivalent), where applicable.	Not Applicable	

Laboratory protocol	Information included in the manuscript?	In which section is the information available? (Reagents and Tools Table, Materials and Methods, Figures, Data Availability Section)
Provide DOI OR other citation details if **external detailed step-by-step protocols** are available.	Yes	Materials and Methods; References

Experimental study design and statistics	Information included in the manuscript?	In which section is the information available? (Reagents and Tools Table, Materials and Methods, Figures, Data Availability Section)
Include a statement about **sample size** estimate even if no statistical methods were used.	Yes	Materials and Methods
Were any steps taken to minimize the effects of subjective bias when allocating animals/samples to treatment (e.g. **randomization procedure**)? If yes, have they been described?	Not Applicable	
Include a statement about **blinding** even if no blinding was done.	Yes	Materials and Methods
Describe **inclusion/exclusion criteria** if samples or animals were excluded from the analysis. Were the criteria pre-established?<br><br>If sample or data points were omitted from analysis, report if this was due to attrition or intentional exclusion and provide justification.	Yes	Materials and Methods
For every figure, are **statistical tests** justified as appropriate? Do the data meet the assumptions of the tests (e.g., normal distribution)? Describe any methods used to assess it. Is there an estimate of variation within each group of data? Is the variance similar between the groups that are being statistically compared?	Yes	Figure captions; Materials and Methods

Sample definition and in-laboratory replication	Information included in the manuscript?	In which section is the information available? (Reagents and Tools Table, Materials and Methods, Figures, Data Availability Section)
In the figure legends: state number of times the experiment was **replicated** in laboratory.	Yes	Figure captions; Materials and Methods
In the figure legends: define whether data describe **technical or biological replicates**.	Yes	Figure captions; Materials and Methods

Ethics

Ethics	Information included in the manuscript?	In which section is the information available? (Reagents and Tools Table, Materials and Methods, Figures, Data Availability Section)
Studies involving **human participants**: State details of **authority granting ethics approval** (IRB or equivalent committee(s), provide reference number for approval.	Not Applicable	
Studies involving **human participants**: Include a statement confirming that **informed consent** was obtained from all subjects and that the experiments conformed to the principles set out in the WMA Declaration of Helsinki and the Department of Health and Human Services Belmont Report.	Not Applicable	
Studies involving **human participants:** For publication of **patient photos**, include a statement confirming that consent to publish was obtained.	Not Applicable	
Studies involving experimental **animals**: State details of **authority granting ethics approval** (IRB or equivalent committee(s), provide reference number for approval. Include a statement of compliance with ethical regulations.	Yes	Materials and Methods
Studies involving **specimen and field samples:** State if relevant **permits** obtained, provide details of authority approving study; if none were required, explain why.	Not Applicable	

Dual Use Research of Concern (DURC)	Information included in the manuscript?	In which section is the information available? (Reagents and Tools Table, Materials and Methods, Figures, Data Availability Section)
Could your study fall under dual use research restrictions? Please check biosecurity documents and list of **select agents and toxins** (CDC): https://www.selectagents.gov/sat/list.htm	Not Applicable	
If you used a select agent, is the security level of the lab appropriate and reported in the manuscript?	Not Applicable	
If a study is subject to dual use research of concern regulations, is the name of the **authority granting approval and reference number** for the regulatory approval provided in the manuscript?	Not Applicable	

Reporting

The MDAR framework recommends adoption of discipline-specific guidelines, established and endorsed through community initiatives. Journals have their own policy about requiring specific guidelines and recommendations to complement MDAR.

Adherence to community standards	Information included in the manuscript?	In which section is the information available? (Reagents and Tools Table, Materials and Methods, Figures, Data Availability Section)
State if relevant guidelines or checklists (e.g., **ICMJE, MIBBI, ARRIVE, PRISMA**) have been followed or provided.	Not Applicable	
For **tumor marker prognostic studies,** we recommend that you follow the **REMARK** reporting guidelines (see link list at top right). See author guidelines, under 'Reporting Guidelines'. Please confirm you have followed these guidelines.	Not Applicable	
For **phase II and III randomized controlled trials**, please refer to the **CONSORT** flow diagram (see link list at top right) and submit the CONSORT checklist (see link list at top right) with your submission. See author guidelines, under 'Reporting Guidelines'. Please confirm you have submitted this list.	Not Applicable	

Data Availability

Data availability	Information included in the manuscript?	In which section is the information available? (Reagents and Tools Table, Materials and Methods, Figures, Data Availability Section)
Have **primary datasets** been deposited according to the journal's guidelines (see 'Data Deposition' section) and the respective accession numbers provided in the Data Availability Section?	Yes	Materials and Methods
Were **human clinical and genomic datasets** deposited in a public access-controlled repository in accordance to ethical obligations to the patients and to the applicable consent agreement?	Not Applicable	
Are **computational models** that are central and integral to a study available without restrictions in a machine-readable form? Were the relevant accession numbers or links provided?	Yes	Materials and Methods
If publicly available data were reused, provide the respective **data citations in the reference list**.	Yes	Materials and Methods
